# Identification of *Drosophila* Mutants Affecting Defense to an Entomopathogenic Fungus

**DOI:** 10.1038/srep12350

**Published:** 2015-07-23

**Authors:** Hsiao-Ling Lu, Jonathan B. Wang, Markus A. Brown, Christopher Euerle, Raymond J. St. Leger

**Affiliations:** 1 Department of Entomology, University of Maryland, College Park, Maryland, United States of America

## Abstract

Fungi cause the majority of insect disease. However, to date attempts to model host–fungal interactions with *Drosophila* have focused on opportunistic human pathogens. Here, we performed a screen of 2,613 mutant *Drosophila* lines to identify host genes affecting susceptibility to the natural insect pathogen *Metarhizium anisopliae* (Ma549). Overall, 241 (9.22%) mutant lines had altered resistance to Ma549. Life spans ranged from 3.0 to 6.2 days, with females being more susceptible than males in all lines. Speed of kill correlated with within-host growth and onset of sporulation, but total spore production is decoupled from host genotypes. Results showed that mutations affected the ability of *Drosophila* to restrain rather than tolerate infections and suggested trade-offs between antifungal and antibacterial genes affecting cuticle and gut structural barriers. Approximately, 13% of mutations where in genes previously associated with host pathogen interactions. These encoded fast-acting immune responses including coagulation, phagocytosis, encapsulation and melanization but not the slow-response induction of anti-fungal peptides. The non-immune genes impact a wide variety of biological functions, including behavioral traits. Many have human orthologs already implicated in human disorders; while others were mutations in protein and non-protein coding genes for which disease resistance was the first biological annotation.

The fruit fly *Drosophila melanogaster* has been the model of choice to develop ideas about innate immunity and host–pathogen interactions^1^,^2^, but much of what we know has been deciphered using opportunistic human pathogens stabbed or injected into immunocompromised flies. These studies model septic injuries but by bypassing the initial steps of cuticular penetration they may not be appropriate models to study commonly occurring insect pathogens^3^. However, they have shown that *Drosophila* activates a wide range of inducible reactions when microbes enter the hemocoel^1^. The fast-acting responses are largely mediated by hemocytes circulating in the hemolymph and include the coagulation or melanization of foreign objects, phagocytosis of microbes and cellular encapsulation of parasites^1^. The slow response is induced over the course of several hours following a systemic infection, and is tailored to combat specific pathogen classes. The anti-fungal response is largely mediated by the evolutionarily conserved *Toll* pathway^1^, and leads to induction of antifungal peptides, mainly Drosomycin (Drs) and Metchnikowin, into the hemolymph.

Fungi cause the majority of insect disease^4^, and include species such as *Metarhizium anisopliae* that are naturally pathogenic to *Drosophila*. Aside from playing a crucial role in natural ecosystems, *Metarhizium* spp. are being developed as alternatives to chemical insecticides, and as a model for understanding how fungi infect insects^5^. These endeavors could benefit greatly from using the *Drosophila* model system to investigate the interplay between host components and fungal strategies to circumvent these components. However, other than the well-characterized activation of the Toll pathway^6^, little is known of the genetic architecture of *Drosophila’s* interactions with naturally occurring fungal pathogens.

Here we report the results of a screen for mutations affecting *Drosophila*’s ability to resist infection by *M. anisopliae* ARSEF549 (Ma549), utilizing *Minos*-element insertion lines that were constructed in isogenic backgrounds. Unlike viruses and bacteria that normally infect through the oral route, *M. anisopliae* breaches the cuticle reaching directly into the hemocoel using a combination of mechanical pressure and an array of cuticle-degrading enzymes^5^. This renders *M. anisopliae* much more amenable to screening than opportunistic human fungal pathogens that have to be injected into the hemocoel to cause infection, and allowed us to study the whole suite of host defenses that the fly is able to mount. By focusing on pathogen life history traits and fly survival rather than just monitoring antimicrobial peptide (AMP) transcription, as many studies do^1^, we looked at how host genotypes affect pathogen fitness. Adaptation of pathogens to their hosts depends critically on factors affecting pathogen reproductive rate, but the extent to which varying host genotype might affect the evolution of pathogen life history is unclear^7^. We also examined the effects of host genotype on variation in critical stages of a pathogens life history, and at the interconnection of defense with other aspects of host physiology that can set the stage for trade-offs between immunity and other costly life-history traits^8^,^9^. This study establishes a foundation for understanding the genes imparting *Drosophila*’s resistance to a natural fungal pathogen, and will allow the identification of gene networks that may be specific to *M. anisopliae* infection, to insect pathogenic fungi in general, or to a variety of opportunistic mammalian pathogens.

## Results

To gain insight regarding the genes and pathways required for normal defense against fungi, we conducted a forward genetic screen in the *Drosophila Minos* insertion mutant collection (2613 single, homozygous lines representing ~15.8% coverage of the fly genome, based on 14029 protein coding genes in *Drosophila*^10^).

**Figure 1:**
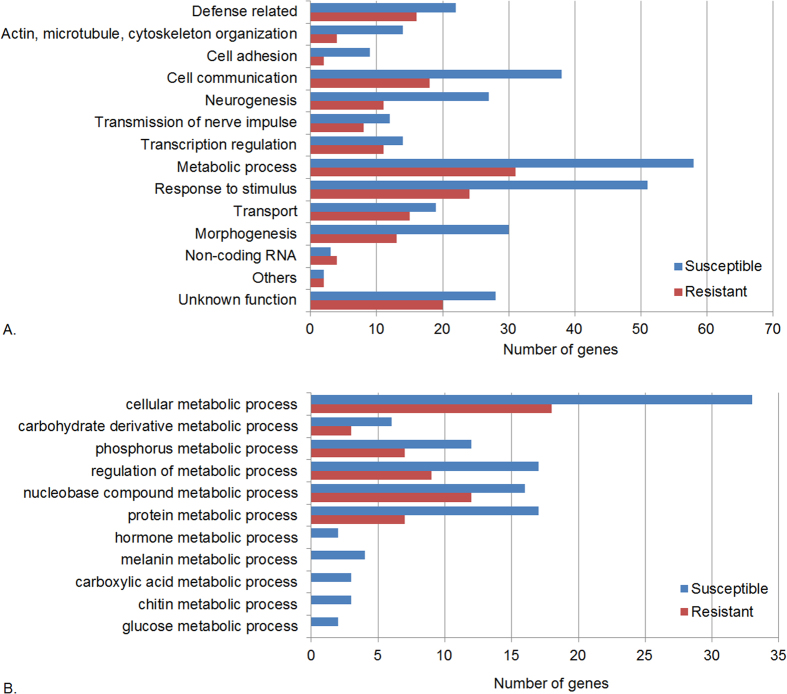
Classification of *Drosophila* genes that change susceptibility to *Metarhizium* infection. (**A**) Genes with mutations increasing (susceptible, blue bar) or decreasing (resistant, red bar) susceptibility to Ma549 infection were classified into categories based on gene ontology and published references. Overall, 147 genes were identified from susceptible lines and 94 genes were identified from resistant lines. Details of genes are in Supplementary Table S1. (**B**) The percentage of genes in the subcategory “metabolic process” was calculated based on 58 genes in susceptible lines and 31 genes in resistant lines. The x-axis indicates the number of genes in each category.

Age-matched flies from each mutant line were infected topically with spores of Ma549, and survival was monitored. A total of 91 mutant lines demonstrated enhanced resistance, while 150 demonstrated lowered resistance compared to wild-type flies, and these were defined as being “resistant” or “susceptible”, respectively (Fig. 1 and Supplementary Table S1). The average LT_50_ was 4.4 days with a range of 3.0 to 6.2 days. Overall, 241 mutant lines (9.22% of the lines we screened) had altered resistance to Ma549. This indicates a large mutational target for disease resistance, consistent with pleiotropy and many biological processes affecting this trait^11^. The asymmetrical distribution of mutational affects indicates more mutations decrease rather than increase disease resistance as expected for a component of fitness.

These 241 lines included 180 with insertions in the coding regions of genes and 19 lines with insertions in upstream promoter regions. Three lines have insertions 2-5kb upstream of a gene and five lines have insertions within 2 kb downstream of the 3’UTR of a gene. These genes are listed in Supplementary Table S1 as they are the only genes in the vicinity of the inserts and as long-distance effects are not uncommon most parsimoniously the inserts affect these genes. Overall, 200 inserts were in or adjacent to protein coding genes (two lines had inserts in the *Connectin* gene), and seven were in long non-protein coding genes, 22.4% of which, including six of the non-protein coding genes, had unknown functions. The *Drosophila* genome has a few hundred non-coding RNAs but they are poorly characterized^12^, so a proposed role for long non coding RNAs in fungal disease resistance is a novel one. The seventh non-coding RNA, *hsr-omega*, has functions affecting protein synthesis and oogenesis. A total of 17 inserts were in regions with no annotated genes within 5 kb of the *Minos* insertion site and either have long-range effects on the neighboring gene(s) or affect an un-annotated gene in the more immediate vicinity. Thirteen lines have inserts located in overlapping genes and four additional lines had inserts in an intergenic region <2kb from two flanking genes, and could affect either or both genes.

### Correlation between traits

To assess the correlation between disease resistance and response to food deprivation we examined times of death of flies maintained on agar (Supplementary Fig. S1). The correlation between disease resistance and starvation longevity (r = 0.02371, p = 0.7181), was not significantly different than zero, indicating that the *Minos* inserts did not decrease general robustness of susceptible flies or increase robustness of resistant flies. Climbing ability is another physiological index commonly applied to flies, and we assessed the correlation between time of death and climbing ability for a subset of 54 randomly selected mutant lines (Supplementary Fig. S1 and Table S2). There was no linear relationship between disease resistance and climbing ability, so the effects are not associated.

**Figure 2:**
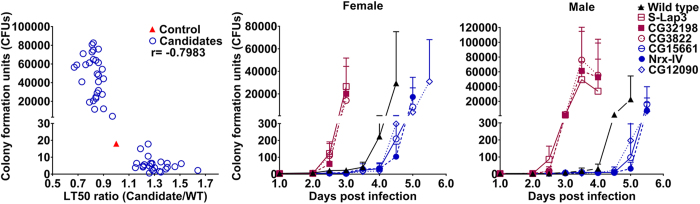
Correlation between traits. (**A**) Negative correlation between fungal growth (colony forming units, CFUs) and male host life span (LT_50_ ratio, candidates vs. wild type) at 3.5 days post infection. CFUs were averaged from 5 individual flies per fly line (experiment repeated twice). (**B**) Time course of CFU production in female or male flies. Three susceptible lines (red) and three resistant lines (blue) are shown as representative examples. The genes affected in these lines are labeled on the right: *S-Lap3* (aminopeptidase), *CG32198* (unknown gene related to defense response to bacterium), and *CG3822* (glutamate receptor) are genes affected in susceptible lines; *CG15661* (glucuronosyltransferase), *Nrx-IV* (transmembrane signaling receptor) and *CG12090* (GTPase activator) are genes affected in resistant lines.

**Figure 3:**
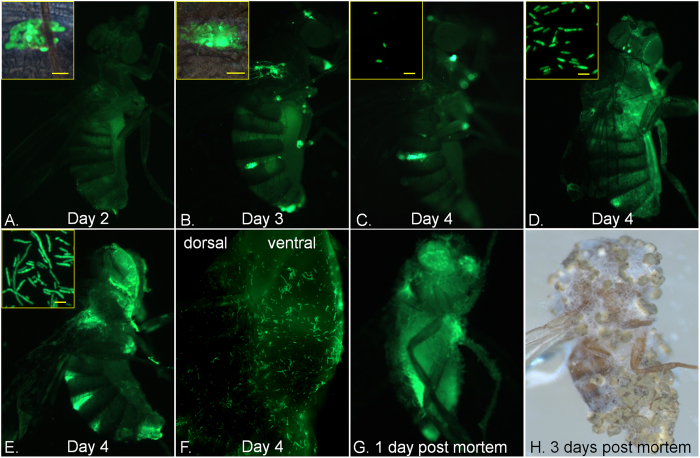
Growth of *Metarhizium* in flies. Wild type flies (*y*^*1*^*w*^*67c23*^), two (**A**), three (**B**), and four days (**C**–**F**) post infection with GFP-Ma549. Fungal growth on abdominal intersegmental membranes (shown in insets, **A**–**B**). At day 4 variable numbers of GFP-Ma549 blastospores (**C** and **D**) and short hyphal lengths (**D** and **E**) are found in in hemolymph samples (shown in insets), and are visible from outside the infected insect’s ventral abdomen (**F**). Flies one-day post mortem (**G**) showing cadavers filled with fluorescent hyphae. Fly three days post mortem showing sporulation on cadaver surface (**H**). Bar in image B represents 50 μm; bars in other images are 10 μm.

To understand the impact of host fitness on fungal fitness we looked at correlations between four key life history traits at different steps of the infection process; within-host growth (fungal load, measured as CFU’s), host life span (LT_50_ values), latent period (the lag time between inoculation and sporulation), and sporulation capacity (the total number of spores per *Drosophila* cadaver). Spore production is a measure of pathogen transmission potential and therefore pathogen fitness^7^. We found a strong negative correlation between host life span and within-host growth 3.5 days post infection (Fig. 2A. r = −0.7983, p < 0.001), i.e., 63.7% (r^2^) of the variation in life span is explained by variation in fungal load at this time point. CFU’s per insect ranged from <5 in the most resistant flies to over 8,000 in susceptible flies, showing that susceptible host genotypes were much less able to restrain fungal growth. A time course of CFU counts confirmed that wild type (LT50 4.3 days) and resistant flies delayed fungal growth compared to susceptible flies (Fig. 2B). CFU’s appeared 2.5 days post infection in female wild type flies but remained <20 per fly until 3.5 days. In all lines, fungal loads sharply increased in the day preceding death, with proliferation occurring 2.5–3 days and 4.5–5 days post-infection in susceptible and resistant flies, respectively. We also used a Ma549 transformant expressing GFP to track infections in whole insects and isolated hemolymph samples (Fig. 3). Fluorescence showed localized fungal infections by day 2 at intersegmental sites that were much larger by day 3 coincident with blastospores (yeast-like budded cells) appearing in the hemolymph. However, massive proliferation of blastospores and subsequently short hyphal lengths only occurred 4 to 4.5 days post-infection in wild type flies, consistent with CFU counts and demonstrating a time lag between penetration and proliferation that did not occur in susceptible lines. Ma549 expressing GFP is sufficiently bright as to be clearly visible from outside the infected insect’s abdomen, which confirmed that blastospores and hyphal bodies accumulated in the body cavity in the day preceding death, and flies continued to fill with fungal hyphae post-mortem leading up to sporulation (Fig. 3G,H).

**Figure 4:**
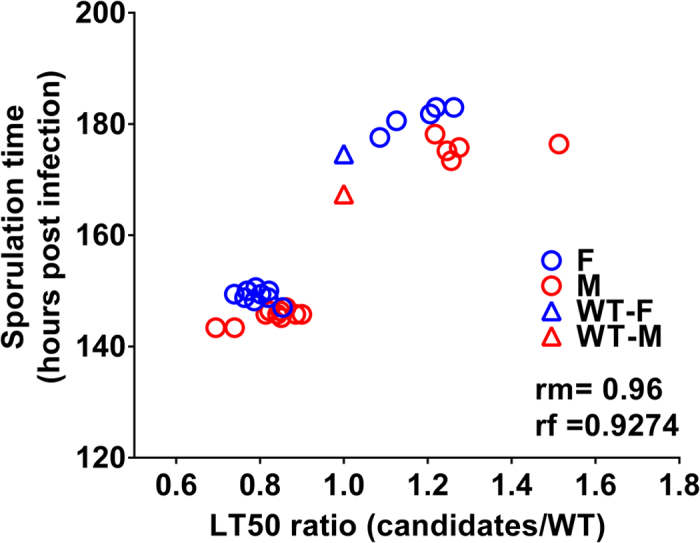
Positive correlation between latent period (time of sporulation) and host life span (LT_50_ ratio). Sporulation time (hours post infection) was averaged for 10 flies per sex per fly line. The LT_50_ ratio was obtained by dividing the LT_50_ of each candidate line with the LT_50_ of wild type flies. Correlation values for males (rm) and females (rf) are shown. Blue: female (F); red: male (M); WT: wild type.

Host genotype impacted the onset of Ma549 sporulation which strongly correlated with life span (Fig. 4); correlation values for males and females were 0.96, and 0.9274, respectively (p < 0.001). That >92% of the variation in latent period is explained by life span is due to sporulation commencing on cadavers within 60 hours post-mortem. However, fewer CFU’s 3.5 days post-infection, and longer host life span or latent period did not correspond to significantly less spore production. Indeed, host genotype had no significant effect on spore production (mean spore production per cadaver per line = 6.1 × 10^6^ for males and 9.8 × 10^6^ for females, all p value ≥ 0.05).

To identify sexual dimorphism, we measured disease resistance separately for males and females in the 54 randomly selected lines (Supplementary S2 Table). We observed significant sex differences, with wild type (control) females and females from all but one of the mutant lines being significantly (p < 0.01) more susceptible than males (average female LT_50_ was 1.08 days less than males). Females disrupted in *CG3940* died faster than males but the difference fell short of significance (p = 0.06046). We found no significant differences between virgin and mated wild type females and between virgin and mated wild type males in susceptibility to Ma549.

### Gene ontology analysis

Gene ontology (GO) analysis revealed diverse categories of genes that confer increased susceptibility or resistance to Ma549 (Fig. 1A). Most (74.7%) of the *Drosophila* genes that affect susceptibility to *Metarhizium* have human counterparts. Overall, 54 lines (22.4%) with altered susceptibility have inserts in genes with no known function in *Drosophila*. Nine of these genes are evolutionary conserved and have human counterparts with no phenotype found, i.e., altered susceptibility to *Metarhizium* is the first phenotype described for them.

**Figure 5:**
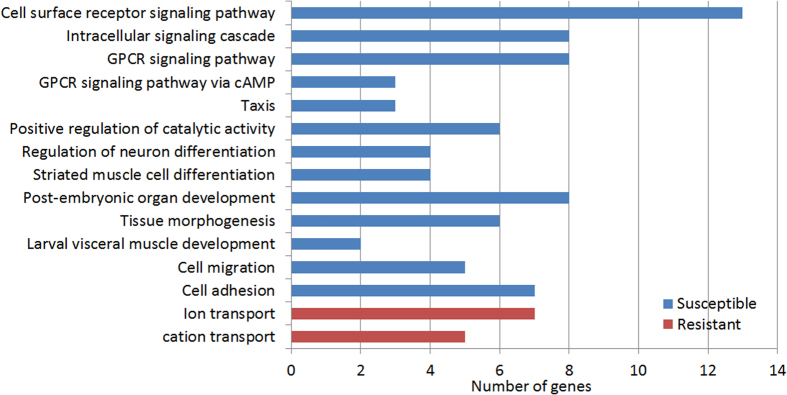
Enrichment of biological function categories of *Drosophila* candidate genes associated with infection sensitivity. Gene ontology categories contain overlapping genes within susceptible or resistant candidates. The x-axis indicates the number of genes in each category.

There is a trend towards differential representation of some gene ontology classes between the resistant and susceptible lines (Fig. 1A). For example, approximately 30% of the susceptible lines are mutated in genes affecting cellular communication, but only 19% of genes in resistant lines fell into this category. Compared to resistant lines, susceptible lines also include a higher percentage of genes affecting metabolic processing, morphogenesis, response to stimulus, neurogenesis, cell adhesion, and cytoskeletal organization. In the subcategory “metabolic process”, 12 mutations (20.7%) in susceptible lines affect processing of hormones, melanin production, glucose, chitin, and carboxylic acid metabolism; no mutations in resistant lines affected these processes (Fig. 1B). GO enrichment analysis confirmed that susceptible lines are significantly enriched in mutations affecting cell adhesion and cellular communication (primarily cell surface receptors and associated signal transduction pathways), as well as organ and tissue development and morphogenesis, and positive regulation of metabolism and cell motility (Fig. 5).

### Immune-related genes altering susceptibility to Ma549

**Figure 6:**
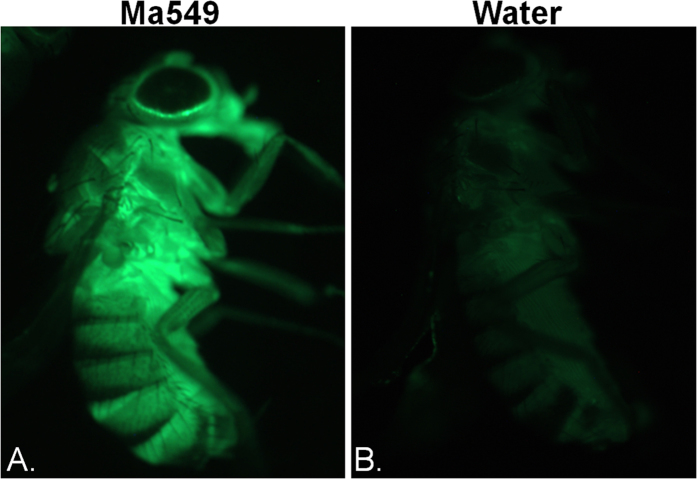
Fluorescent microscope images of Drs-GFP expression. *Drs*-GFP fly infected with Ma549 (**A**), compared with uninfected control fly (vortexed in water) (**B**), showing that systemic infection results in a completely labelled fly. *Drs*-GFP is a reporter system for activation of the Toll immune pathway and shows a strong, albeit unsuccessful immune response to Ma549.

A total of 31 (17 susceptible, 14 resistant) of our 241 candidate genes (13% of total), overlapped with candidate innate immune response genes identified in previous screens using viruses^13^, bacteria^8^,^14^,^15^,^16^,^17^,^18^,^19^ , *Candida albicans*^20^, a protozoan parasite^21^, and the eggs of a parasitoid wasp^22^ (Supplementary Table S1). The Toll and Imd pathogen-recognition pathways are well represented in most of these previous screens. Toll-dependent *Drosomycin* (*Drs*) expression is up-regulated by *Metarhizium*-infected flies^6^, but surprisingly inserts disrupting *pipe* (stock numbers 24732 and 29054) and *spheroide* (29227), involved in activating the Toll pathway, and core Toll signaling pathway components *Pellino* (26071), Gprk2 (26097) and *ush* (23467), did not affect susceptibility to Ma549 although they are known to be involved in production of Drosomycin^23^. We used expression of *Drs-*GFP, a classical read-out of activation of the Toll pathway^24^ to confirm Drs-GFP was induced by infection with Ma549 (Fig. 6). *Drs*-GFP flies disrupted in the critical Toll pathway gene *dif* show no GFP fluorescence in response to Ma549, but nor were they more susceptible to Ma549 (Supplementary Table S3). Real time-PCR confirmed greatly reduced expression of *Drs* in these flies. We conclude that the Toll pathway does not restrain Ma549 even though it is activated by Ma549, and responsible for *Drs* transcription. This contrasts with opportunistic human pathogens which are lethal to Toll mutants but not to wild-type flies^25^.

Although the Toll pathway is ineffective against Ma549, there was substantial evidence that other components of defense provide resistance. A group of susceptible lines were mutated in cuticle biosynthesis genes, and presumably limit cuticle penetration by *Metarhizium* when functioning normally (Supplementary Table S1). An insert in the coagulation (blood clotting) *hemolectin* gene also increased susceptibility. *Drosophila* responds to parasitic wasp eggs in the hemocoel by surrounding the egg with a multicellular capsule^1^. *RhoGEF3* (a Rho-family GTPase) is necessary for encapsulation^22^, and an insert in *RhoGEF3* increased susceptibility of *Drosophila* to Ma549, suggesting commonalities in defense against fungi and larger parasitoids. During encapsulation, plasmatocytes deposit a protein layer onto the parasite derived from their extracellular matrix proteins (ECM)^22^. Disruption of plasmatocyte ECM protein *peroxidasin,* that functions in both phagocytosis and encapsulation^26^, increased susceptibility to Ma549 as did inserts in the transcription factor *caupolican* and a clathrin-binding protein (*likeAP180*) that regulate phagocytosis against *C. albicans*^20^, and *Cryptococcus neoformans*^27^, respectively.

Disrupting an FGF receptor (*heartless*), an endopeptidase (*CG11843*), an ATPase involved in cell motility (*CG14838*), a peroxidase (*CG5873*) and two genes with unknown functions (*CG14331* and *CG32198*) increased susceptibility to *Serratia marcescens*^18^ as well as to Ma549. However, there was also evidence for trade-offs between antibacterial and antifungal genes. The group of 16 Ma549 resistant lines disrupted in putative defense related genes included a chitin synthase active in the gut and gonads, a JAK/STAT signaling protein (controls host defense in the gut), and 6 antibacterial genes that are active in the gut or gonads. Conversely, knocking down the cuticle development gene *Cpr97Eb* and an unknown gene *CG31323* increases resistance to *S. marcescens*^18^, while decreasing resistance to Ma549. Similarly, knocking down *srpk79D* increases resistance to *Listeria monocytogenes*^8^, but decreases resistance to Ma549.

### Non-immunity genes represented in susceptibility lines to Ma549

The gene ontology categories represented in susceptible lines are involved in a broad spectrum of biological functions including basic cellular processes (mitosis, transcription, translation and protein modification), early development, muscle and nervous system development and function, chemosensation and vision, and metabolism; 25.2% of the mutations represent diverse pathways affecting morphogenesis and neurogenesis. Thirteen genes from susceptible lines (and eight genes from resistant lines), have human orthologs with describers that include an aspect of temperament (including some with dysfunctions associated with mood affective disorders, autism, schizophrenia and Alzheimer’s), or cognitive development including learning.

Nutritional factors and circadian rhythms are known to affect *Drosophila* survival to bacteria^28^,^29^. We found only a single candidate gene, the pleiotropic kinase *CkIIalpha,* associated with circadian rhythms in a previous study^30^. Three of the Ma549 susceptibility lines were mutated in octopamine (*Octbeta2R, Oamb*) and dopamine (*Dop1R1*) receptors, which work together in *Drosophila* learning, and form memories for sugar and other foods^31^. Two susceptible lines had insertions in (*dpr4*) or near (*dpr15*) members of the Ig family that regulate taste perception and proboscis extension^32^. Three susceptible lines (*AstA-R1, AstA-R2* and *AstC-R2*), were mutated in the allatostatin signaling pathway that directly regulates food intake by adult *Drosophila*^33^. This enrichment of genes involved in appetite is consistent with nutritional status being a big factor in resistance to fungal pathogens. However, we did not find a simple relationship when we looked at the effects of these feeding behavior genes on starvation stress. *Octbeta2R*, *Oamb* and *Dop1R1* mutated lines were not significantly altered in starvation tolerance; *dpr4* (t = 3.237, p = 0.0119), *dpr15* (t = 2.839, p = 0.0218) and *AstA-R2* (t = 5.620, p = 0.0005) lines were significantly more resistant and *AstA-R1* was significantly less (t = 6.054, p = 0.0003) resistant to starvation.

**Figure 7:**
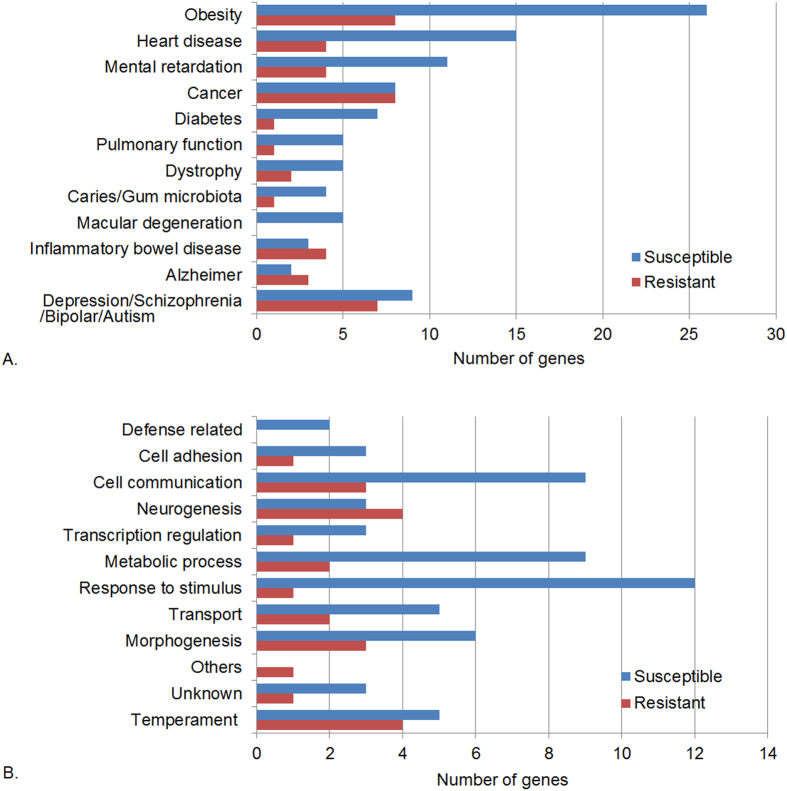
Diseases associated with human orthologs of *Drosophila* candidate genes. **(A**) The number of *Drosophila* genes with human orthologs related to disease. (**B**) *Drosophila* genes with human orthologs related to obesity. *Drosophila* gene categories were the same as Fig. 1. *Drosophila* genes with human orthologs affecting obesity and temperament are listed at the end of the bar chart. The x-axis indicates the number of genes in each category.

Although proboscis extension and allatostatin are specific to arthropods, dopamine and octopamine have similar functions in *Drosophil*a and mammals^31^. Many elements of metabolic homeostasis are conserved between flies and mammals, including genes for fat storage^34^, so we looked for concordant mammalian systems that influence disease and physiology. In total, 53.9% of the genes associated with changes in disease susceptibility in *Drosophila* have human counterparts associated with diseases, with the obesity category being one of those prominently associated with susceptibility (Fig. 7A). *Drosophila* mutants in the obesity category included orthologs to human genes impacting metabolism (including *lnk* involved in insulin signaling), sensory inputs, and immunity (Fig. 7B).

If functions are conserved, mutations in obesity related genes could affect starvation resistance. However, the mutational correlate between starvation tolerance and Ma549 resistance in lines mutated in “obesity category” genes was not significantly different from zero (r = −0.1252, p = 0.4807). Starvation tolerance may depend on metabolic state, but Ma549 resistance in lines affected in basic metabolic processes also did not correlate with starvation resistance (r = −0.09877, p = 0.2771) (Supplementary Fig. S1). Analyzed individually, 59.8% of obesity and metabolic process genes had significant and 31.3% highly significant (p < 0.001) effects on starvation tolerance. However, this was not significantly different than the total Ma549 resistant and susceptible mutant population showing altered starvation tolerance (p = 0.7181).

### Non-immunity genes represented in resistant lines to Ma549

Six behavioral genes were found in Ma549-resistant mutant flies. Of particular note, mutations in *Spn*, *Arrest* and *Px* previously implicated in reduced aggressive behavior in flies^35^, increased resistance to Ma549, suggesting a possible trade-off between hostile social interactions and disease resistance. We also found disrupting two of *Drosophila*’s *Synaptotagamins* (*Syt ß*, *Syt 7*), increased resistance. *Synaptotagamins* are responsible for multiple types of Ca^2+^-induced exocytosis, but their exact biological roles in mammals and *Drosophila* are controversial^36^,^37^. Disrupting the glutamate receptor *CG31760* and the glutamate Ca^2+^ ion channel *CG11155* also increased resistance consistent with Ca^2+^ having an unknown function inhibiting *Drosophila’*s defense against Ma549. Two other genes (*CG13793* and *Neurexin IV*), associated with exocytosis and neurotransmitter transport were disrupted in resistant lines as was *Still life*, a Rho GTPase regulator of the neural cytoskeleton.

As with susceptible *Drosophila* lines, resistant lines were disrupted in genes with pleiotropic effects on other complex traits such as morphogenesis and response to stimuli. Not surprisingly, resistant lines were disrupted in fewer (three) positive regulators of processes than susceptible lines (eleven). Three resistant lines were disrupted in negative regulators of multicellular growth (*Ac76E*), transcription (*pgc*) and translation (*Arrest*). As previously mentioned *Arrest* is also implicated in moderating aggression illustrating the pleiotropic nature of many genes implicated in disease resistance.

## Discussion

Ma549 life history stages show apparent density-dependent effects; greater growth within the host was correlated with shorter life spans and earlier onset of reproduction. The most commonly accepted model assumes that within-host growth is a critical parameter in the evolution of virulence, but that high virulence is traded-off against increased host mortality cutting short the infectious period for transmission between hosts^7^,^38^. In contrast, variation in within-host Ma549 growth between *Drosophila* lines and their susceptibility to Ma549 does not affect total spore production on individual flies because Ma549 sporulates only after host death. Instead, trade-offs effect latent periods which are longer on resistant hosts, so a *Metarhizium* strain that evolves to kill these as quickly as susceptible lines should have higher lifetime fitness. However, transgenic *Metarhizium* strains that killed very quickly came at a cost of decreased spore production^39^,^40^, which will limit the apparent benefit of a very short latent period and thereby constrain the evolution of virulence. These results suggest that differing host pathogen interactions will produce widely varied costs that impact where trade-offs occur between life history traits but will not eliminate them.

Unlike some other *Metarhizium* strains, Ma549 does not use toxins and kills principally by invasive growth and depleting the host of nutrients^41^. We predict this pathogen strategy would likely induce selection on the host to reduce fungal infectivity and growth as these are the traits most strongly affected by host genotype. Sporulation capacity is a measure of host resistance to plant pathogens^7^, but that would not apply to Ma549 and *Drosophila*. Fly lines that succumbed quickly to Ma549 all had higher fungal loads than wild type flies 3.5 days post-infection, suggesting that the principal defect in these flies is that they are less able to restrain *Metarhizium* growth. In contrast, many *Drosophila* mutants succumb to bacterial infections because of defects in tolerance rather than resistance^8^,^42^. This distinction may be because it is more difficult to evolve tolerance traits to a filamentous fungal pathogen that unlike bacteria actively penetrates and colonizes infected tissues. Plausibly, mutations in growth factor signaling pathways such as *pvf1,* involved in wound repair^43^, or structural components such as *dystrophin* increase susceptibility because these genes are normally modulators of both tolerance and resistance. This could occur if reduced damage repair in the vicinity of invasive hyphae facilitates rapid fungal growth. However, none of the resistant lines appeared to tolerate Ma549 better than the wild type because their delayed mortality coincided with delayed rapid proliferation of Ma549.

The first line of defense that prevents microbial invasion into the hemocoel is structural, and comprises the external cuticle (for fungi), the gut peritrophic matrix (for bacteria), and the tracheal lining. Mutations in some genes that affect the gut matrix caused Ma549 infected flies to live longer implying trade-offs between resistance to fungal diseases and increased susceptibility to gut pathogenic bacteria. Possibly, knocking out antibacterial defenses in the gut allows resources to be diverted to anti-fungal defenses in the cuticle and hemolymph. There are alternative possibilities such as antagonism between Ma549 and commensal bacteria if the latter flourish more in some *Drosophila* lines. However, the concept of trade-offs is central to many evolutionary hypotheses for limited lifespan and optimal allocation of resources^44^. It is likely that future studies in this direction will focus on the interactions between host defenses and global metabolism of *Drosophila* and in particular determine how nutrients and energy are distributed among organs during different phases of bacterial and fungal infections^41^.

Modeling fungal or bacterial infections in *Drosophila* typically involves injecting the pathogen of interest into female flies because of their larger size and relative resistance to injection injury when compared with male flies^45^. By only bio-assaying females these studies may have missed sex differences as we found females in all lines were more susceptible to Ma549. This contrasts sharply with screens for other multifactorial phenotypes such as aggression and oxidative stress resistance where many mutations had different effects on males and females^46^. Potentially, the increased susceptibility of females may be due to the energy demands of oogenesis^9^, but we found age-matched virgin and mated females were similarly susceptible to Ma549, whereas mating reduces the ability of female *Drosophila* to defend against bacteria^47^. Presumably, the immune processes suppressed in mated females do not restrain Ma549 and therefore do not impact the progress of a natural infection.

Even when fungal pathogens are experimentally introduced directly into the fly hemolymph, wild-type flies are still capable of eliminating infection because they activate a wide range of immune mechanisms^1^,^2^. Because wild-type *Drosophila* are resistant to most pathogenic fungi and bacteria^48^, Toll deficient flies are frequently employed to model infections caused by medically important fungi. The Toll pathway is therefore regarded as having a critical role in immunity against fungi^1^,^2^, but at least for dif-regulated components this does not apply to the natural pathogen Ma549. Evolving resistance against antimicrobial peptides was probably a pre-requisite for *Metarhizium* spp. to function as professional entomopathogens. Tolerance to products of the Toll pathway, e.g., drosomycin, will prevent clearance of Ma549 from the hemocoel, which likely dooms the fly. Nevertheless, the degree of functioning of the cellular immune response against Ma549 was somewhat surprising. *Metarhizium* blastospores can evade hemocytes by producing a hydrophilic collagen (Mcl1) coat^49^. Furthermore, conidia can be internalized and grow within arthropod phagocytic cells, which may facilitate dispersal through the insect body^50^. Nevertheless, we show that disrupting many genes that influence cellular immunity increases susceptibility. The potential immune function of clotting in response to Ma549 infection was particularly unexpected. Clots composed of melanized hemolectin fibers are rapidly generated at the site of an injury and act to trap microbes. It is an integral part of the insect immune response to bacteria and nematodes^51^,^52^, but we are not aware of any similar reports in response to fungi. Infection by *Metarhizium* often produces a melanization reaction in the cuticle at the site of entry^53^, and the involvement of hemolectin suggests that penetrant fungal hyphae are recognized as septic wounding or that clotting can occur in the absence of significant injury. Melanization increases resistance to *Metarhizium* spp., mainly due to toxic effects from L-DOPA oxidation products^53^,^54^.

The approximately 87% of genes effecting resistance to Ma549 that were not previously implicated in interactions with pathogens explore many different aspects of host defense. Further analysis will be required to formally prove the involvement of these genes in disease resistance, including studying multiple mutant alleles, assessment of temporal and spatial expression patterns and evaluating disease resistance and associated traits in flies in which the genes are over- or under expressed. However, the repeated implication of genes effecting response to stimuli, nervous system development, synapse biogenesis and neuron functioning, temperament and cognition, suggests that some or all of the functions of these processes mediate disease resistance. They imply that disease resistance is linked to complex behaviors perhaps by affecting nutrient uptake or social interactions in a manner which alters disease resistance. *Drosophila* mutants in the obesity category included orthologs to human genes impacting sensory inputs and immunity, and nine have human orthologs that also influence temperament (Fig. 7B), providing a plausible link between behavioral or cognitive traits and disease resistance. Aggression is regulated in part by obesity-linked genes through octopamine signaling^55^, indicating that these genes will provide an entree to analyzing interacting genes linking complex traits such as nutritional factors and disease resistance.

Overall, 9.22% of the mutant lines we screened had altered disease resistance and approximately half of these had significant effects on starvation resistance. Failure to find a simple association between disease and starvation resistance might be expected when networks of pleiotropic genes regulate complex traits, and it is consistent with an earlier screen of P-element lines showing a similar very large mutational target for starvation resistance^56^. Clearly being more or less tolerant to starvation does not by itself alter resistance to Ma549. However, 58 mutations affecting cellular processes and metabolism increase susceptibility (39.5% of all susceptibility genes), and it is plausible that dysfunction in these processes could specifically reduce expenditure of energy on immune responses. Overall, 53.9% of the *Drosophila* genes associated with susceptibility or resistance to Ma549 have been implicated in human diseases or disorders, suggesting that some of the processes that are important in flies are also relevant to mammalian host defense^1^,^2^.

## Material and Methods

### *Drosophila* and fungal stocks

The Drs-GFP, *Dif*^1^ and isogenic control line for *Dif*^*1*^ have been previously described^24^,^57^. We obtained all available *Minos* insertion lines (Mi{ET1}) (listed in Supplementary Table S4) from the Bloomington *Drosophila* Stock Center and these were reared on Cornmeal-molasses-yeast-agar medium with Tegosept and Propionic acid (Genesee Scientific) at 24 ± 3 °C. The single insertion site in each line has been mapped and verified by resequencing of flanking DNA^58^. Two isogenic backgrounds (*w*^*1118*^stock number 5905; *y*^*1*^*w*^*67c23*^ stock number 6599) were employed as controls.

*M. anisopliae* (ARSEF 549) was obtained from the USDA Entomopathogenic Fungus Collection (Ithaca, N.Y). Fungal cultures were moved from −80 degree stock tubes 10 days before each bioassay and grown on potato dextrose agar at 27 °C. Plasmid construction and transformation for GFP fluorescent Ma549 strains was described previously^59^.

### Fungal infection bioassay and screening procedure

During bioassay, flies were maintained at 27 °C, ~85% humidity, on food made without Tegosept and propionic acid. We bioassayed ~30 flies (2–4 days old) per mutant line with conidia from 10 day old Ma549 plates (see Supplementary methods for details). For the pilot screen, LT_50_ values from individual lines were compared with the mean LT_50_ of the experimental cohort treated the same day. Each cohort ranged from 100 to 200 lines. A fly line with an LT_50_ value ±5% different than the average LT_50_ was retested with a new cohort. Out of 2,613 lines, 866 were identified as putative candidates. In the secondary screen, putative candidate lines where compared with their control isogenic background line (3 replicates, experiment repeated at least twice for each candidate), and the LT_50_ values were analyzed using Welch’s T-test. The survival data from candidates were also compared to controls each day post infection using a Log-rank (Mantel-Cox) test (SPSS). Fly lines with significantly lower survival than the wild type (p ≤ 0.05) were identified as susceptible lines and lines with significantly longer survival (p ≤ 0.05) than wild type were identified as resistant lines.

### Gene ontology and expression levels

Detailed information on *Minos* element insertion locations, genes affected by insertions, gene ontology, and gene expression levels were obtained from Flybase (http://flybase.org), Blast2Go Tool (https://blast2go.com), and FlyAtlas (http://flyatlas.org). In addition, we manually looked for genes that may be affected by the *Minos* element if the insertion site is within 2 kb from a gene. Gene ontology enrichment analysis was conducted using the DAVID program^60^.

### Human orthologs and disease/traits

We used the DRSC platform (http://www.flyrnai.org) to identify human orthologs and disease traits, and analyze alignments and scores. The DIOPT-DIST search tool in DRSC includes information from the NCBI OMIM database and genome-wide association studies. A low score (≤2) was excluded unless it was the only match score.

### Starvation assay

A group of ~150 flies (2–4 days old) per mutant line were sub-divided into five fly vials each containing 5 ml of 1% agar and maintained at 27 °C, ~85% humidity. Dead flies were recorded every 8 hours for 72 hours. LT_50_ values were calculated using SPSS 22.0 and compared with the average LT_50_ from the same isogenic background.

### Sex-dependent infection assay, climbing ability, fungal growth and sporulation capacity

We randomly selected 29 susceptible and 25 resistant strains (Supplementary Table S2), to test if the sex of flies influenced disease resistance, and also used this subset of flies to quantify pathogen life history stages and climbing ability. The selected lines all have the *y*^*1*^*w*^*67c23*^ background and so the *y*^*1*^*w*^*67c23*^ line was used as a control. To measure sexual dimorphism, ~25 females or males per line were tested per tube and the infection methods were as described in the fungal infection assay. Each experiment was replicated three times and LT_50_’s were calculated using SPSS 22.0.

To measure fungal growth in the haemolymph, 3.5-days post inoculation, 5 flies per sex were individually homogenized with 45 μl of 0.1% Tween 80. As most females from susceptible lines were dead by 3.5 days, we only analyzed data collected from the males of these lines. For resistant lines, the entire 45 μl homogenate was spread onto selective medium plates. For susceptible lines, 5 μl of a 10-fold dilution of the homogenate was spread onto plates. Colony forming units (CFUs) were counted after 7 days. For latent period and sporulation capacity, ten flies per sex harvested within 12 hours of death, were individually transferred into tubes containing a damp cotton ball and time of appearance of spores (latent period) was recorded. After 20 days, 500 μl of 0.1% Tween 80 was added to each tube, the tubes were vortexed (1 minute), and spore counts per individual fly were made using a hemocytometer. Results are the average of 10 flies per line.

To assess climbing ability, a group of ~25 flies were transferred without anesthesia to a 50-mL conical tube. The flies were tapped to the bottom of the tube as a timer was simultaneously started. Images were taken at three, six and nine seconds and subsequently processed to determine the height of individual flies at each time point. Height and time values were then used to generate the slope of the line. Three replicates per line were performed and compared with the wild type control using Student’s t-test.

Correlations between LT50 survival values and starvation, CFU counts, latent period, sporulation capacity, and climbing ability, were analyzed using GraphPad Prism 6 (GraphPad Software, Inc.).

## Additional Information

**How to cite this article**: Lu, H.-L. *et al.* Identification of *Drosophila* Mutants Affecting Defense to an Entomopathogenic Fungus. *Sci. Rep.* **5**, 12350; doi: 10.1038/srep12350 (2015).

## Supplementary Material

Supplementary Information
