# Supplementary material for: Identification of Drosophila Mutants Affecting Defense to an Entomopathogenic Fungus
Source: Sci Rep. 2015 Jul 23;5:12350. doi: 10.1038/srep12350 (PMC4511952; doi:10.1038/srep12350)
Supplement: Supplementary Information [file srep12350-s1.pdf]

Title: Identification of *Drosophila* Mutants Affecting Defense to an Entomopathogenic Fungus.

Hsiao-Ling Lu, Jonathan Wang, Markus A. Brown, Christopher Euerle, Raymond J St. Leger*

Department of Entomology, University of Maryland, College Park, Maryland, United States of America

Supplementary methods:

Fungal infection bioassay and screening procedure

To prepare inoculum, conidia were suspended in sterile distilled water, vortexed for 2 minutes and filtered through Miracloth (22-25µm) (Andwin Scientific) to remove mycelia. Spore concentrations were determined using a hemocytometer and adjusted with water to make a final concentration of 2.5×10^4 conidia/ml. Flies were vortexed with spore suspensions (20 ml, 2.5×10^4 conidia/ml) for 30 seconds, collected by filtering the suspensions through Miracloth, and transferred into vials containing fresh food. Less than 10% of flies vortexed with water alone (mock-infected), or conidial suspensions died within two days, with no significant differences between lines, so flies succumbing within two days post-infection were deleted from the infection data. Flies were flipped into fresh vials of food every other day. The number of dead flies was recorded twice per day for 7 days and LT_{50} values (lethal time in days at which 50% of the flies died), were calculated using the SPSS 22.0 (IBM Corp.). This method was highly reproducible with a mean LT_{50} value for control flies of 4.682 ± 0.029, N = 126

To determine the inoculum load achieved with a 2.5×10^4 conidia/ml suspension, flies infected one hour previously were individually vortexed in 100µl of 0.05% Tween 80 and the wash was plated onto *Metarhizium* selective medium [Rose Bengal Agar supplemented with Oxytetracycline (80 mg/l), Cycloheximide (250 mg/l), Streptomycin (80 mg/l), and Penicillin (80

mg/l)], for counts of colony forming units. The number of spores per male and female *Drosophila* was estimated to be 198 and 219, respectively (t= 0.3140, p=0.7615, n=10).

Supplementary Figure:

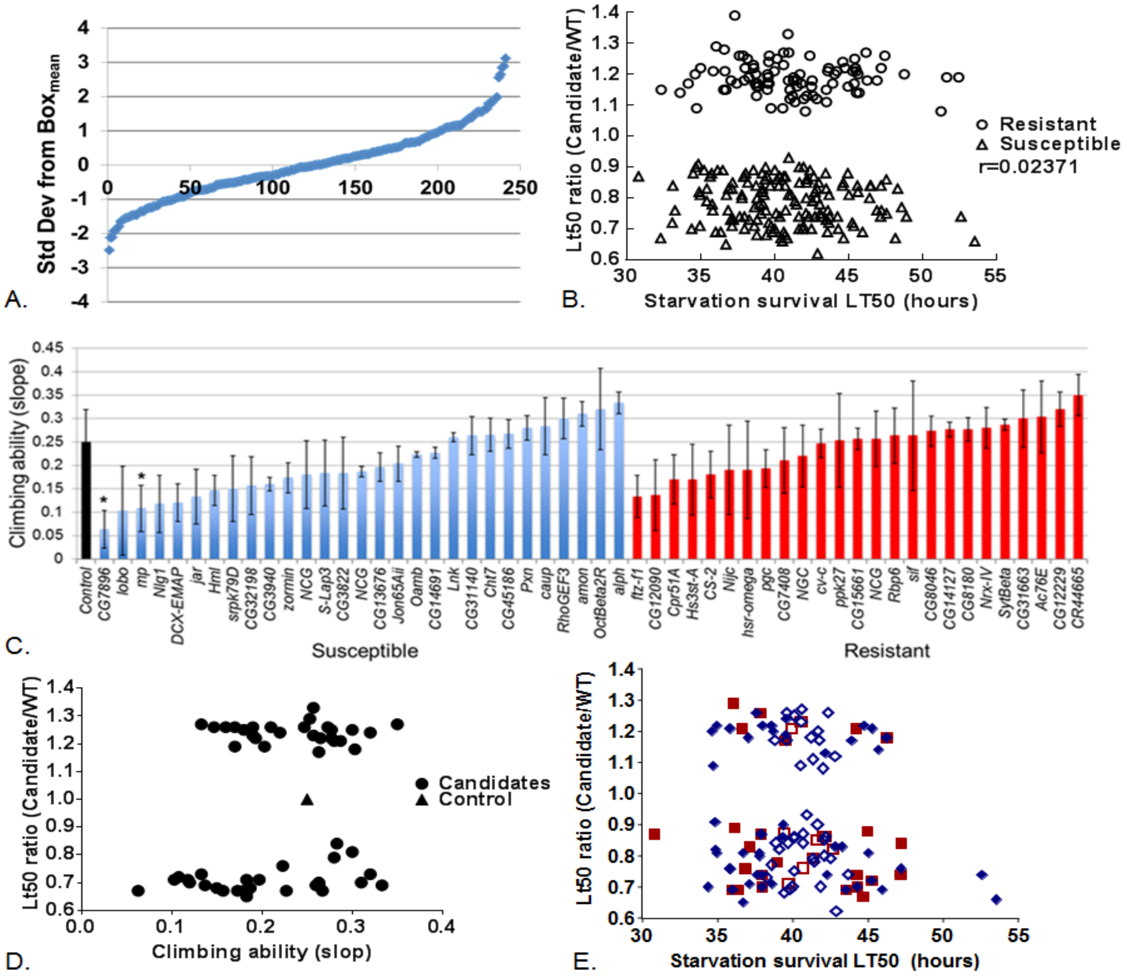

Supplementary Fig. S1. Starvation tolerance and LT_{50} values for candidate lines. A. Total data of starvation tolerance for candidate lines. B. Starvation survival and disease resistance are not correlated (r = 0.02371, p > 0.05). C. Climbing ability of 54 randomly selected lines. The slope of each line was generated from the climbing height and time. Lines significantly different from the wild type control were labeled with an asterisk. D. Climbing ability and LT_{50} values of 54 randomly selected lines shows no linear relationship. E. Starvation survival and LT_{50} values

for genes in the metabolism (blue diamonds) and obesity-related (red squares) categories. Filled in symbols are lines significantly different from the average or control lines of the same background.

Supplementary Tables:

Supplementary Table S1. List of classifications for *Drosophila* genes that change susceptibility to *Metarhizium* infection. The LT50 values from three replicates were averaged and the LT50 ratio of candidate and wild type calculated. Information on the insertion sites was obtained from FlyBase. When an insertion affects more than one gene, the information on each gene is separated with a slash (/). In the defense related gene category, information from references not listed in Flybase's gene ontology are highlighted in gray. For candidate gene expression levels, the tissue categories are nervous (N: head, brain, central nervous system and thoracic-abdominal ganglion), gut (G: crop, midgut, hindgut and Malpighian tubules), gonad (R: spermatheca, ovary, testis and male accessory gland), fat body (F), and carcass (C). The expression scores assigned by FlyAtlas are 0 (no expression), 1 (low expression), 2 (moderate expression), 3 (high expression), and 4 (very high expression).

**List of susceptible lines**									
**Insertion line**	**LT50 ratio (candidate/wild type)**	**Nearest Gene (s)**	**Insert location**	**Gene ontology**		**References**	**Human ortholog(s) (DIOPT scores)**	**Disease/Trait**	**Gene expression scores**
				**Molecular function**	**Biological process**				
**Defense related-physical barrier**									
FBti0113966	88.8%	Cpr49Ad	Exon	Cuticle protein	Cuticle development.		-	-	N0; G0; FB0; R0; C0.

FBti0076263	76.9%	Cpr97Eb	Exon	Cuticle protein	Cuticle development; defense response to bacterium.	RNAi increase resistance to bacterial infection [22].	-	-	N0; G0; FB0; R1; C2.
FBti0075385	86.9%	CG1136	Intron	Cuticle protein	Unknown.		-	-	N1; G2; FB2; R4; C2.
FBti0075299	71.0%	CG13676	5'UTR intron	Chitin binding	Chitin metabolism.		-	-	N0; G1; FB0; R1; C0.
FBti0076229	83.6%	Cht7	5'UTR intron	Chitinase activity	Chitin catabolism.		CHIT1 (7)	Chitotriosidase deficiency.	N0; G0; FB1; R0; C1.
FBti0100466	73.8%	Hemolectin	Intron	Chitin binding	Chitin metabolism; hemolymph coagulation; cell adhesion; wound healing; hemostasis.		OTOG (4) MUC5B (3) VWF (3)	OTOG - Deafness. MUC5B - Pulmonary fibrosis. VWF - Platelet volume.	N2; G1; FB1; R1; C2.
**Defense related- canonical immune system (humoral & cellular)**									
FBti0075291	80.5%	RhoGEF3	Exon	RhoGEF activity	Melanotic encapsulation of foreign target.	[26]	SPATA13 (2) ARHGEF9 (2)	SPATA13 - Airflow obstruction, depression and alcohol dependence, orofacial clefts. ARHGEF9 - Epileptic encephalopathy.	N0; G0; FB0; R0; C0.
FBti0075259	78.6%	Peroxidasin	Intron	Peroxidase activity	Phagocytosis; oxidation-reduction process.	[29]	PXDN (10)	-	N2; G3; FB0; R1; C2.
FBti0076243	70.2%	DCX-EMAP	Intron	Microtubule binding	Melanotic encapsulation of foreign target; locomotory behavior; microtubule-based process; sensory perception of sound.	[26]	EML1 (6) EML2 (6)	EML2 - Subcutaneous adipose tissue.	N1; G0; FB0; R0; C1.
FBti007573	75.5%	Caupolica	Intron	Transcription	Phagocytosis; transcription	[24]; RNAi	IRX6 (4)	IRX6 - Visceral fat.	N2; G1; FB0;

9		n		factor activity	regulation; morphogenesis; defense response to bacterium.	increase susceptible to bacterial infection [22].	IRX5 (4)	IRX5 - Dialysis-related mortality, Hamamy syndrome.	R2; C1.
FBti0114283	89.4%	likeAP180	3'UTR	Clathrin binding	Phagocytosis; synaptic vesicle endocytosis.	RNAi reduce phagocytosis of *C. neoformans* [30].	PICALM (9) SNAP91 (7)	PICALM - Alzheimer's disease, Tourette syndrome, leukemia. SNAP91 - Bipolar disorder.	N4; G1; FB1; R1; C1.
FBti0076392	74.0%	CG10418	3'UTR	Unknown	mRNA splicing; neurogenesis; immune response.	RNAi suppress activation of Dipt-lacZ by bacterial LPS [21].	LSM2 (9)	-	N2; G2; FB2; R2; C2.
FBti0075794	75.9%	kek6	5'UTR intron	Unknown	Lateral inhibition; immune response.	RNAi suppress constitutive activation of Dipt-lacZ [21].	LRTM1 (1) LRRC24 (1) LRFN2 (1)	LRFN2 - Multiple cancers (lung, gastric, squamous cell carcinoma), obesity related traits.	N0; G0; FB0; R0; C0.
**Defense related- non-canonical immune system**									
FBti0100416	82.9%	Heartless	5'UTR intron	Protein tyrosine kinase activity	Morphogenesis; cardiovascular system development; cell proliferation; defense response to bacterium.	[22]	FGFR3 (9) FGFR4 (8) FGFR2 (8) FGFR1 (7)	Several types of cancer (e.g. bladder, prostate, colon, cervical cancers), Craniosynostosis syndromes, mental retardation.	N2; G3; FB1; R1; C1.
FBti0075265	67.8%	srpk79D	3'UTR	Protein serine threonine kinase activity	Protein phosphorylation; synaptic transmission; defense response to bacterium.	Insertion line is more resistant to bacterial infection [8].	SRPK3 (6) SRPK1 (5) SRPK2 (5)	SRPK2 - Pathological myopia.	N2; G1; FB1; R1; C1.
FBti0075658	90.8%	CG11843	Exon	Serine endopeptidase activity	Proteolysis; defense response to bacterium.	RNAi increase susceptible to bacterial infection	-	-	N0; G0; FB0; R0; C1.

						[22] .			
FBti0078252	75.3%	CG14838	Exon	ATPase activity	Microtubule-based movement; defense response to bacterium.	RNAi increase susceptible to bacterial infection [22]	WDR63 (9)	-	N0; G0; FB0; R2; C0.
FBti0075741	83.5%	CG31323	5'UTR intron	Unknown	Defense response to bacterium.	RNAi increase resistance to bacterial infection [22].	-	-	N1; G4; FB0; R1; C0.
FBti0075388	77.9%	CG4329	Exon	Unknown	Sensory perception of sound; regulation of JAK/STAT signaling.	RNAi decrease JAK/STAT signaling in S2 cells [20].	WDR65 (9)	van der Woude syndrome (cleft lip).	N0; G0; FB0; R3; C0.
FBti0075428	80.8%	CG5873/CG14331	Intron/intron	Peroxidase activity/unknown	Oxidation-reduction process; defense response to bacterium/defense response to bacterium.	CG5837: control bacterial producing LLO toxin [17]. CG14331: RNAi increase susceptible to bacterial infection [22].	CG5873 - EPX (2) TPO (2)	EPX - Eosinophil peroxidase deficiency. TPO - Thyroid dyshormonogenesis.	N0/1; G1/0; FB0/0; R1/0; C0/0.
FBti0100458	67.1%	CG32198	1.2kb upstream	Unknown	Defense response to bacterium.	RNAi increase susceptible to bacterial infection [22].	-	-	N0; G0; FB0; R0; C0.
**List of other susceptible lines:**									
FBti0115830	84.8%	CG12507/katanin 80	500 bp upstream/20 bp downstream	Unknown/microtubule binding	Unknown/dorsal appendage formation; microtubule-based process.		kat80 - KATNB1 (8)	-	N0/2; G0/2; FB0/1; R1/2; C0/1.

FBti0075753	81.4%	CG32944	Intron	Protein serine threonine kinase activity	Actin filament organization; protein phosphorylation.		STK32B (7)	Alzheimer's disease, Coronoary heart disease.	N2; G0; FB0; R0; C1.
FBti0075765	73.0%	Jaguar	5'UTR intron	Myosin protein,	Actin filament-based process; embryonic development.		MYO6 (8)	Hypertension, deafness, hypertrophic cardiomyopathy.	N2; G4; FB2; R3; C2.
FBti0078329	89.9%	DAAM	5'UTR intron	Rho GTPase binding	Axonogenesis; filopodium assembly; cuticle pattern formation; actin cytoskeleton organization		DAAM1 (10) DAAM2 (8)	DAAM2 - Pulmonary function.	N3; G3; FB1; R2; C1.
FBti0076261	67.5%	zormin	Intron	Structural constituent of cytoskeleton	Unknown.		NCAM1 (1) NCAM2 (1)	NACM1 - Entorhinal cortical thickness, left ventricular mass. NCAM2 - . Longevity, obesity.	N2; G4; FB1; R2; C2.
FBti0100418	67.4%	CG45186	Intron	Actin binding	Cytoskeleton organization.		-	-	N2; G3; FB1; R4; C2.
FBti0075283	87.8%	Dscam4	Intron	Identical protein binding	Cell adhesion.		DSCAM (7) DSCAML1 (5)	DSCAM - Neutrophil count, non-small cell lung cancer, obesity-related traits. DSCAML1 – Triglycerides.	N1; G0; FB0; R0; C0.
FBti0075286	74.0%	Roughened	Exon	GTPase activity	Regulation of cell shape; cell adhesion.		RAP1A (10)	Osteoporosis.	N3; G4; FB3; R4; C3.
FBti0077628	89.1%	Kirre	Intron	Cell adhesion molecule binding	Cell junction organization; cell adhesion; renal filtration; muscle cell differentiation.		KIRREL3 (6) KIRREL (6)	KIRREL3 - Attention deficit hyperactivity disorder, mental retardation. KIRREL - Obesity-related traits.	N2; G2; FB0; R0; C0.

FBti0114192	83.4%	beaten path Ib	5'UTR intron	Unknown	Heterophilic cell-cell adhesion; axon choice point recognition.		CADM2 (1) CADM1 (1)	CADM2 - Temperament, visceral fat. CADM1 - Cardiovascular disease risk factors, obesity-related traits.	N3; G0; FB0; R0; C0.
FBti0075423	68.7%	Synapsin/Timp	Intron/intron	ATP binding/metalloendopeptidase inhibitor activity	Olfactory learning; neurotransmitter secretion; response to heat/ delamination; cell adhesion; basement membrane organization and morphogenesis.		Synapsin - Syn3 (8) Syn1 (7) Syn2 (5) Timp - TIMP3 (7) TIMP2 (7)	Syn3 - Macular degeneration, height. SYN2 - Epilepsy, platelet counts, type 2 diabetes, schizophrenia. TIMP3 - Macular degeneration, sorsby fundus dystrophy. TIMP2 - Dupuytren's disease, obesity.	N4/3; G0/4; FB0/3; R1/4; C1/3.
FBti0075754	68.7%	Lnk	Intron	Signal transducer activity	Female mating behavior; regulation of insulin receptor signaling pathway.		SH2B1 (6) SH2B3 (4)	SH2B1 - Weight, bowel disease. SH2B3 - Celiac disease, rheumatoid arthritis, coronary heart disease, hypothyroidism, type 1 diabetes.	N2; G2; FB2; R2; C2.
FBti0102016	87.6%	AstA-R1	5'UTR intron	Allatostatin receptor activity	Neuropeptide signaling pathway.		GALR2 (7) KISS1R (3)	KISS1R - Hypogonadotropic hypogonadism.	N2; G0; FB0; R1; C0.
FBti0075352	73.6%	AstA-R2	3'UTR	Allatostatin receptor activity	Neuropeptide signaling pathway.		GALR1 (3)	Obesity-related traits.	N0; G2; FB0; R0; C0.
FBti0100440	81.6%	AstC-R2	5'UTR intron	Allatostatin recept	Neuropeptide signaling pathway.		SSTR2 (6) SSTR5	SSTR5 - Acromegaly, pituitary	N0; G0; FB0; R0; C0.

				or activity			(4) SSTR4 (4)	adenoma. SSTR4 - Obesity-related traits.	
FBti0075743	75.6%	Oamb	5'UTR intron	Octopamine receptor activity	Cellular calcium ion homeostasis; octopamine or tyramine signaling pathway; learning.		ADRA1A (2)	Response to amphetamines.	N2; G0; FB1; R1; C1.
FBti0075293	88.1%	CrzR	Intron	Corazonin receptor activity	Neuropeptide signaling pathway.		GNRHR (2)	Fertile eunuch syndrome, hypogonadotropic hpogonadism.	N1; G1; FB3; R0; C2.
FBti0075290	67.9%	CG42788	5'UTR intron	Unknown	Signal transduction.		FRMPD4 (6)	-	N2; G2; FB2; R1; C2.
FBti0114291	86.7%	TkR99D	Exon	Tachykinin receptor activity	Neuropeptide signaling pathway; olfactory behavior; negative regulation of synaptic transmission.		TACR3 (9) TACR1 (6)	TACR3 - Hypogonadotropic hypogonadism. TACR1 - Brain Connectivity.	N1; G1; FB0; R0; C0.
FBti0075793	85.3%	Dop1R1	3"UTR	Dopamine receptor activity	Learning or memory; cognition; Neuropeptide signaling pathway response to temperature stimulus.		DRD5 (5) DRD1 (5)	DRD5 - Attention deficit hyperactivity disorder, blepharospasm, dystonia. DRD1 - Obesity-related traits.	N2; G0; FB0; R1; C0.
FBti0075261	80.1%	CkIIalpha	5'UTR intron	Casein kinase	Signal transduction; response to stimulus; circadian rhythm; locomotory behavior.		CSNK2A1 (10)	-	N4; G3; FB3; R4; C3.
FBti0100428	89.7%	Dystrophin	Intron	Structural constituent of muscle	Muscle organ development; muscle cell cellular homeostasis; neuromuscular synaptic		DMD (9)	Response to antidepressant treatment, muscular dystrophy, cardiomyopathy dilated.	N2; G3; FB2; R3; C2.

					transmission.				
FBti0075792	70.8%	CG3822	Exon	Glutamate receptor activity	Ion transport; regulation of synaptic activity.		GRIK2 (5) GRIK1 (4)	GRIK2 - Obesity-related traits, obsessive compulsive disorder, mental retardation. GRIK1 - Autism spectrum disorder, bipolar disorder, schizophrenia, breast cancer, major depressive disorder.	N2; G0; FB0; R0; C0.
FBti0075396	70.4%	CG31140	Exon	NAD^+ kinase activity	Protein kinase C-activating GPCR signaling pathway; phosphorylation; lateral inhibition.		DGKQ (7)	Parkinson's disease.	N3; G1; FB0; R1; C0.
FBti0075275	78.9%	Protein Kinase D	5'UTR	Protein serine threonine kinase activity	Intracellular signal transduction; protein phosphorylation.		PRKD3 (8) PRKD1 (8) PRKD2 (7)	PRKD1 - Obesity-related traits. PRKD2 – Leukemia.	N2; G2; FB2; R2; C2.
FBti0076399	75.3%	torso-like	5'UTR intron	Torso binding.	Embryonic pattern specification; torso signaling pathway.		-	-	N1; G3; FB4; R3; C3.
FBti0076397	90.2%	Pvf1	5'UTR intron	Growth factor activity	Cell migration; malpighian tubule morphogenesis; vascular endothelial growth factor receptor signaling pathway.		PDGFA (3)	-	N0; G2; FB1; R3; C1.
FBti0100411	83.0%	sprouty	3'UTR	Unknown	Morphogenesis; muscle cell differentiation; negative regulation of		SPRY2 (7) SPRY4 (6)	SPRY2 - Adiposity, facial clefts, diabetes. SPRY4 -	N2; G2; FB2; R2; C2.

					fibroblast growth factor receptor signaling pathway.			Bowel disease, testicular germ cell cancer, hypogonadotropic hypogonadism.	
FBti0100426	69.0%	Alphabet	3'UTR intron	Protein serine threonine phosphatase activity	Morphogenesis; negative regulation of MAPK cascade; response to UV, and stress.		PPM1A (9)	PPM1A - Allergic rhinitis.	N2; G2; FB2; R2; C2.
FBti0100390	86.9%	dor	5'UTR intron	Receptor binding activity	Autophagy; transcription regulation; ecdysone receptor-mediated signaling pathway.		TP53INP1 (2)	Type 2 diabetes.	N2; G3; FB3; R2; C3.
FBti0075784	73.1%	CG13708	Exon	Unknown	Lateral inhibition.		LRRC49 (7)	-	N0; G1; FB0; R0; C0.
FBti0100407	70.4%	CG16908	Intron	Unknown	Lateral inhibition.		TTI1 (10)	-	N1; G1; FB1; R1; C1.
FBti0076164	74.5%	Ankyrin2/CG32373	Intron/5'UTR intron	structural constituent of cytoskeleton/ calcium ion binding	Microtubule cytoskeleton organization; axon extension; sensory perception of sound/ synaptic target recognition.		Ank2 - ANK2 (6) ANK3 (5) ANK1 (4) CG32373 - FBLN5 (2)	ANK2 - Cancer, cardiac arrhythmia. ANK3 - Autism spectrum disorder, bipolar disorder, schizophrenia, major depressive disorder. ANK1 - Pulmonary function decline, diabetes, Spherocytosis. FBLN5 - Height, Cutis laxa, Macular degeneration.	N-/1; G-/1; FB-/1; R-/1; C-/0.

FBti0075318	73.2%	Octbeta2R	5'UTR intron	Octopamine receptor activity	Synaptic growth at neuromuscular junction; transcription regulation.		HTR4 (3)	Periodontal microbiota, pulmonary function.	N1; G1; FB0; R0; C0.
FBti0075347	71.2%	Neuroligin 1	Intron	Neurexin family protein binding	Synaptic growth at neuromuscular junction.		NLGN4X (3) NLGN1 (3) NLGN3 (3)	NLGN4X - Asperger syndrome, autism, mental retardation. NLGN1 - Major depressive disorder.	N0; G1; FB0; R1; C1.
FBti0077719	76.2%	Neurexin 1	Intron	Apolipoprotein binding	Synaptic transmission; associative learning.		NRXN1 (7) NRXN2 (7) NRXN3 (6)	NRXN1 - Longevity, Autism, Pitt-hopkins-like syndrome, Schizophrenia. NRXN2 - Urate levels. NRXN3 - Obesity, amyotrophic lateral sclerosis.	N3; G1; FB0; R1; C0.
FBti0100420	72.4%	Syntrophin-like 1	Exon	structural constituent of muscle	Synaptic growth at neuromuscular junction.		SNTB1 (8)	Myopia.	N2; G1; FB1; R0; C0.
FBti0076326	84.1%	RhoGAP100F	3'UTR	Rho GTPase activator activity	Axon guidance; signal transduction.		SYDE1 (6)	-	N3; G0; FB0; R1; C0.
FBti0078196	90.1%	cap	Intron	ATP binding	Neurogenesis; morphogenesis; lateral inhibition.		SMC3 (10)	Cornelia de Lange syndrome 3.	N2; G1; FB1; R2; C1;
FBti0076219 FBti0076212	72.9%/ 76.4%	Connectin	Intron /5'UTR intron	Unknown	Homophilic cell adhesion; synaptic target attraction.		RXFP2 (1) SLITRK6 (1)	RXFP2 - Cryptorchidism. SLITRK6 - Bipolar disorder.	N3; G1; FB1; R1; C0.
FBti0075810	72.4%	Multiplexin	Intron	Carbohydrate bindin	Axon guidance; basement membrane organization;		COL18A1 (6) COL15A1 (6)	COL18A1 - Hippocampal atrophy, Knobloch	N4; G1; FB1; R2; C1.

				g	cell adhesion.			syndrome.	
FBti0078411	89.3%	dpr12	5'UTR intron	Unknown	Sensory perception of chemical stimuli; synaptic target recognition.		FGFR4 (1) FGFR3 (1)	Several types of cancer (e.g. bladder, prostate, colon, cervical cancers), Craniosynostosis syndromes, mental retardation.	N1; G0; FB0; R0; C0.
FBti0077623	84.1%	CG1677	5'UTR intron	Metal ion binding	Neurogenesis; mRNA splicing.		ZC3H18 (8)	-	N2; G2; FB2; R3; C2.
FBti0100413	86.7%	Fork head domain 64A	5'UTR	Transcription factor activity	Neurogenesis; regionalization; neuron differentiation; RNA metabolic process; morphogenesis.		FOXL1 (4)	Bone mineral density, obesity-related traits.	N0; G1; FB0; R0; C0.
FBti0114278	85.3%	Lady bird late	Intron	Transcription factor activity	Neurogenesis; cardiovascular system development; RNA metabolic process; morphogenesis.		LBX1 (7)	LBX1 - Scoliosis.	N1; G1; FB0; R2; C1.
FBti0075790	73.6%	Tincar	5'UTR intron	Unknown	Photoreceptor cell development.		-	-	N1; G0; FB0; R1; C0.
FBti0075288	85.9%	TfAP-2	Intron	Transcription factor activity	Post-embryonic organ morphogenesis; adult locomotory behavior; response to stimulus; transcription regulation.		TFAP2A (7) TFAP2D (6) TFAP2B (4)	TFAP2A - Branchiooculofacial syndrome. TFAP2D - Breast size. TFAP2B - Obesity, Char syndrome.	N2; G0; FB0; R0; C0.
FBti0076217	74.2%	CG4576/rec	Exon/intron	Transferase activity/DNA helicase activity	Unknown/DNA replication; meiotic recombination.		rec - MCM8 (7)	Menarche and menopause.	N0/1; G1/1; FB0/1; R0/2; C0/0.

FBti0077792	82.2%	Twin of eyg	Intron	Transcription factor activity	Compound eye development; transcription regulation.		PAX6 (1) PAX4 (1)	PAX6 - Body mass index, corneal dystrophy, cataract, coloboma, foveal hyperplasia, Gillespie syndrome, Keratitis, Peters anomaly. PAX4 - Diabetes.	N1; G1; FB0; R4; C1.
FBti0127529	83.0%	CG42666	5'UTR	Exonuclease activity	Unknown.		REXO1L (4)	-	N0; G1; FB0; R2; C0.
FBti0076332	86.3%	CG4374	5'UTR intron	Nucleic acid binding	Unknown		ZNF827 (1)	Dental caries, Immune response to smallpox, gamma glutamyl transferase level.	N0; G0; FB0; R0; C0.
FBti0076223	69.1%	Jon65Aii	Exon	Serine endopeptidase activity	Proteolysis.		CTRB1 (2) CELA1 (2)	CELA1 - Obesity-related traits.	N0; G4; FB0; R0; C0.
FBti0075469	69.9%	amon	3'UTR	Serine endopeptidase activity	Peptide hormone processing.		PCSK2 (10)	Dialysis-related mortality, menarche, obesity-related traits.	N3; G1; FB0; R0; C0.
FBti0100463	70.6%	furry/CG18179	Intron/30bp upstream	Protein binding/serine endopeptidase activity	Post-embryonic organ morphogenesis/ proteolysis.		Furry - FRYL (9) FRY (7) CG18179 - CTRB1 (2) CTRB2 (2)	-	N2/0; G3/4; FB2/0; R2/0; C2/0.
FBti0075826	82.4%	CG7236	4.2kb upstream	Protein kinase activity	Protein phosphorylation; mitotic cytokinesis.		CDKL1 (6)	Cognitive performance.	N0; G0; FB0; R0; C0.

FBti0076184	71.8%	CG43335	3.5kb upstream	Serine endopeptidase activity	Proteolysis.		GZMB (1) CELA1 (1)	GZMB - Vitiligo. CELA1 - Obesity-related traits.	N0; G0; FB0; R0; C0.
FBti0075320	64.7%	S-Lap3	Exon	Aminopeptidase activity	Proteolysis.		LAP3 (6)	-	N0; G0; FB0; R4; C0.
FBti0100444	76.8%	Elo68alpha	Exon	Fatty acid elongase activity.	Pheromone biosynthetic process; fatty acid elongation.		ELOVL4 (8)	Aging, partial epilepsies, ichthyosis, spastic quadriplegia, mental retardation, macular dystrophy.	N0; G1; FB0; R0; C0.
FBti0075312	75.6%	CG5278	3'UTR	Fatty acid elongase activity.	Fatty acid elongation.		ELOVL4 (2)	Aging, partial epilepsies, ichthyosis, spastic quadriplegia, mental retardation, macular dystrophy.	N0; G1; FB0; R2; C0.
FBti0075752	85.6%	Cyp12a4/Cyp12a5	300bp upstream/70bp downstream	Oxidoreductase activity	Oxidation-reduction process; response to insecticide.		CYP24A1 (5) CYP27A1 (4)	CYP24A1 - Atopic dermatitis, multiple scerosis, hypercalcemia. CYP27A1 - Cerebral cholesterosis.	N2/2; G4/2; FB4/2; R4/2; C3/2.
FBti0101997	86.7%	CG5561/CG31924	5'UTR/3'UTR	Hydrolase activity	Metabolic process.		CG31924 - HDHD1 (7) CG5561 - HDHD1 (4)	-	N0/0; G0/0; FB0/0/0; R3/2; C0/0.
FBti0075785	77.5%	CG10170	Exon	Galactosyltransferase activity.	Metabolic process.		UGT2B15 (2)	Sex hormone-binding globulin levels.	N0; G3; FB1; R0; C0.
FBti007580	69.5%	CG3940	5'UTR	Carbonate	One-carbon metabolic		CA10 (1)	CA10 - Menarche.	N4; G2; FB3;

7				dehydratase activity	process.		CA8 (1) CA4 (1)	CA8 - Cardiac hypertrophy, obesity-related traits, response to amphetamines, cerebellar ataia and mental retardation. CA4 - Retinitis pigmentosa.	R4; C3.
FBti0076215	74.3%	CG5999	1kb upstream	Glucuronosyltransferase activity	Metabolic process.		UGT1A1 (1) UGT2B15 (1) UGT2B17(1) UGT2B7 (1)	UGT1A1 - Bilirubin levels. UGT2B15 - Sex hormone-binding globulin levels. UGT2B17 - Osteoporosis. UGT2B7 - Obesity-related traits.	N0; G2; FB1; R1; C0.
FBti0075748	65.8%	CG31075/CG31076	400bp upstream/ 100bp downstream	NAD activity /Unknown	Pyruvate metabolic process/Unknown.		CG31075 - ALDH2 (5) CG31076 - C10orf11 (5)	ALDH2 - Alcohol consumption, coronary heart disease, esophageal cancer, stroke, upper aero digestive tract cancers. C10orf11 - Pulmonary function, albinism.	N1/0; G2/2; FB1/0; R2/0; C2/0.
FBti0076322	75.8%	CG7059	5'UTR	Phosphoglycerate mutase activity	Glycolysis.		BPGM (3)	Erythrocytosis.	N1; G3; FB0; R3; C1.
FBti0075355	70.4%	CG4483	2kb downstream	Palmitoyltransferase activity	Protein palmitoylation.		ZDHHC6 (8)	-	N0; G0; FB0; R2; C0.
FBti0075282	80.3%	CG34342	100bp upstream	Fatty-acyl-CoA reduct	Unknown.		FAR2 (2)	Platelet volume.	N0; G0; FB0; R0; C0.

				ase activity.					
FBti0114270	92.5%	Dgp-1	Exon	GTPase activity	Unknown.		GTPBP1 (10)	-	N2; G2; FB2; R3; C2.
FBti0100435	69.2%	scp2	900bp upstream	GTPase activity	Unknown.		-	-	N4; G4; FB0; R0; C1.
FBti0075279	61.8%	Ubc87F/CG34383	5'UTR intron /5'UTR intron	Ubiquitin-protein ligase activity/unknown	Unknown/Unknown.		-	-	N0/2; F0/2; FB1/1/0; R4/1; C1/1.
FBti0102022	81.3%	CG15385	Exon	Acid phosphatase activity	Unknown.		ACPL2 (10)	Cognitive performance.	N2; G1; FB2; R2; C1.
FBti0075488	74.2%	dpr4	Intron	Unknown	Sensory perception of chemical stimulus.		CADM1 (1)	Cardiovascular disease risk factors, obesity-related traits.	N2; G0; FB0; R1; C0.
FBti0100520	85.0%	dpr15	2.6kb upstream	Unknown	Sensory perception of chemical stimulus.		CADM2 (2)	Termperament, visceral fat.	N2; G0; FB0; R0; C0.
FBti0075278	81.2%	CG11498	5'UTR intron	Unknown	Cellular response to stress.		-	-	N0; G0; FB0; R1; C0.
FBti0075589	85.6%	NAAT1	Intron	Neurotransmitter transporter activity	Sodium ion and amino acid transport.		SLC6A17 (1) SLC6A14 (1)	Obesity.	N0; G4; FB0; R2; C2.
FBti0075398	82.0%	CG6231	5'UTR intron	Transmembrane transporter activity	Transmembrane transport.		SLC22A14 (1)	-	N1; G2; FB1; R1; C2.
FBti0100425	67.4%	CG14691	Intron	Unknown	Transmembrane transport.		SVA2 (2)	Height.	N0; G1; FB0; R2; C0.
FBti0100437	88.7%	CG14857	Intron	Transmembrane	Transmembrane transport.		SLC22A14 (1)	-	N0; G2; FB0; R0; C0.

				transporter activity					
FBti0075267	83.8%	CG33970	Exon	Transporter activity	Transport.		ABCA13 (1)	Dermatofibrosarcoma protuberans.	N1; G4; FB0; R2; C3.
FBti0076167	70.7%	CG6893	200bp downstream	Transmembrane transporter activity	Unknown.		SLC25A10 (3)	Dental caries.	N0; G0; FB0; R2; C0.
FBti0128239	87.2%	Myo10A	Exon	motor activity	Filopodium assembly; dorsal closure; intracellular protein transport.		MYO15A (7)	Obesity-related traits, deafness.	N1; G2; FB1; R1; C1.
FBti0100453	80.5%	CG42268	Exon	Unknown	Imaginal disc-derived wing morphogenesis.		-	-	N4; G2; FB2; R3; C2.
FBti0075386	80.8%	CG31145	Intron	Protein kinase activity	Imaginal disc-derived wing morphogenesis.		FAM20C (6) FAM20A (5)	FAM20C - Raine syndrome. FAM20A - gingival fibromatosis syndrome, amelogenesis imperfecta..	N3; G3; FB3; R4; C3.
FBti0075301	88.7%	CG43427	Intron	Zinc ion binding.	Imaginal disc-derived wing morphogenesis.		LMO7 (3)	Type 1 diabetes.	N1; G2; FB1; R1; C2.
FBti0100406	85.6%	mRpL43	Intron	Mitochondrial ribosomal protein	Translation.		MRPL43 (9)	-	N2; G2; FB2; R2; C2.
FBti0075771	71.0%	lobo	Exon	Unknown	Sperm storage; sperm motility.		CCDC135 (8)	-	N0; G0; FB0; R3; C0.
FBti0078393	83.7%	Zwilch	Exon	Unknown	Mitotic nuclear division.		ZWILCH (3)	-	N0; G1; FB1; R2; C0;
**Non protein coding gene**									
FBti0075467	80.3%	CR32111	Exon	Unknown	Unknown		-	-	N0; G0; FB0; R0; C0.
FBti010045	72.9%	CR42651	Intron	Unknown	Unknown		-	-	N0; G1; FB0;

1									R0; C0.
FBti0100431	76.1%	CR42836	800bp upstream	Unknown	Unknown		-	-	N0; G0; FB0; R0; C0.
**Unknown**									
FBti0114048	86.9%	CG18420	90bp upstream	Unknown	Unknown		TMPRSS6 (1) CELA1 (1)	TMPRSS6 - Red blood cell traits, iron deficiency anemia. CELA1 - Obesity-related traits.	N0; G0; FB0; R0; C0.
FBti0075796	85.0%	bves	5'UTR intron	Unknown	Unknown		BVES (7)	Height, menarche.	N1; G1; FB1; R2; C1.
FBti0127510	87.6%	CG12880	Exon	Unknown	Unknown		-	-	N1; G1; FB0; R2; C0.
FBti0076232	86.7%	CG13699	3'UTR	Unknown	Unknown		-	-	N0; G0; FB0; R0; C0.
FBti0076189	75.4%	CG14238	Exon	Unknown	Unknown		TMEM26(9)	Diastolic blood pressure.	N0; G0; FB0; R0; C0.
FBti0075321	84.1%	CG14853	5'UTR intron	Unknown	Unknown		ERICH2 (2)	-	N4; G0; FB0; R0; C0.
FBti0075340	83.0%	CG14989	Intron	Unknown	Unknown		-	-	N4; G2; FB0; R1; C1.
FBti0113991	75.6%	CG15252	Intron	Unknown	Unknown		-	-	N0; G0; FB0; R0; C0.
FBti0076210	72.2%	CG15822	Intron	Unknown	Unknown		SESTD1 (6)	Presence of antiphospholipid antibodies.	N0; G2; FB1; R1; C0.
FBti0076333	87.2%	CG15888	Intron	Unknown	Unknown		-	-	N0; G0; FB0; R0; C0.
FBti0100436	66.0%	CG17666	5'UTR	Unknown	Unknown		FOS (1)	Bowel disease, periodontal microbiota.	N0; G0; FB0; R4; C0.
FBti0076346	80.1%	CG32106	Exon	Unknown	Unknown		-	-	N0; G0; FB0; R2; C0.
FBti0075260	83.7%	CG34114	5'UTR intron	Unknown	Unknown		KIRREL3(1) KIRREL (1)	KIRREL3 - Attention deficit hyperactivity disorder and conduct disorder, mental	N1; G0; FB0; R0; C0.

								retardation. KIRREL - Obesity-related traits.	
FBti0078159	87.6%	CG34355	Exon	Unknown	Unknown		-	-	N1; G0; FB0; R0; C0.
FBti0075332	77.7%	CG34391	Intron	Unknown	Unknown		OPCML (2) NTM (2)	OPCML - Breast-ovarian cancer. NTM – Autism, obesity-related traits.	N1; G0; FB0; R1; C0.
FBti0075776	68.9%	CG3984	Exon	Unknown	Unknown		-	-	N0; G1; FB3; R3; C2.
FBti0075357	83.9%	CG42540	Intron	Unknown	Unknown		STOM (5) STOML3 (3) NPHS2 (3)	STOM – Stomatocytosis. STOML3 - Metabolic syndrome. NPHS2 - Nephrotic syndrome type 2	N4; G0; FB1; R0; C0.
FBti0075536	85.3%	CG6149	Exon	Unknown	Unknown		AIG1 (7) ADTRP (5)	ADTRP - Orofacial clefts (interaction).	N0; G0; FB0; R4; C0.
FBti0100449	78.1%	CG6332	Exon	Unknown	Unknown		THEG (4)	-	N0; G0; FB0; R4; C0.
FBti0100452	66.5%	CG7896	5'UTR intron	Unknown	Unknown		IGFALS (1)	Insulin-like growth factors, deficiency of acid-labile subunit.	N0; G0; FB0; R0; C1.
FBti0113990	88.4%	CG8483	Intron	Unknown	Unknown		PI16 (2)	Chemerin levels.	N0; G2; FB0; R0; C0.
FBti0075478	74.5%	Osi17	5'UTR	Unknown	Unknown		-	-	N0; G0; FB0; R0; C0.
FBti0100401	82.6%	CG13905	1.6kb upstream	Unknown	Unknown		-	-	N0; G4; FB0; R0; C0.
FBti0075309	79.2%	CG42789	1.2kb downstream	Unknown	Unknown		-	-	N0; G0; FB0; R0; C4.
FBti007815	82.7%	CG1387	400bp upstre	Unknown	Unknown		-	-	N0; G0; FB0;

8			am						R2; C0.
**List of resistant lines**									
**Insertion line**	**LT50 ratio (candidate/wild type)**	**Nearest Gene(s)**	**Insert location**	**Gene ontology**		**References**	**Human ortholog(s) (DIOPT scores)**	**Disease/Trait**	**Gene expression scores**
				**Molecular function**	**Biological process**				
**Defense related-physical barrier**									
FBti0075406	119.4%	Cpr51A	300bp upstream	Cuticular protein	Multicellular organism reproduction.		-	-	N1; G4; FB0; R2; C0.
FBti0075815	125.1%	CS-2	Intron	Chitin synthase activity	Unknown.		HAS1 (3)	-	N0; G2; FB0; R1; C0.
**Defense related- canonical immune system (humoral & cellular)**									
FBti0100386	112.4%	MED26	Exon	Mediator complex subunit 26	Phagocytosis; transcription regulation.	[24]	MED26 (6)	-	N3; G2; FB2; R4; C2.
FBti0100394	127.2%	ftz-f1	3'UTR	Transcription factor activity	Response to Juvenile hormone; RNA metabolic process; morphogenesis; phagocytosis.	RNAi reduce both escape/ replication and phagocytosis of *C. neoformans* [30].	NR5A2 (8) NR5A1 (7)	NR5A2 - Pancreatic cancer. NR5A1 - Adrenocortical insufficiency, premature ovarian failure, spermatogenic failure.	N2; G1; FB1; R3; C1.
FBti0100522	119.9%	CG10226	Intron	Drug transmembrane transporter activity	Response to methotrexate; immune response.	RNAi suppress activation of Dipt-lacZ by bacteria LPS [21].	ABCB11 (5) ABCB5 (4) ABCB1 (4) ABCB4 (4)	ABCB11 - Metabolic traits. ABCB5 - Dental caries. ABCB1 - Bowel disease. ABCB4 - Cholestasis intrahepatic.	N2; G4; FB0; R1; C0.
**Defense related- non-canonical immune system**									
FBti0075799	123.8%	CG7408	Exon	Acetylgalactosamine-4-sulfata	Metabolic process; defense response to bacterium.	[8]	ARSI (7) ARSJ (6) ARSB	ARSB - Hippocampal atrophy, mucopolysaccharidosis.	N1; G2; FB1; R1; C1.

				se activity			(6)		
FBti0075365	116.1%	mangetout	Intron	GPCR activity	Adult feeding behavior; response to insecticide; activate phospholipase C; defense response to bacterium.	RNAi decrease infection of *L. monocytogenes* [23].	GRM4 (4) GRM1 (3) GRM7 (3) GRM8 (3) GRM5 (3)	GRM1 - Spinocerebellar ataxia. GRM7 - Depressive disorder, orofacial clefts. GRM8 - Carotid atherosclerosis in HIV infection, chemerin levels, depression. GRM5 - Attention deficit hyperactivity disorder, tanning.	N2; G0; FB0; R0; C0.
FBti0101982	113.1%	CG10513	Exon	Transferase activity	Defense response to bacterium.	RNAi increase susceptible to bacterial infection [22].	-	-	N3; G4; FB0; R1; C1.
FBti0075343	108.5%	CG12105/CG18170/CG33791	Exon/3'UTR intron/intron	Unknown/unknown/oxoglutarate dehydrogenase activity.	Defense response to bacterium/unknown/tricarboxylic acid cycle.	RNAi increase susceptible to bacterial infection [22].	CG33791 - OGDH (5) OGDHL (5)	OGDH - Alpha-ketoglutarate, dehydrogenase deficiency.	N1/0/0; G1/0/0; FB0/0/0; R0/4/4; C2/0/0.
FBti0114235	121.6 %	CG13085/CG16771	5'UTR intron/3'UTR	Unknown/alkaline phosphatase activity	Unknown/metabolic process; defense response to bacterium.	RNAi increase susceptible to bacterial infection [22].	CG13085 - RA1 (1) CG16771 - ALPP (4) ALPL (4)	RA1 - Parkinson's disease, Smith-Magenis syndrome ALPP - Height. ALPL - Metabolic traits, hypophosphatasia, odontohypoph	N1/1; G1/2; FB1/2; R1/1; C1/1.

								osphatasia.	
FBti0078152	118.8%	CG14636	Exon	Unknown	Sensory perception of sound; defense response to bacterium	RNAi increase resistance to bacterial infection [22].	-	-	N1; G0; FB1; R1; C0.
FBti0075266	128.5%	CG32264/ppk27	Intron/1.5kb downstream	Unknown/sodium channel activity	Unknown/sodium ion transport; defense response to bacterium	RNAi increase susceptible to bacterial infection [22].	CG32264 - PHACTR2 (5) PHACTR4 (5)	PHACTR2 - Erectile dysfunction, bowel disease, lung cancer, obesity-related traits.	N3/0; G2/0; FB1/0; R2/0; C2/0.
FBti0077729	121.8%	CG8180	Intron	Unknown	Regulation of JAK/STAT signaling.	RNAi decrease JAK/STAT signaling [20].	-	-	N1; G3; FB0; R3; C1.
FBti0077734	115.1%	CG15625	70bp upstream	Unknown	Defense response to bacterium.	RNAi increase susceptible to bacterial infection [22].	SAMD15 (4)	-	N0; G0; FB0; R2; C0.
FBti0076230	117.1%	CG9733	Intron	Serine endopeptidase activity.	Proteolysis; defense response.		-	-	N0; G0; FB0; R0; C0.
FBti0075272	108.4%	CG5909	1.1kb upstream	Serine endopeptidase activity	Proteolysis; defense response.	Gene expression up regulated in flies infected with *Beauveria* [25].	-	-	N0; G1; FB0; R0; C2.
**List of other resistant lines:**									
FBti0078139	121.0%	Cdc42-interacting protein 4	3'UTR	Rho GTPase binding	Morphogenesis; negative regulation of actin filament polymerization, synaptic growth at neuromuscular junction, and actin		TRIP10 (8) FNBP1L (8)	-	N2; G2; FB1; R2; C1.

					nucleation.				
FBti0101677	122.1 %	Dhc93AB	Exon	Motor activity	Sound perception; microtubule-based movement.		DNAH9 (9) DNAH11 (6) DNAH17 (6)	DNAH9 - Pathological myopia. DNAH11 - LDL cholesterol, multiple cancers, ciliary dyskinesia.	N0; G0; FB0; R0; C0.
FBti0101590	117.4 %	Ankyrin	Intron	Structural constituent of cytoskeleton	Cytoskeletal anchoring at plasma membrane; signal transduction.		ANK1(6) ANK3 (5) ANK2 (4)	ANK2 - Cancer, cardiac arrhythmia. ANK3 - Autism spectrum disorder, bipolar disorder, schizophrenia, major depressive disorder. ANK1 - Pulmonary function decline, diabetes, Spherocytosis. FBLN5 - Height, Cutis laxa, Macular degeneration.	N3; G3; FB2; R3; C2.
FBti0100408	117.4 %	still life	Intron	GEF activity	Axonogenesis; actin cytoskeleton organization; Rac GTPase activity; filopodium assembly.		TIAM1 (5)	Renal sinus fat.	N4; G1; FB1; R1; C1.
FBti0075319	125.5 %	Nijc/CG14391	160bp upstream/120bp upstream	Ninjurin C /unknown	Cell adhesion, tissue regeneration /unknown.		Nijc – NINJ1 (8) NINJ2 (6)	NINJ2 - Stroke.	N0/0; G1/0; FB0/0; R1/3; C1/0.
FBti0075829	114.6 %	Dh31-R	5'UTR intron	Diuretic hormone receptor activit	GPCRsignaling pathway; Malpighian tubule function.		CALCRL (8) CALCR (5)	CALCR - Involutional osteoporosis.	N2; G3; FB0; R0; C1.

				y					
FBti0075649	116.5 %	CG31760	Intron	Glutamate receptor activity	Gamma-aminobutyric acid signaling pathway.		GPR179 (4)	Ovarian cancer, congenital stationary night blindness.	N3; G0; FB1; R2; C0.
FBti0075434	126.1 %	cv-c	3'UTR	Rho GTPase activator activity	Digestive tract development; epidermal growth factor receptor signaling pathway; synaptic transmission; morphogenesis.		DLC1 (5) STARD13 (5)	DLC1 - Bipolar disorder, obesity-related traits, colorectal cancer. STARD13 - Intracranial aneurysm.	N1; G1; FB1; R1; C0.
FBti0076178	107.5 %	CG32206	Intron	Wnt-activated receptor activity	Canonical Wnt signaling pathway.		CDCP2 (1)	-	N2; G0; FB1; R0; C0.
FBti0075473	139.1 %	Synaptotagmin 7	Exon	Transporter activity	Synaptic vesicle exocytosis.		SYT7 (7)	-	N4; G0; FB1; R1; C1.
FBti0077565	117.8 %	CG11155	3'UTR	Glutamate-gated ion channel activity	Ion transport; neuron projection morphogenesis.		GRIK4 (3) GRIK5 (3) GRIK2 (3)	GRIK4 - Cognitive performance. GRIK5 - Bipolar disorder. GRIK2 - Cardiac hypertrophy, obesity-related traits, obsessive compulsive disorder, mental retardation.	N2; G2; FB1; R1; C1.
FBti0076174	121.0 %	Synaptotagmin β	1kb upstream	Transporter activity	Synaptic vesicle exocytosis.		SYT6 (1) SYT4 (1) SYT11 (1) SYT9 (1)	SYT6 - Breast cancer, obesity-related traits. SYT4 and SYT11- Parkinson's disease.	N1; G0; FB0; R1; C0.
FBti0078348	118.8 %	CG13793	Exon	Sodium sympo	Neurotransmitter transport.		-	-	N0; G2; FB1; R1; C2.

				rter activity					
FBti0100402	121.2 %	Neurexin IV	Exon	Transmembrane signaling receptor activity	Exocytosis; synaptic target recognition; cell junction organization; endothelial cell development.		CNTNAP2 (9) CNTNAP3 (6)	CNTNAP2 - Alzheimer's disease, schizophrenia, bipolar disorder, bone mineral density, obesity-related traits.	N2; G2; FB2; R2; C2.
FBti0075300	109.3 %	POU domain protein 2	Exon	Transcription factor activity	Ectoderm development; neuroblast development.		POU2F2 (4) POU2F3 (4) POU2F1 (3)	POU2F1 - Liver enzyme levels.	N1; G1; FB0; R0; C0.
FBti0077627	118.0 %	shaven	Intron	Transcription factor activity	Neuron development; muscle organ development; transcription regulation.		PAX5 (6) PAX2 (6)	PAX5 - Intelligence, obesity and blood pressure. PAX2 - Alzheimer's disease, papillorenal syndrome, renal adysplasia, urogential adysplasia.	N1; G0; FB0; R0; C0.
FBti0076233	113.8 %	Sema-1a	5'UTR	Semaphorin receptor binding	Neuron projection guidance; brain morphogenesis; synapse assembly; locomotory behavior.		SEMA6A (3)	Allergic rhinitis, amyotrophic lateral sclerosis.	N2; G0; FB0; R1; C0.
FBti0127615	120.7 %	CG5213	50bp downstream	mRNA binding	Neurogenesis.		ELAVL2 (1) ELAVL4 (1)	ELAVL2- Response to platinum-based chemotherapy in non-small-cell lung cancer. ELAVL4- Obesity-related traits.	N0; G0; FB0; R2; C0.
FBti0075715	117.8 %	Nemo	5'UTR	MAP kinase activity	Synaptic growth at neuromuscular junction;		NLK (10)	-	N3; G2; FB1; R3; C1.

					morphogenesis; protein phosphorylation.				
FBti0075708	121.8 %	atilla/arrest	5'UTR/5'UTR intron	Unknown/mRNA binding	Unknown/ negative regulation of translation; morphogenesis; oogenesis.		arrest - CELF2 (8) CELF1 (8)	CELF2 - Alzheimer's disease. CELF1 - Body mass index.	N2/1; G4/3; FB3/3; R4/4; C4/2.
FBti0075502	119.8 %	CG11902	Exon	Nucleic acid binding	Unknown.		ZNF423 (1) ZNF664 (1)	ZNF423 - Inattentive symptoms, Joubert syndrome, nephronophthisis. ZNF664 - HDL cholesterol.	N1; G1; FB1; R2; C1.
FBti0077700	115.0 %	CG13287	4kb upstream	Transcription factor activity	Transcription regulation.		PRDM8 (6)	Diastolic blood pressure.	N1; G0; FB0; R0; C0.
FBti0075382	121.7 %	pgc	3'UTR	Unknown	Negative regulation of transcription; germ cell development.		-	-	N-; G-; FB-; R-; C-.
FBti0113997	121.5 %	toy	Intron	Transcription factor activity	Transcription regulation; mushroom body development.		PAX6 (9)	Body mass index, corneal dystrophy, cataract, coloboma, foveal hyperplasia, Gillespie syndrome, Keratitis, Peters anomaly.	N3; G0; FB0; R1; C0.
FBti0076307	116.3 %	CG7804	3'UTR	mRNA binding	Unknown.		TARDBP (7)	Ewing sarcoma, baldness, amyotrophic lateral sclerosis, frontotemporal lobar degeneration.	N0; G1; FB0; R2; C0.
FBti0076228	122.2 %	Rbp6	5'UTR	mRNA-binding	Unknown.		MSI2 (8)	-	N2; G0; FB0; R0; C0.

FBti0100392	116.7%	CG9850	Intron	Metalloendopeptidase activity	Proteolysis; cell proliferation.		ADAMTS5 (1)	Dialysis-related mortality.	N1; G2; FB0; R1; C0.
FBti0077724	125.1%	CG14127/CG34420	Exon/intron	Unknown/dipeptidase activity	Cilium movement; axonemal dynein complex assembly/proteolysis.		CG14127 - CCDC151 (8) CG34420 - DPEP2 (5)	-	N0/-; G0/-; FB0/-; R0/-; C0/-.
FBti0100410	117.6%	Ac76E	Intron	Adenylate cyclase activity	cAMP biosynthetic process; response to starvation; negative regulation of multicellular organism growth.		ADCY2 (8) ADCY7 (6)	ADCY2 - Visceral fat, capecitabine sensitivity. ADCY7 - Bowel disease.	N2; G2; FB2; R2; C2.
FBti0100462	126.0%	CG8046	Exon	Glycerophosphate dehydrogenase activity	Transmembrane transport; glycerol-3-phosphate metabolic process.		SLC46A2 (3) SLC46A1 (3)	SLC46A1 - Folate malabsorption hereditary.	N0; G0; FB0; R0; C0.
FBti0075412	123.4%	CG15661	5'UTR	Glucuronosyltransferase activity	Metabolic process.		UGT2B7 (3)	Obesity-related traits.	N2; G3; FB2; R2; C2.
FBti0075763	111.0%	CG15629	Exon	Oxidoreductase activity	Metabolic process.		RDH10 (6) SDR16C5 (5)	RDH10 - Bowel disease. SDR16C5 - Height.	N1; G0; FB0; R1; C1.
FBti0078451	122.2%	Prosβ4R2	Intron	Threonine endopeptidase activity	Ubiquitin-dependent protein catabolic process.		PSMB2 (7)	-	N0; G0; FB0; R1; C0.
FBti0078155	119.9%	CG33093	Exon	Oxidoreductase activity	Oxidation-reduction process		-	-	N0; G0; FB0; R0; C0.

FBti0076197	124.0%	CG12229/G32176	Exon/intron	Pyruvate kinase activity/unknown	Glycolytic process/Unknown.		CG12229 - PKM (2) CG32176 - CAAP1 (2)	-	N0/2; G0/1; FB0/1; R4/2; C0/1.
FBti0075818	125.8%	Hs3st-A	5'UTR intron	3-OST-1 activity	Unknown.		HS3ST5 (6) HS3ST1 (5)	HS3ST1 - Nonalcoholic fatty liver disease.	N3; G1; FB0; R1; C0.
FBti0075691	113.2%	CG31637	Intron	Sulfotransferase activity	Unknown.		CHST1 (7) CHST4 (5) CHST6 (5)	CHST6 - Macular corneal dystrophy.	N2; G2; FB2; R2; C2.
FBti0075786	118.8%	CG12090	500bp upstream	GTPase activator activity	Unknown.		DEPDC5 (8)	Chronic hepatitis C infection, intelligence, epilepsy.	N2; G1; FB2; R2; C1.
FBti0115873	117.3%	CG12289	2kb downstream	Ketohexokinase activity	Unknown.		KHK (7)	Fructosuria.	N0; G0; FB0; R2; C0.
FBti0078351	121.3%	CG9451	Exon	Phosphatase activity	Unknown.		ACP2 (2)	Lysosomal acid phosphatase deficiency.	N1; G2; FB4; R3; C3.
FBti0100491	126.7%	Ir93a	Intron	Ionotropic glutamate receptor activity	Detection of chemical stimulus involved in sensory perception.		-	-	N0; G0; FB0; R0; C0.
FBti0075621	124.5%	dpr20	Intron	Unknown	Sensory perception of chemical stimulus.		-	-	N3; G0; FB0; R0; C0.
FBti0077765	120.0%	Spinophilin	3'UTR	Protein phosphatase 1 binding	Olfactory behavior; inter-male aggressive behavior.		PPP1R9A (7)	-	N3; G2; FB0; R2; C0.
FBti0114248	117.6%	CG31229	Intron	Transporter activit	Protein import into mitochondrial		TIMM22 (10)	-	N2; G2; FB2; R2; C1.

				y	inner membrane.				
FBti0078228	118.1%	Ncc69	Intron	Sodium chloride symporter activity	Chloride, potassium, rubidium, and amino acid transmembrane transport.		SLC12A2 (9) SLC12A1 (6)	SLC12A2 - Schizophrenia. SLC12A1 - Bartter syndrome.	N2; G2; FB0; R2; C2.
FBti0075553	115.5%	CG14855	Exon	Transporter activity	Transmembrane transport.		SLC22A14 (1)	-	N1; G0; FB0; R0; C0.
FBti0075329	113.2%	CG31030	110bp upstream	Proton-transporting ATPase activity	ATP hydrolysis coupled proton transport.		ATP6AP1L (2)	-	N4; G0; FB0; R1; C1.
FBti0075303	115.4%	ZnT63C	5'UTR intron	Zinc transporter activity	Zinc ion transmembrane transport		SLC30A1 (6) SLC30A10 (5)	SLC30A1 - Coronary heart disease. SLC30A10 - Hypermagnesemia with dystonia, polycythemia, and cirrhosis.	N2; G3; FB2; R2; C2.
FBti0075354	119.9%	CG10950	1.6kb upstream	Transporter activity	Protein import into nucleus		TNPO3 (1)	Primary biliary cirrhosis, systemic lupus erythematosus, ulcerative colitis.	N0; G0; FB0; R2; C0.
FBti0077645	114.0%	plexus	Intron	Unknown	Male aggressive behavior; morphogenesis.		SRF (9)	-	N1; G2; FB2; R2; C2.
FBti0076206	110.9%	hebe	5'UTR	Unknown	Determination of adult lifespan.		-	-	N2; G3; FB2; R3; C2.
FBti0076231	117.0%	CG10440	40bp upstream	Unknown	Protein homooligomerization.		KCTD15 (9) KCTD1 (6)	KCTD15 - Major depressive disorder, obesity-related traits. KCTD1 - Sudden cardiac arrest.	N2; G0; FB0; R1; C0.
**Non protein coding gene**									

FBti0076291	126.6%	CR44665	Exon	Unknown	Unknown		-	-	N-; G-; FB-; R-; C-.
FBti0101676	116.8%	CR44260	800bp upstream	Unknown	Unknown		-	-	N-; G-; FB-; R-; C-.
FBti0075783	122.7%	hsr-omega	Exon	unknown	Oogenesis; regulation of JNK cascade; response to heat.		-	-	N-; G-; FB-; R-; C-.
FBti0101663	119.5%	CR45457	Exon	Unknown	Unknown		-	-	N0; G0; FB0; R2; C0.
**Unknown**									
FBti0101673	119.8%	CG4751	3'UTR	Unknown	Unknown.		MPND (6)	-	N2; G2; FB1; R2; C1.
FBti0115852	120.0%	CG12814	Intron	Unknown	Unknown		-	-	N0; G2; FB1; R1; C1.
FBti0114158	111.7%	CG13428	Exon	Unknown	Unknown		-	-	N0; G1; FB0; R0; C4.
FBti0075297	116.1%	CG2010	Intron	Unknown	Unknown		FBXL12 (1)	-	N0; G2; FB0; R2; C2.
FBti0075772	114.2%	CG31128	Exon	Unknown	Unknown		-	-	N0; G0; FB0; R3; C0.
FBti0077637	112.2%	CG31191	5'UTR intron	Unknown	Unknown		-	-	N2; G0; FB1; R0; C0.
FBti0078347	127.7%	CG31211	3'UTR	Unknown	Unknown		PNISR (7)	-	N3; G1; FB1; R2; C1.
FBti0075271	125.0%	CG31663	Intron	Unknown	Unknown		MFSD6L (2)	-	N3; G4; FB0; R1; C1.
FBti0113546	120.9%	CG6983	Intron	Unknown	Unknown		C20orf27(7)	-	N2; G4; FB1; R2; C1.
FBti0115879	118.2%	CG7337	Intron	Unknown	Unknown		WDR62 (7)	Microcephaly.	N2; G2; FB2; R2; C2.
FBti0075749	118.5	CG15140	Exon	Unknown	Unknown		-	-	N0; G0; FB0; R0; C0.

	%								
FBti0076207	109.4 %	CG43120	Exon	Unknown	Unknown		-	-	N-; G-; FB-; R-; C-.
FBti0128240	116.8 %	CG15080	1kb upstream	Unknown	Unknown		NMU (1)	-	N0; G0; FB0; R0; C0.
FBti0101653	113.4 %	CG14540	3'UTR	Unknown	Unknown		-	-	N0; G0; FB0; R4; C0.

Supplementary Table S2: Subset of lines tested for fungal life history components and climbing assay.

Susceptible lines	Name of gene(s)	Molecular function	Female LT50 (days)	Female LT50 ratio (candidates/ wild type)	Male LT50 (days)	Male LT50 ratio (candidates/ wild type)	Climbing ability (slop)
FBti0075259	Peroxidasin	Peroxidase activity	3.37	81.9%	4.24	85.1%	0.28
FBti0075265	srpk79D	Protein serine threonine kinase activity	3.25	76.5%	5.13	97.3%	0.15
FBti0075291	RhoGEF3	RhoGEF activity	3.16	77.0%	3.89	86.2%	0.30
FBti0075299	CG13676	Chitin binding	3.51	85.4%	4.57	90.0%	0.20
FBti0075318	Octbeta2R	Octopamine receptor activity	3.34	78.6%	4.27	81.1%	0.32
FBti0075320	S-Lap3	Aminopeptidase activity	3.14	73.9%	4.47	84.8%	0.18
FBti0075347	Neuroligin 1	Neurexin family protein binding	3.15	87.5%	3.63	86.1%	0.12
FBti0075396	CG31140	NAD+ kinase activity	3.40	80.0%	4.29	81.5%	0.26
FBti0075469	amon	Serine endopeptidase activity	3.62	85.2%	4.75	90.3%	0.31
FBti0075486	No affected gene		3.50	82.3%	4.42	84.0%	0.18
FBti0075739	Caupolican	Transcription factor activity	3.23	78.6%	3.71	73.9%	0.28
FBti0075743	Oamb	Octopamine receptor activity	3.42	80.3%	4.33	82.3%	0.22
FBti0075754	Lnk	Signal transducer activity	3.25	76.3%	3.66	69.4%	0.26
FBti0075765	Jaguar	Myosin protein	3.20	75.1%	4.55	86.4%	0.13

FBti0075771	lobo	Unknown	3.21	89.2%	3.62	85.7%	0.10
FBti0075792	CG3822	Glutamate receptor activity	3.23	75.9%	4.55	86.4%	0.18
FBti0075807	CG3940	Carbonate dehydratase activity	3.31	77.8%	3.50	66.5%	0.16
FBti0075810	Multiplexin	Carbohydrate binding	3.33	92.7%	3.55	84.0%	0.11
FBti0076223	Jon65Aii	Serine endopeptidase activity	3.17	74.5%	4.30	81.6%	0.20
FBti0076229	Cht7	Chitinase activity	3.37	82.1%	4.14	81.4%	0.27
FBti0076243	DCX-EMAP	Microtubule binding	3.27	76.9%	4.33	82.3%	0.12
FBti0076261	zormin	Structural constituent of cytoskeleton	3.49	81.9%	4.66	88.6%	0.17
FBti0076404	No affected gene		3.12	73.3%	3.86	73.3%	0.19
FBti0100418	CG45186	Actin binding	3.36	79.0%	4.43	84.2%	0.27
FBti0100425	CG14691	Unknown	3.16	74.2%	4.35	82.6%	0.23
FBti0100426	Alphabet	Protein serine threonine phosphatase activity	3.51	82.6%	4.01	76.2%	0.33
FBti0100452	CG7896	Unknown	3.14	73.7%	4.17	79.1%	0.06
FBti0100458	CG32198	Unknown	3.16	74.2%	4.18	79.4%	0.16
FBti0100466	Hemolectin	Chitin binding	3.30	80.2%	4.38	88.5%	0.15
Resistant lines	Name of gene(s)	Molecular function	Female LT50	Female LT50 ratio (candidates/ wild type)	Male LT50	Male LT50 ratio (candidates/ wild type)	Climbing ability (slop)
FBti0075266	CG32264/ppk27	Unknown/sodium channel activity	4.84	123.0%	6.44	139.7%	0.25
FBti0075271	CG31663	Unknown	4.66	118.3%	6.41	139.2%	0.30
FBti0075319	Nijc/CG14391	Ninjurin C /unknown	4.60	116.8%	5.92	128.4%	0.19
FBti0075382	pgc	Unknown	4.28	108.6%	5.79	125.6%	0.19
FBti0075406	Cpr51A	Cuticular protein	4.53	115.1%	5.93	128.6%	0.17
FBti0075412	CG15661	Glucuronosyltransferase activity	4.97	126.2%	5.74	124.6%	0.26
FBti0075434	cv-c	Rho GTPase activator activity	4.80	122.0%	6.97	151.3%	0.25
FBti0075783	hsr-omega	Unknown	4.69	119.0%	7.57	164.3%	0.19
FBti0075786	CG12090	GTPase activator activity	5.04	127.9%	5.78	125.5%	0.14

FBti0075799	CG7408	Acetylgalactosamine-4-sulfatase activity	4.70	119.3%	5.91	128.3%	0.21
FBti0075815	CS-2	Chitin synthase activity	4.43	112.6%	5.88	127.5%	0.18
FBti0075818	Hs3st-A	3-OST-1 activity	4.88	123.9%	5.33	115.7%	0.17
FBti0076172	No affected gene		4.55	115.6%	5.30	114.9%	0.26
FBti0076174	Synaptotagmin β	Transporter activity	4.62	117.4%	5.99	130.0%	0.29
FBti0076197	CG12229/CG32176	Pyruvate kinase activity/unknown	4.15	105.3%	5.86	127.1%	0.32
FBti0076202	No affected gene		4.93	125.2%	5.77	125.2%	0.22
FBti0076228	Rbp6	mRNA-binding	4.31	109.4%	5.59	121.2%	0.26
FBti0076291	CR44665	Unknown	4.18	106.2%	6.16	133.6%	0.35
FBti0077724	CG14127/CG34420	Unknown/dipeptidase activity	5.15	130.9%	6.73	146.1%	0.28
FBti0077729	CG8180	Unknown	4.81	122.2%	6.20	134.6%	0.28
FBti0100394	ftz-f1	Transcription factor activity	4.81	122.1%	6.46	140.1%	0.13
FBti0100402	Neurexin IV	Transmembrane signaling receptor activity	4.53	115.1%	5.58	121.1%	0.28
FBti0100408	still life	GEF activity	4.76	120.9%	6.24	135.4%	0.26
FBti0100410	Ac76E	Adenylate cyclase activity	5.10	129.4%	5.48	118.9%	0.30
FBti0100462	CG8046	Glycerophosphate dehydrogenase activity	4.75	120.6%	5.61	121.7%	0.27

Supplementary Table S3: LT50 comparison between the control line (cnbw), and Dif mutant flies. LT50 values obtained from four repeats were averaged (C Avg and Dif Avg).

C-1	C-2	C-3	C-4	Dif1	Dif2	Dif3	Dif4	C Avg	C SD	Dif Avg	Dif SD
4.024	4.199	4.065	4.033	4.111	4.083	3.917	4.032	4.080	.0811	4.036	.0857

Supplementary Table S4: List of all tested Mi{ET1} insertion lines. The lines were generated as part of The *Drosophila* Gene Disruption Project[58]

**Flybase ID**	**Stock information**
FBti0075253	22674 y[1] w[67c23]; Mi{ET1}fas[MB00027] CG6220[MB00027]
FBti0075254	22694 y[1] w[67c23]; Mi{ET1}MB00078
FBti0075255	22747 y[1]; Mi{ET1}Slip1[MB00262]

FBti0075256	22791 y[1] w[67c23]; Mi{ET1}MB00408
FBti0075257	22794 y[1] w[67c23]; Mi{ET1}MB00427
FBti0075258	22796 y[1] w[67c23]; Mi{ET1}msn[MB00430]
FBti0075259	22805 y[1] w[67c23]; Mi{ET1}Pxn[MB00459]
FBti0075260	22809 y[1] w[67c23]; Mi{ET1}CG34114[MB00468]
FBti0075261	22812 y[1] w[67c23]; Mi{ET1}CkIIα[MB00477]/TM3, Sb[1] Ser[1]
FBti0075262	22813 y[1] w[67c23]; Mi{ET1}Rab23[MB00481]/TM3, Sb[1] Ser[1]
FBti0075263	22815 y[1] w[67c23]; Mi{ET1}MB00489
FBti0075265	22844 y[1] w[67c23]; Mi{ET1}srpk79D[MB00578]
FBti0075266	22847 y[1] w[67c23]; Mi{ET1}CG32264[MB00592] CG10858[MB00592]
FBti0075267	22850 y[1] w[67c23]; Mi{ET1}CG33970[MB00606]
FBti0075268	22853 y[1] w[67c23]; Mi{ET1}MB00615
FBti0075269	22854 y[1] w[67c23]; Mi{ET1}CG15820[MB00627]
FBti0075270	22855 y[1] w[67c23]; Mi{ET1}Slc45-1[MB00630]
FBti0075271	22857 y[1] w[67c23]; Mi{ET1}CG31663[MB00634]
FBti0075272	22861 y[1] w[67c23]; Mi{ET1}MB00654
FBti0075273	22865 y[1] w[67c23]; Mi{ET1}CG7352[MB00667]
FBti0075274	22867 y[1]; Mi{ET1}NfI[MB00672]
FBti0075275	22868 y[1] w[67c23]; Mi{ET1}PKD[MB00674]
FBti0075276	22874 y[1] w[67c23]; Mi{ET1}MB00695
FBti0075277	22884 y[1] w[67c23]; Mi{ET1}nmo[MB00735]
FBti0075278	22888 y[1] w[67c23]; Mi{ET1}MB00746
FBti0075279	22889 y[1] w[67c23]; Mi{ET1}CG9602[MB00748]/TM6C, cu[1] Sb[1]
FBti0075280	22890 y[1] w[67c23]; Mi{ET1}CG7816[MB00749]
FBti0075281	22891 y[1] w[67c23]; Mi{ET1}CG13830[MB00767]
FBti0075282	22893 y[1] w[67c23]; Mi{ET1}MB00769
FBti0075283	22894 y[1] w[67c23]; Mi{ET1}Dscam4[MB00771]
FBti0075284	22896 y[1] w[67c23]; Mi{ET1}CG4221[MB00781]
FBti0075285	22898 y[1] w[67c23]; Mi{ET1}MB00787
FBti0075286	22899 y[1] w[67c23]; Mi{ET1}R[MB00790]
FBti0075287	22900 y[1] w[67c23]; Mi{ET1}MB00799
FBti0075288	22901 y[1] w[67c23]; Mi{ET1}TfAP-2[MB00802]
FBti0075289	22904 y[1] w[67c23]; Mi{ET1}Ptp61F[MB00813]
FBti0075290	22905 y[1] w[67c23]; Mi{ET1}CG42788[MB00815]
FBti0075291	22907 y[1] w[67c23]; Mi{ET1}RhoGEF3[MB00829]
FBti0075292	22908 y[1] w[67c23]; Mi{ET1}MB00834
FBti0075293	22910 y[1] w[67c23]; Mi{ET1}CrzR[MB00838]
FBti0075295	22913 y[1] w[67c23]; Mi{ET1}CG17181[MB00844]/TM3, Sb[1] Ser[1]
FBti0075297	22917 y[1] w[67c23]; Mi{ET1}CG2010[MB00855]

FBti0075298	22918 y[1] w[67c23]; Mi{ET1}MB00860
FBti0075299	22921 y[1] w[67c23]; Mi{ET1}CG13676[MB00880]/TM3, Sb[1] Ser[1]
FBti0075300	22925 y[1] w[67c23]; Mi{ET1}pdm2[MB00894]
FBti0075301	22931 y[1] w[67c23]; Mi{ET1}CG43427[MB00912]
FBti0075302	22953 y[1] w[67c23]; Mi{ET1}MB00985
FBti0075303	22963 y[1] w[67c23]; Mi{ET1}ZnT63C[MB01014]
FBti0075304	22980 y[1] w[67c23]; Mi{ET1}MB01062
FBti0075305	22984 w[1118] Mi{ET1}CG14234[MB01077]
FBti0075306	22988 w[1118] Mi{ET1}cngl[MB01092]
FBti0075307	22992 w[1118] Mi{ET1}CG9281[MB01100]
FBti0075308	23001 y[1] w[67c23]; Mi{ET1}sowah[MB01147]
FBti0075309	23002 y[1] w[67c23]; Mi{ET1}MB01153
FBti0075310	23008 y[1] w[67c23]; Mi{ET1}sima[MB01168]
FBti0075311	23009 y[1] w[67c23]; Mi{ET1}MB01173
FBti0075312	23014 y[1] w[67c23]; Mi{ET1}MB01186
FBti0075313	23015 y[1] w[67c23]; Mi{ET1}MB01188
FBti0075314	23017 y[1] w[67c23]; Mi{ET1}MB01198
FBti0075315	23019 y[1] w[67c23]; Mi{ET1}CG34354[MB01203]
FBti0075316	23021 y[1] w[67c23]; Mi{ET1}MB01218
FBti0075317	23022 y[1] w[67c23]; Mi{ET1}VGAT[MB01219]/CyO
FBti0075318	23040 y[1] w[67c23]; Mi{ET1}MB01266/TM3, Sb[1] Ser[1]
FBti0075319	23045 y[1] w[67c23]; Mi{ET1}MB01283
FBti0075320	23055 y[1] w[67c23]; Mi{ET1}S-Lap3[MB01319]
FBti0075321	23056 y[1] w[67c23]; Mi{ET1}CG14853[MB01320]
FBti0075322	23057 y[1] w[67c23]; Mi{ET1}Usp12-46[MB01322]
FBti0075323	23059 y[1] w[67c23]; Mi{ET1}CG7720[MB01330]
FBti0075324	23061 y[1] w[67c23]; Mi{ET1}MB01333
FBti0075326	23064 y[1] w[67c23]; Mi{ET1}MB01342
FBti0075327	23066 y[1] w[67c23]; Mi{ET1}5-HT7[MB01344]
FBti0075328	23068 y[1] w[67c23]; Mi{ET1}MB01349
FBti0075329	23069 y[1] w[67c23]; Mi{ET1}MB01350
FBti0075330	23072 y[1] w[67c23]; Mi{ET1}CG6938[MB01354]
FBti0075331	23077 y[1] w[67c23]; Mi{ET1}MB01366
FBti0075332	23080 y[1] w[67c23]; Mi{ET1}CG34391[MB01371]
FBti0075333	23082 y[1] w[67c23]; Mi{ET1}Takr86C[MB01381]
FBti0075335	23086 y[1] w[67c23]; Mi{ET1}MB01390
FBti0075336	23087 y[1] w[67c23]; Mi{ET1}CG11073[MB01677]
FBti0075337	23088 y[1] w[67c23]; Mi{ET1}CG3608[MB01727] CG4741[MB01727]
FBti0075338	23090 y[1] w[67c23]; Mi{ET1}MB01740

FBti0075339	23091 y[1] w[67c23]; Mi{ET1}MB01788
FBti0075340	23172 y[1] w[67c23]; Mi{ET1}CG14989[MB00293]/TM3, Sb[1] Ser[1]
FBti0075341	23726 y[1] w[67c23]; Mi{ET1}CG14301[MB00673]
FBti0075342	23728 y[1]; Mi{ET1}Lin29[MB00729]
FBti0075343	23173 y[1] w[67c23]; Mi{ET1}CG33791[MB00772] CG12105[MB00772] CG18170[MB00772]
FBti0075344	23174 y[1]; Mi{ET1}ey[MB00784]
FBti0075346	23730 y[1] w[67c23]; Mi{ET1}MB00845
FBti0075347	23176 y[1] w[67c23]; Mi{ET1}Nlg1[MB00987]
FBti0075348	24453 y[1]; Mi{ET1}CG11148[MB01094]
FBti0075349	23731 y[1] w[67c23]; Mi{ET1}MB01144
FBti0075350	23177 y[1] w[67c23]; Mi{ET1}CecC[MB01213]
FBti0075351	23732 w[1118] Mi{ET1}Cda4[MB01337]
FBti0075352	23178 y[1] w[67c23]; Mi{ET1}AR-2[MB01405]
FBti0075353	23733 y[1] w[67c23]; Mi{ET1}Ptp52F[MB01407]
FBti0075354	23179 y[1] w[67c23]; Mi{ET1}MB01408
FBti0075355	23734 y[1] w[67c23]; Mi{ET1}MB01410
FBti0075356	23180 y[1] w[67c23]; Mi{ET1}CG34393[MB01412]
FBti0075357	23181 y[1] w[67c23]; Mi{ET1}CG42540[MB01413]
FBti0075358	23735 y[1] w[67c23]; Mi{ET1}Pde1c[MB01415]
FBti0075359	23736 y[1] w[67c23]; Mi{ET1}CG30263[MB01421]/CyO
FBti0075360	23182 y[1] w[67c23]; Mi{ET1}CG31708[MB01431]
FBti0075361	23183 w[1118] Mi{ET1}MB01433
FBti0075362	23184 w[1118] Mi{ET1}MB01437
FBti0075363	23185 Mi{ET1}CG3078[MB01438] w[1118]
FBti0075364	23186 y[1] w[67c23]; Mi{ET1}Osi21[MB01450]
FBti0075365	23737 y[1] w[67c23]; Mi{ET1}mtt[MB01452]
FBti0075366	23738 y[1] w[67c23]; Mi{ET1}MB01457
FBti0075367	23739 y[1] w[67c23]; Mi{ET1}MB01460
FBti0075368	23187 y[1] w[67c23]; Mi{ET1}CG11374[MB01461]
FBti0075369	23740 y[1] w[67c23]; Mi{ET1}CG44434[MB01463] CG44435[MB01463]
FBti0075370	23188 y[1] w[67c23]; Mi{ET1}MB01465
FBti0075371	23189 w[1118] Mi{ET1}org-1[MB01466]
FBti0075372	23190 w[1118] Mi{ET1}MB01490
FBti0075373	23191 y[1] w[67c23]; Mi{ET1}CG4341[MB01502]
FBti0075374	23192 y[1] w[67c23]; Mi{ET1}MB01528
FBti0075375	23193 y[1] w[67c23]; Mi{ET1}Sr-CI[MB01539]
FBti0075376	23194 y[1] w[67c23]; Mi{ET1}CG30015[MB01543] CG30015[MB01543]
FBti0075377	23195 y[1] w[67c23]; Mi{ET1}CG17350[MB01546]
FBti0075378	23196 y[1] w[67c23]; Mi{ET1}Glut1[MB01560]

FBti0075379	23742 y[1] w[67c23]; Mi{ET1}MB01562
FBti0075380	23743 y[1] w[67c23]; Mi{ET1}MB01563
FBti0075381	23744 w[1118] Mi{ET1}dnc[MB01572]
FBti0075382	23197 y[1] w[67c23]; Mi{ET1}pgc[MB01573]
FBti0075383	23745 y[1] w[67c23]; Mi{ET1}MB01583
FBti0075384	23746 y[1] w[67c23]; Mi{ET1}MB01591
FBti0075385	23198 y[1] w[67c23]; Mi{ET1}CG1136[MB01597]
FBti0075386	23199 y[1] w[67c23]; Mi{ET1}CG31145[MB01598]
FBti0075387	23747 y[1] w[67c23]; Mi{ET1}CG4053[MB01609]
FBti0075388	23200 y[1] w[67c23]; Mi{ET1}CG4329[MB01614]
FBti0075389	23201 y[1] w[67c23]; Mi{ET1}MB01615
FBti0075390	23202 y[1] w[67c23]; Mi{ET1}lola[MB01619]
FBti0075392	23748 y[1] w[67c23]; Mi{ET1}MB01625
FBti0075393	23204 y[1] w[67c23]; Mi{ET1}MB01627
FBti0075394	23205 y[1] w[67c23]; Mi{ET1}spag4[MB01631]
FBti0075395	23749 y[1] w[67c23]; Mi{ET1}CG31121[MB01633]
FBti0075396	23750 y[1] w[67c23]; Mi{ET1}CG31140[MB01635]/TM3, Sb[1] Ser[1]
FBti0075397	23206 y[1] w[67c23]; Mi{ET1}MB01636
FBti0075398	23751 y[1] w[67c23]; Mi{ET1}CG6231[MB01639]
FBti0075399	23752 y[1] w[67c23]; Mi{ET1}CG31752[MB01647]
FBti0075400	23753 y[1] w[67c23]; Mi{ET1}MB01653
FBti0075401	23207 w[1118] Mi{ET1}MB01657
FBti0075402	23754 y[1] w[67c23]; Mi{ET1}MB01663
FBti0075403	23208 w[1118] Mi{ET1}mmd[MB01664]
FBti0075404	23209 w[1118] Mi{ET1}MB01694
FBti0075405	23210 w[1118] Mi{ET1}flw[MB01707]
FBti0075406	23755 y[1] w[67c23]; Mi{ET1}MB01712
FBti0075407	23757 y[1] w[67c23]; Mi{ET1}GluRIIA[MB01746]
FBti0075408	23211 y[1] w[67c23]; Mi{ET1}MB01749
FBti0075409	23212 y[1] w[67c23]; Mi{ET1}CG31751[MB01760]
FBti0075410	23758 y[1] w[67c23]; Mi{ET1}CG30103[MB01774]
FBti0075412	23759 y[1] w[67c23]; Mi{ET1}CG15661[MB01781]
FBti0075413	23213 y[1] w[67c23]; Mi{ET1}MB01789
FBti0075414	23214 y[1] w[*]/Dp(1;Y)y[+]; Mi{ET1}slik[MB01795]
FBti0075415	23215 Mi{ET1}CG32816[MB01802] CG3777[MB01802] w[1118]
FBti0075416	23216 y[1] w[67c23]; Mi{ET1}CG14339[MB01805]
FBti0075417	23217 y[1] w[67c23]; Mi{ET1}CG31933[MB01818]
FBti0075418	23218 y[1] w[67c23]; Mi{ET1}MB01823
FBti0075419	23760 y[1] w[67c23]; Mi{ET1}MB01825

FBti0075420	23219 y[1] w[67c23]; Mi{ET1}MB01830
FBti0075421	23761 y[1] w[67c23]; Mi{ET1}Zasp52[MB01837]
FBti0075422	23220 y[1] w[67c23]; Mi{ET1}CG1213[MB01853]
FBti0075423	23762 y[1] w[67c23]; Mi{ET1}Syn[MB01857] Timp[MB01857]
FBti0075425	23221 y[1] w[67c23]; Mi{ET1}sha[MB01870]
FBti0075426	24452 y[1] w[67c23]; Mi{ET1}MB01877
FBti0075427	23763 w[1118] Mi{ET1}kek5[MB01879]
FBti0075428	23222 y[1] w[67c23]; Mi{ET1}CG14331[MB01897] CG5873[MB01897]
FBti0075429	23765 y[1] w[67c23]; Mi{ET1}MB01912
FBti0075430	23766 y[1] w[67c23]; Mi{ET1}Acp53C14a[MB01917]
FBti0075431	23223 y[1] w[67c23]; Mi{ET1}MB01920
FBti0075432	23224 y[1] w[67c23]; Mi{ET1}MB01939
FBti0075434	23226 y[1] w[67c23]; Mi{ET1}cv-c[MB01956]/TM3, Sb[1] Ser[1]
FBti0075465	23389 y[1] w[67c23]; Mi{ET1}CG32373[MB00260]
FBti0075466	23390 y[1] w[67c23]; Mi{ET1}Doa[MB00621]
FBti0075467	23391 y[1] w[67c23]; Mi{ET1}CG32111[MB00646]
FBti0075468	23392 y[1] w[67c23]; Mi{ET1}α-Est8[MB00671]
FBti0075469	23393 y[1] w[67c23]; Mi{ET1}amon[MB00756]
FBti0075470	23729 y[1] w[67c23]; Mi{ET1}bib[MB00798]
FBti0075471	23555 w[1118]; Mi{ET1}LanA[MB01129]/TM6C, Sb[1]
FBti0075473	23394 y[1]; Mi{ET1}Syt7[MB01453]
FBti0075475	23395 w[1118] Mi{ET1}CG34411[MB01485]
FBti0075476	23741 y[1] w[67c23]; Mi{ET1}MB01498
FBti0075477	23396 y[1]; Mi{ET1}CG32000[MB01559]
FBti0075478	24451 w[1118]/Dp(1;Y)y[+]; Mi{ET1}Osi17[MB01628]
FBti0075479	24450 y[1] w[1118]; Mi{ET1}Hcf[MB01666]
FBti0075480	23397 y[1] w[67c23]; Mi{ET1}unc79[MB01679]
FBti0075481	23398 w[1118] Mi{ET1}Cyp4s3[MB01696]
FBti0075483	23756 y[1] w[67c23]; Mi{ET1}CG12084[MB01742]
FBti0075484	23399 y[1] w[67c23]; Mi{ET1}CG17360[MB01767]/TM3, Sb[1] Ser[1]
FBti0075485	23400 y[1] w[67c23]; Mi{ET1}CG43980[MB01814]
FBti0075486	23401 y[1] w[67c23]; Mi{ET1}MB01850
FBti0075487	23764 y[1] w[67c23]; Mi{ET1}CG34242[MB01911]
FBti0075488	23402 y[1] w[67c23]; Mi{ET1}dpr4[MB01913]
FBti0075489	23403 y[1] w[67c23]; Mi{ET1}MB01958
FBti0075490	23767 w[1118]; Mi{ET1}CG13830[MB01961]
FBti0075491	23404 w[1118]; Mi{ET1}CG42260[MB01964]
FBti0075492	23465 w[1118]; Mi{ET1}CG4770[MB01974]/TM6C, Sb[1]
FBti0075493	23768 w[1118]; Mi{ET1}CG33116[MB01977]

FBti0075494	23405 w[1118]; Mi{ET1}CG11320[MB01978]
FBti0075495	23406 w[1118]; Mi{ET1}plx[MB01981]
FBti0075496	23556 w[1118]; Mi{ET1}CG6125[MB01987]
FBti0075497	23466 w[1118] Mi{ET1}CG8289[MB01992]
FBti0075498	23407 w[1118]; Mi{ET1}CG13071[MB01995]
FBti0075499	23557 w[1118]; Mi{ET1}CG6619[MB02006]
FBti0075500	23408 w[1118]; Mi{ET1}Sems[MB02013]
FBti0075501	23409 w[1118]; Mi{ET1}Rbp[MB02027]/TM6C, Sb[1]
FBti0075502	23410 w[1118]; Mi{ET1}CG11902[MB02045]
FBti0075503	23411 w[1118]; Mi{ET1}form3[MB02055]
FBti0075504	23412 w[1118]; Mi{ET1}CG17732[MB02058]
FBti0075505	23413 w[1118]; Mi{ET1}Dscam4[MB02068]
FBti0075506	23414 w[1118]; Mi{ET1}CG11426[MB02073]
FBti0075507	23558 w[1118]; Mi{ET1}MB02086
FBti0075508	23415 w[1118]; Mi{ET1}jdp[MB02104]
FBti0075509	23416 w[1118]; Mi{ET1}CG11000[MB02121]
FBti0075510	23417 w[1118]; Mi{ET1}GluRIB[MB02126]
FBti0075511	23418 w[1118]; Mi{ET1}UK114[MB02149]
FBti0075512	23419 w[1118]; Mi{ET1}CG30083[MB02150]
FBti0075513	23420 w[1118]; Mi{ET1}stumps[MB02154]
FBti0075514	23467 w[1118]; Mi{ET1}ush[MB02155]
FBti0075515	23468 w[1118]; Mi{ET1}CG15170[MB02157]
FBti0075516	23421 w[1118]; Mi{ET1}gprs[MB02158]
FBti0075517	23469 w[1118]; Mi{ET1}CG5104[MB02165] CG4825[MB02165]
FBti0075518	23559 w[1118]; Mi{ET1}Ugt[MB02166]/TM6C, Sb[1]
FBti0075519	23470 w[1118]; Mi{ET1}Piezo[MB02167]
FBti0075520	23422 w[1118]; Mi{ET1}CAH1[MB02177] adat[MB02177]
FBti0075521	23423 w[1118]; Mi{ET1}Cpr66D[MB02183]
FBti0075522	23471 w[1118]; Mi{ET1}Or85d[MB02185]
FBti0075523	23424 w[1118]; Mi{ET1}Ir94b[MB02190]
FBti0075524	23425 w[1118]; Mi{ET1}Drip[MB02194]
FBti0075525	23426 w[1118]; Mi{ET1}MB02227
FBti0075526	23769 w[1118]; Mi{ET1}CG9825[MB02228]
FBti0075527	23427 w[1118]; Mi{ET1}CG12943[MB02238]
FBti0075528	23428 w[1118]; Mi{ET1}TrissinR[MB02243]
FBti0075529	23429 w[1118]; Mi{ET1}CG43143[MB02272]
FBti0075530	23770 w[1118]; Mi{ET1}CG13594[MB02278]
FBti0075531	23430 w[1118]; Mi{ET1}SP1029[MB02279]
FBti0075532	23431 w[1118]; Mi{ET1}tkv[MB02285]

FBti0075533	23432 w[1118]; Mi{ET1}Msr-110[MB02290]
FBti0075534	23433 w[1118]; Mi{ET1}CG6197[MB02291]/SM6a
FBti0075535	23434 w[1118]; Mi{ET1}CG13086[MB02292]
FBti0075536	23472 w[1118]; Mi{ET1}CG6149[MB02293]
FBti0075537	23560 w[1118]; Mi{ET1}Corin[MB02295]
FBti0075538	23435 w[1118]; Mi{ET1}CG31148[MB02296]
FBti0075540	23473 w[1118]; Mi{ET1}Ir48b[MB02315]
FBti0075541	23437 w[1118]; Mi{ET1}MB02320
FBti0075542	24457 w[1118]; Mi{ET1}MB02326/CyO
FBti0075543	23438 w[1118]; Mi{ET1}MB02327
FBti0075544	23561 w[1118]; Mi{ET1}CG30271[MB02328]
FBti0075545	23439 w[1118]; Mi{ET1}MB02329
FBti0075546	23474 w[1118]; Mi{ET1}ACXD[MB02330]/TM6C, Sb[1]
FBti0075547	23440 w[1118]; Mi{ET1}MB02344
FBti0075548	23441 w[1118]; Mi{ET1}Dscam4[MB02353]
FBti0075549	23562 w[1118]; Mi{ET1}CG33339[MB02357]
FBti0075551	23563 w[1118]; Mi{ET1}MB02359 Mi{ET1}CG17150[MB02359]
FBti0075552	23564 w[1118]; Mi{ET1}MB02361
FBti0075553	23475 w[1118]; Mi{ET1}CG14855[MB02378]
FBti0075554	23442 w[1118]; Mi{ET1}VGlut[MB02384]
FBti0075555	23565 w[1118]; Mi{ET1}PIP5K59B[MB02388]
FBti0075556	23566 w[1118]; Mi{ET1}cp309[MB02408]
FBti0075557	23476 w[1118]; Mi{ET1}CG32052[MB02409]
FBti0075558	23567 w[1118]; Mi{ET1}sas[MB02437]
FBti0075559	23443 w[1118]; Mi{ET1}CG5421[MB02442]
FBti0075560	23568 w[1118]; Mi{ET1}Doc1[MB02443]/TM3, Sb[1] Ser[1]
FBti0075561	23444 w[1118]; Mi{ET1}MB02449
FBti0075562	23569 w[1118]; Mi{ET1}Pop2[MB02463]/TM6C, Sb[1]
FBti0075563	23477 w[1118]; Mi{ET1}CG30203[MB02464]/SM6a
FBti0075564	23445 w[1118] Mi{ET1}Cyp318a1[MB02480]
FBti0075566	23446 w[1118]; Mi{ET1}CG14830[MB02495]
FBti0075567	23447 w[1118]; Mi{ET1}CG12963[MB02497]
FBti0075568	23448 w[1118]; Mi{ET1}Rgk1[MB02498]
FBti0075569	23449 w[1118]; Mi{ET1}CG2082[MB02501]
FBti0075570	23571 w[1118]; Mi{ET1}GstZ1[MB02503]
FBti0075571	23771 w[1118]; Mi{ET1}MB02512
FBti0075573	23572 w[1118]; Mi{ET1}CG30447[MB02526]
FBti0075575	23479 w[1118]; Mi{ET1}CG12071[MB02534]
FBti0075576	23451 w[1118]; Mi{ET1}CG6138[MB02537] Trim9[MB02537]

FBti0075577	23452 w[1118]; Mi{ET1}MB02538
FBti0075578	23573 w[1118] Mi{ET1}CG3679[MB02546]
FBti0075579	23574 w[1118]; Mi{ET1}PK2-R1[MB02552]
FBti0075580	23575 w[1118]; Mi{ET1}Kaz1-ORFA[MB02559] Kaz1-ORFB[MB02559]
FBti0075581	23480 w[1118]; Mi{ET1}CG8552[MB02562]
FBti0075582	23576 w[1118]; Mi{ET1}sens[MB02566]
FBti0075583	23577 w[1118]; Mi{ET1}nyo[MB02571]
FBti0075584	23578 w[1118]; Mi{ET1}ItgαPS4[MB02574]
FBti0075585	23579 w[1118]; Mi{ET1}CG44153[MB02575]
FBti0075586	23580 w[1118]; Mi{ET1}CG13786[MB02584]
FBti0075587	23581 w[1118]; Mi{ET1}CG1311[MB02591]
FBti0075588	23582 w[1118]; Mi{ET1}CG15270[MB02602]
FBti0075589	23583 w[1118] Mi{ET1}NAAT1[MB02611]
FBti0075590	23584 w[1118]; Mi{ET1}CG32260[MB02623]
FBti0075591	23772 w[1118] Mi{ET1}MB02627
FBti0075592	23481 w[1118]; Mi{ET1}CG15695[MB02636]
FBti0075593	23453 w[1118]; Mi{ET1}prom[MB02637]
FBti0075595	23455 w[1118]; Mi{ET1}CG13618[MB02652]
FBti0075596	23456 w[1118]; Mi{ET1}MB02658
FBti0075597	23457 w[1118]; Mi{ET1}CG32032[MB02659]
FBti0075598	23458 w[1118]; Mi{ET1}GstD6[MB02663]
FBti0075599	23459 w[1118]; Mi{ET1}ninaC[MB02664]
FBti0075600	23460 w[1118]; Mi{ET1}CG15147[MB02665]
FBti0075601	23461 w[1118]; Mi{ET1}CG34362[MB02668]
FBti0075602	23462 w[1118]; Mi{ET1}MB02670
FBti0075603	23463 w[1118]; Mi{ET1}MB02678
FBti0075604	23464 w[1118] Mi{ET1}CG6617[MB02683] CG6540[MB02683]
FBti0075605	23482 w[1118] Mi{ET1}CCKLR-17D1[MB02688]
FBti0075606	23483 w[1118]; Mi{ET1}CG31413[MB02698]
FBti0075607	23585 w[1118]; Mi{ET1}Shab[MB02726]
FBti0075608	23484 w[1118]; Mi{ET1}Dat[MB02738]
FBti0075609	23485 w[1118]; Mi{ET1}CG10073[MB02747]
FBti0075610	23486 w[1118]; Mi{ET1}CG10710[MB02752]
FBti0075611	23487 w[1118]; Mi{ET1}emp[MB02756]
FBti0075612	23586 Mi{ET1}CG3777[MB02761] CG13375[MB02761] w[1118]
FBti0075613	23488 w[1118]; Mi{ET1}CG32373[MB02773] Ank2[MB02773]
FBti0075614	23489 w[1118]; Mi{ET1}MB02774
FBti0075615	23587 w[1118]; Mi{ET1}Cyp28d2[MB02776]/TM3, Sb[1] Ser[1]
FBti0075616	23490 w[1118]; Mi{ET1}zormin[MB02779]

FBti0075617	23491 w[1118]; Mi{ET1}hug[MB02782]
FBti0075618	23492 w[1118]; Mi{ET1}CG33281[MB02784]
FBti0075619	23493 w[1118]; Mi{ET1}MB02792
FBti0075620	23494 w[1118]; Mi{ET1}MB02795
FBti0075621	23495 w[1118]; Mi{ET1}dpr20[MB02798]
FBti0075623	23497 w[1118]; Mi{ET1}MB02803
FBti0075624	23498 w[1118]; Mi{ET1}CG4409[MB02805]
FBti0075625	23588 w[1118]; Mi{ET1}mRpL21[MB02827]/TM6C, Sb[1]
FBti0075626	23589 w[1118]; Mi{ET1}CG1443[MB02828]
FBti0075627	23590 w[1118]; Mi{ET1}MB02837
FBti0075628	23591 w[1118]; Mi{ET1}CG32150[MB02846]/TM3, Sb[1] Ser[1]
FBti0075629	23499 w[1118]; Mi{ET1}MB02849
FBti0075630	23592 w[1118]; Mi{ET1}MB02864/TM6C, Sb[1]
FBti0075631	23500 w[1118]; Mi{ET1}MB02908
FBti0075632	23501 w[1118]; Mi{ET1}Esyt2[MB02922]/TM6C, Sb[1]
FBti0075633	23593 w[1118]; Mi{ET1}α-Est6[MB02935]
FBti0075634	23594 w[1118]; Mi{ET1}dmrt93B[MB02937]
FBti0075635	23595 w[1118]; Mi{ET1}CG15545[MB02956]
FBti0075636	23502 w[1118]; Mi{ET1}CG5958[MB02978]
FBti0075637	23503 w[1118]; Mi{ET1}CG42362[MB02979]
FBti0075638	23504 w[1118]; Mi{ET1}dom[MB02981]
FBti0075639	23596 w[1118]; Mi{ET1}CG7579[MB02986]/TM3, Sb[1] Ser[1]
FBti0075640	23597 w[1118]; Mi{ET1}CG17770[MB02987]/TM6C, Sb[1]
FBti0075641	23505 w[1118]; Mi{ET1}Lim3[MB02989]
FBti0075642	23506 w[1118]; Mi{ET1}Ziz[MB02999]
FBti0075643	23598 w[1118]; Mi{ET1}Sb[MB03009]
FBti0075644	23599 w[1118]; Mi{ET1}MB03031
FBti0075645	23600 w[1118]; Mi{ET1}zye[MB03036]
FBti0075646	23601 w[1118]; Mi{ET1}fln[MB03038]/TM3, Sb[1] Ser[1]
FBti0075647	23602 w[1118]; Mi{ET1}CG31414[MB03039]/TM6C, Sb[1]
FBti0075648	23507 w[1118]; Mi{ET1}MB03040
FBti0075649	23508 w[1118]; Mi{ET1}CG31760[MB03046]
FBti0075650	23509 w[1118]; Mi{ET1}CR31824[MB03060]
FBti0075651	23510 w[1118]; Mi{ET1}dsf[MB03062]
FBti0075652	23511 w[1118]; Mi{ET1}ClC-a[MB03070]
FBti0075654	23513 w[1118]; Mi{ET1}CG31368[MB03076] mthl5[MB03076]
FBti0075656	23603 w[1118]; Mi{ET1}CG33203[MB03102]
FBti0075657	23604 w[1118]; Mi{ET1}CG18417[MB03109]
FBti0075658	23605 w[1118]; Mi{ET1}CG11843[MB03115]

FBti0075659	23606 w[1118]; Mi{ET1}MB03116
FBti0075660	23514 w[1118]; Mi{ET1}CG14183[MB03150]
FBti0075661	23515 w[1118]; Mi{ET1}Mst57Da[MB03151]
FBti0075662	23607 w[1118] Mi{ET1}MB03163
FBti0075663	23516 w[1118]; Mi{ET1}ACXE[MB03178]
FBti0075664	23517 w[1118]; Mi{ET1}Dh44-R1[MB03192]
FBti0075665	23518 w[1118]; Mi{ET1}bond[MB03193]
FBti0075666	23519 w[1118]; Mi{ET1}Gyc-89Db[MB03197]
FBti0075667	23520 w[1118]; Mi{ET1}glob3[MB03202]
FBti0075668	23521 w[1118]; Mi{ET1}CG43783[MB03223]
FBti0075669	23522 w[1118]; Mi{ET1}CG4230[MB03224]
FBti0075670	23523 w[1118]; Mi{ET1}CG32369[MB03233]/TM6C, Sb[1]
FBti0075671	23524 w[1118]; Mi{ET1}ash1[MB03235]/TM6C, Sb[1]
FBti0075672	23525 w[1118]; Mi{ET1}CG8552[MB03243]
FBti0075673	23526 w[1118]; Mi{ET1}MB03248
FBti0075674	23527 w[1118]; Mi{ET1}dpr12[MB03255]
FBti0075675	23528 w[1118]; Mi{ET1}Lasp[MB03281] CG9692[MB03281]
FBti0075676	23529 w[1118]; Mi{ET1}CG4995[MB03285]
FBti0075677	23530 w[1118]; Mi{ET1}Cyp28d1[MB03293]
FBti0075678	23531 w[1118]; Mi{ET1}CCHa2r[MB03316]
FBti0075679	23532 w[1118]; Mi{ET1}Or98b[MB03318]
FBti0075680	23533 w[1118]; Mi{ET1}MB03323
FBti0075681	23534 w[1118]; Mi{ET1}CG6472[MB03349] CG8910[MB03349]
FBti0075682	23535 w[1118]; Mi{ET1}CG44085[MB03357]
FBti0075683	23536 w[1118]; Mi{ET1}Nup54[MB03363]/SM6a
FBti0075684	23608 w[1118]; Mi{ET1}CG34139[MB03367]
FBti0075685	23537 w[1118]; Mi{ET1}MB03377
FBti0075686	23609 w[1118]; Mi{ET1}Fcp1[MB03378]/SM6a
FBti0075687	23610 w[1118]; Mi{ET1}MB03379
FBti0075688	23611 w[1118]; Mi{ET1}Dhc98D[MB03402]
FBti0075689	23612 w[1118]; Mi{ET1}CG9682[MB03410]
FBti0075690	23613 w[1118] Mi{ET1}MB03419
FBti0075691	23614 w[1118]; Mi{ET1}CG31637[MB03435]
FBti0075692	23615 w[1118] Mi{ET1}CG34411[MB03440]
FBti0075693	23616 w[1118]; Mi{ET1}MB03454
FBti0075694	23617 w[1118]; Mi{ET1}dpr5[MB03458]
FBti0075695	23538 w[1118]; Mi{ET1}CG9134[MB03465]
FBti0075696	23618 w[1118]; Mi{ET1}MB03472
FBti0075697	23619 w[1118]; Mi{ET1}CG13587[MB03476]

FBti0075698	23620 w[1118] Mi{ET1}CG15202[MB03501]
FBti0075699	23621 w[1118]; Mi{ET1}MB03504
FBti0075700	23622 w[1118]; Mi{ET1}Best4[MB03507]
FBti0075701	23623 w[1118]; Mi{ET1}Ih[MB03508]
FBti0075702	23624 w[1118]; Mi{ET1}CG44013[MB03512]
FBti0075703	23625 w[1118] Mi{ET1}CG42343[MB03513]
FBti0075704	23626 w[1118]; Mi{ET1}MB03528/TM3, Sb[1] Ser[1]
FBti0075705	23627 w[1118]; Mi{ET1}CDase[MB03531]/TM6C, Sb[1]
FBti0075706	23628 w[1118]; Mi{ET1}Gr64e[MB03533] Gr64d[MB03533]
FBti0075707	23539 w[1118]; Mi{ET1}CG14007[MB03535]
FBti0075708	23540 w[1118]; Mi{ET1}atilla[MB03539]
FBti0075709	23541 w[1118]; Mi{ET1}CG12766[MB03542]
FBti0075710	23629 w[1118]; Mi{ET1}CG4069[MB03548]
FBti0075711	23542 w[1118]; Mi{ET1}Atg4[MB03551]
FBti0075712	23630 w[1118]; Mi{ET1}Mst57Dc[MB03566]
FBti0075713	23543 w[1118]; Mi{ET1}pio[MB03570]
FBti0075714	23631 w[1118] Mi{ET1}Sdic4[MB03575]
FBti0075715	23632 w[1118]; Mi{ET1}nmo[MB03577] nmo[MB03577]/TM6C, Sb[1]
FBti0075716	23544 w[1118]; Mi{ET1}MB03583
FBti0075717	23545 w[1118]; Mi{ET1}Cpr31A[MB03597]
FBti0075718	23546 w[1118]; Mi{ET1}MB03602
FBti0075719	23633 w[1118] Mi{ET1}CG1889[MB03604]
FBti0075720	23547 w[1118]; Mi{ET1}MB03606
FBti0075721	23634 w[1118]; Mi{ET1}Rfx[MB03617]
FBti0075722	23548 w[1118]; Mi{ET1}CG31784[MB03624]
FBti0075724	23773 w[1118]; Mi{ET1}CG15260[MB03642]
FBti0075725	23549 w[1118]; Mi{ET1}CG15263[MB03647]
FBti0075726	23635 w[1118]; Mi{ET1}Sgs1[MB03659]
FBti0075727	23550 w[1118]; Mi{ET1}MB03663
FBti0075728	23636 w[1118]; Mi{ET1}trp[MB03672]
FBti0075729	23551 w[1118]; Mi{ET1}CG12484[MB03693]
FBti0075730	23552 w[1118] Mi{ET1}MB03696
FBti0075731	23553 w[1118]; Mi{ET1}CG7781[MB03700]
FBti0075732	23637 w[1118]; Mi{ET1}Sh3β[MB03704]
FBti0075733	23638 w[1118]; Mi{ET1}Ir92a[MB03705]
FBti0075734	23554 w[1118]; Mi{ET1}GABA-B-R3[MB03707]
FBti0075736	22749 y[1] w[67c23]; Mi{ET1}sif[MB00266]
FBti0075737	22752 y[1] w[67c23]; Mi{ET1}MB00275
FBti0075738	22753 y[1] w[67c23]; Mi{ET1}MB00276

FBti0075739	22754 y[1] w[67c23]; Mi{ET1}caup[MB00278]
FBti0075740	22755 y[1] w[67c23]; Mi{ET1}MB00288
FBti0075741	22756 y[1] w[67c23]; Mi{ET1}CG31323[MB00294]
FBti0075742	22757 y[1] w[67c23]; Mi{ET1}MB00295/TM3, Sb[1] Ser[1]
FBti0075743	22758 y[1] w[67c23]; Mi{ET1}Oamb[MB00297]
FBti0075744	22759 Mi{ET1}CG15890[MB00299] w[1118]
FBti0075746	22762 w[1118] Mi{ET1}Pkcδ[MB00303]
FBti0075747	22763 y[1] w[67c23]; Mi{ET1}CG9316[MB00308]
FBti0075748	22764 y[1] w[67c23]; Mi{ET1}MB00317
FBti0075749	22765 y[1] w[67c23]; Mi{ET1}MB00318
FBti0075750	22766 y[1] w[67c23]; Mi{ET1}MB00319
FBti0075751	22767 y[1] w[67c23]; Mi{ET1}CG10252[MB00324]
FBti0075752	22768 y[1] w[67c23]; Mi{ET1}MB00331
FBti0075753	22769 y[1] w[67c23]; Mi{ET1}CG32944[MB00336]
FBti0075754	22783 y[1] w[67c23]; Mi{ET1}Lnk[MB00388]
FBti0075755	22810 y[1] w[67c23]; Mi{ET1}CG4341[MB00473]
FBti0075756	22822 Mi{ET1}MB00522 w[1118]
FBti0075759	22828 y[1] w[67c23]; Mi{ET1}MB00530
FBti0075761	22830 w[1118] Mi{ET1}MB00532
FBti0075763	22845 y[1] w[67c23]; Mi{ET1}CG15629[MB00580]
FBti0075764	22851 y[1] w[67c23]; Mi{ET1}CG11873[MB00611]/TM3, Sb[1] Ser[1]
FBti0075765	22852 y[1] w[67c23]; Mi{ET1}jar[MB00612]/TM3, Sb[1] Ser[1]
FBti0075766	22862 w[1118] Mi{ET1}rad[MB00656]
FBti0075767	22864 w[1118] Mi{ET1}MB00664
FBti0075768	22873 y[1] w[67c23]; Mi{ET1}CG44837[MB00693]
FBti0075769	22876 y[1] w[67c23]; Mi{ET1}egg[MB00702]/CyO
FBti0075770	22878 y[1] w[67c23]; Mi{ET1}CG5142[MB00716]
FBti0075771	22880 y[1] w[67c23]; Mi{ET1}lobo[MB00722]
FBti0075772	22882 y[1] w[67c23]; Mi{ET1}CG31128[MB00728]
FBti0075773	22885 y[1] w[67c23]; Mi{ET1}MB00738
FBti0075775	22923 y[1] w[67c23]; Mi{ET1}MB00884
FBti0075776	22934 y[1] w[67c23]; Mi{ET1}CG3984[MB00918]
FBti0075777	22936 y[1] w[67c23]; Mi{ET1}MB00926
FBti0075778	22937 y[1] w[67c23]; Mi{ET1}CG7341[MB00927]
FBti0075779	22939 y[1] w[67c23]; Mi{ET1}MB00949
FBti0075780	22942 y[1] w[67c23]; Mi{ET1}MB00957
FBti0075781	22947 y[1] w[67c23]; Mi{ET1}MB00971
FBti0075782	22948 y[1] w[67c23]; Mi{ET1}MB00975
FBti0075783	22949 y[1] w[67c23]; Mi{ET1}Hsrω[MB00978]

FBti0075784	22951 y[1] w[67c23]; Mi{ET1}CG13708[MB00980]
FBti0075785	22952 y[1] w[67c23]; Mi{ET1}CG10170[MB00984]
FBti0075786	22954 y[1] w[67c23]; Mi{ET1}MB00986
FBti0075787	22955 w[1118] Mi{ET1}kirre[MB00990]
FBti0075788	22956 w[1118] Mi{ET1}CG34104[MB00991]
FBti0075789	22957 y[1] w[67c23]; Mi{ET1}MB00993
FBti0075790	22960 y[1] w[67c23]; Mi{ET1}tinc[MB01004]
FBti0075791	22961 w[1118] Mi{ET1}MB01008
FBti0075792	22962 y[1] w[67c23]; Mi{ET1}CG3822[MB01010]
FBti0075793	22964 y[1] w[67c23]; Mi{ET1}MB01015
FBti0075794	22966 y[1] w[67c23]; Mi{ET1}MB01018
FBti0075795	22968 y[1] w[67c23]; Mi{ET1}MB01021
FBti0075796	22970 w[1118] Mi{ET1}bves[MB01031]
FBti0075797	22972 y[1] w[67c23]; Mi{ET1}p130CAS[MB01039]a
FBti0075799	22974 y[1] w[67c23]; Mi{ET1}CG7408[MB01042]
FBti0075800	22975 y[1] w[67c23]; Mi{ET1}CG42260[MB01043]
FBti0075801	22976 y[1] w[67c23]; Mi{ET1}LpR1[MB01048]
FBti0075803	22979 y[1] w[67c23]; Mi{ET1}MB01060
FBti0075804	22981 y[1] w[67c23]; Mi{ET1}MB01064
FBti0075805	22982 y[1] w[67c23]; Mi{ET1}MB01065
FBti0075806	22985 y[1] w[67c23]; Mi{ET1}CG13857[MB01078]
FBti0075807	22986 y[1] w[67c23]; Mi{ET1}CG3940[MB01088]
FBti0075808	22989 y[1] w[67c23]; Mi{ET1}heph[MB01096]
FBti0075809	22990 y[1] w[67c23]; Mi{ET1}CG7557[MB01098]
FBti0075810	22993 y[1] w[67c23]; Mi{ET1}mp[MB01109]
FBti0075811	22996 y[1] w[67c23]; Mi{ET1}CG32113[MB01121]
FBti0075812	23000 y[1] w[67c23]; Mi{ET1}comm2[MB01146]
FBti0075813	23004 y[1] w[67c23]; Mi{ET1}pnt[MB01157]
FBti0075814	23005 y[1] w[67c23]; Mi{ET1}CG8320[MB01163] ATPCL[MB01163]
FBti0075815	23006 y[1] w[67c23]; Mi{ET1}CS-2[MB01164]
FBti0075816	23011 y[1] w[67c23]; Mi{ET1}uzip[MB01177]
FBti0075817	23013 y[1] w[67c23]; Mi{ET1}CG32432[MB01182]
FBti0075818	23018 y[1] w[67c23]; Mi{ET1}Hs3st-A[MB01199]
FBti0075819	23020 y[1] w[67c23]; Mi{ET1}Vmat[MB01214]
FBti0075820	23027 y[1] w[67c23]; Mi{ET1}MB01231
FBti0075821	23035 w[1118] Mi{ET1}CG32791[MB01249]
FBti0075822	23038 y[1] w[67c23]; Mi{ET1}MB01263
FBti0075823	23048 y[1] w[67c23]; Mi{ET1}CG3746[MB01292]
FBti0075825	23062 y[1] w[67c23]; Mi{ET1}CG16947[MB01338]

FBti0075826	23073 y[1] w[67c23]; Mi{ET1}MB01357
FBti0075827	23085 y[1] w[67c23]; Mi{ET1}MB01388
FBti0075828	22661 y[1]; Mi{ET1}CG9935[MB00001]
FBti0075829	22718 y[1] w[67c23]; Mi{ET1}Dh31-R[MB00175]
FBti0076162	22663 y[1] w[67c23]; Mi{ET1}MB00003/TM3, Sb[1] Ser[1]
FBti0076163	22664 y[1] w[67c23]; Mi{ET1}kkv[MB00004]
FBti0076164	22665 y[1] w[67c23]; Mi{ET1}CG32373[MB00005] Ank2[MB00005]
FBti0076166	22667 y[1] w[67c23]; Mi{ET1}Ac78C[MB00011]
FBti0076167	22668 y[1] w[67c23]; Mi{ET1}MB00015/TM3, Sb[1] Ser[1]
FBti0076168	22669 y[1] w[67c23]; Mi{ET1}luna[MB00021]
FBti0076169	22670 y[1] w[67c23]; Mi{ET1}CG7497[MB00023]
FBti0076170	22671 y[1] w[67c23]; Mi{ET1}nord[MB00024]
FBti0076172	22673 y[1] w[67c23]; Mi{ET1}MB00026
FBti0076173	22675 y[1] w[67c23]; Mi{ET1}MB00028
FBti0076174	22676 y[1] w[67c23]; Mi{ET1}MB00032
FBti0076175	22677 y[1] w[67c23]; Mi{ET1}MB00035
FBti0076176	22678 y[1] w[67c23]; Mi{ET1}MB00037
FBti0076177	22679 y[1] w[67c23]; Mi{ET1}CG42266[MB00038]
FBti0076178	22680 y[1] w[67c23]; Mi{ET1}CG32206[MB00039]
FBti0076179	22681 y[1] w[67c23]; Mi{ET1}CG5191[MB00040]
FBti0076180	22682 y[1] w[67c23]; Mi{ET1}MB00041
FBti0076181	22683 w[1118] Mi{ET1}mamo[MB00043]
FBti0076182	22684 y[1] w[67c23]; Mi{ET1}MB00045
FBti0076183	22685 y[1] w[67c23]; Mi{ET1}MB00051
FBti0076184	22686 y[1] w[67c23]; Mi{ET1}MB00056
FBti0076185	22687 y[1] w[67c23]; Mi{ET1}dyl[MB00062]
FBti0076186	22688 y[1] w[67c23]; Mi{ET1}CG31300[MB00063]
FBti0076187	22689 y[1] w[67c23]; Mi{ET1}Gfrl[MB00064]
FBti0076188	22690 y[1] w[67c23]; Mi{ET1}MB00070
FBti0076189	22691 y[1] w[67c23]; Mi{ET1}CG14238[MB00073]
FBti0076190	22692 y[1] w[67c23]; Mi{ET1}MB00074
FBti0076191	22693 y[1] w[67c23]; Mi{ET1}MB00076
FBti0076192	22695 y[1] w[67c23]; Mi{ET1}MB00080
FBti0076194	22697 y[1] w[67c23]; Mi{ET1}wry[MB00103]
FBti0076195	22698 y[1] w[67c23]; Mi{ET1}MB00108
FBti0076196	22699 y[1] w[67c23]; Mi{ET1}SKIP[MB00109]
FBti0076197	22700 y[1] w[67c23]; Mi{ET1}CG32176[MB00110] CG12229[MB00110]
FBti0076198	22701 y[1] w[67c23]; Mi{ET1}Poxn[MB00113]
FBti0076199	22702 y[1] w[67c23]; Mi{ET1}MB00120

FBti0076200	22703 y[1] w[67c23]; Mi{ET1}CG34370[MB00122]
FBti0076201	22704 y[1] w[67c23]; Mi{ET1}MB00124
FBti0076202	22705 y[1] w[67c23]; Mi{ET1}MB00129
FBti0076203	22706 y[1] w[67c23]; Mi{ET1}CG6337[MB00130]
FBti0076204	22707 y[1] w[67c23]; Mi{ET1}MB00134
FBti0076205	22708 y[1] w[67c23]; Mi{ET1}MB00135
FBti0076206	22709 y[1] w[67c23]; Mi{ET1}hebe[MB00136]
FBti0076207	22710 y[1] w[67c23]; Mi{ET1}MB00145
FBti0076208	22711 y[1] w[67c23]; Mi{ET1}gk[MB00149]
FBti0076209	22712 y[1] w[67c23]; Mi{ET1}Cbp53E[MB00150]
FBti0076210	22713 y[1] w[67c23]; Mi{ET1}CG15822[MB00151]
FBti0076211	22714 y[1] w[67c23]; Mi{ET1}MB00163
FBti0076212	22715 y[1] w[67c23]; Mi{ET1}MB00167
FBti0076213	22716 y[1] w[67c23]; Mi{ET1}MB00169
FBti0076214	22717 w[1118] Mi{ET1}MB00172
FBti0076215	22719 y[1] w[67c23]; Mi{ET1}MB00176
FBti0076216	22720 y[1] w[67c23]; Mi{ET1}CG9265[MB00177]/CyO
FBti0076217	22721 y[1] w[67c23]; Mi{ET1}CG4576[MB00179] rec[MB00179]
FBti0076219	22723 y[1] w[67c23]; Mi{ET1}Con[MB00185]
FBti0076220	22724 y[1] w[67c23]; Mi{ET1}CG5888[MB00188]
FBti0076221	22725 y[1] w[67c23]; Mi{ET1}CG11321[MB00197]
FBti0076222	22726 y[1] w[67c23]; Mi{ET1}MB00199
FBti0076223	22727 y[1] w[67c23]; Mi{ET1}Jon65Aii[MB00207]
FBti0076224	22728 y[1] w[67c23]; Mi{ET1}Dscam3[MB00214]
FBti0076225	22729 y[1] w[67c23]; Mi{ET1}Ir76b[MB00216]
FBti0076226	22730 y[1] w[67c23]; Mi{ET1}MB00219
FBti0076227	22731 y[1] w[67c23]; Mi{ET1}MB00220
FBti0076228	22732 y[1] w[67c23]; Mi{ET1}Rbp6[MB00221]
FBti0076229	22733 y[1] w[67c23]; Mi{ET1}Cht7[MB00223]
FBti0076230	22734 y[1] w[67c23]; Mi{ET1}CG9733[MB00227]
FBti0076231	22736 y[1] w[67c23]; Mi{ET1}MB00231
FBti0076232	22737 y[1] w[67c23]; Mi{ET1}CG13699[MB00234]
FBti0076233	22738 y[1] w[67c23]; Mi{ET1}Sema-1a[MB00238]
FBti0076234	22739 y[1] w[67c23]; Mi{ET1}MB00240
FBti0076235	22740 y[1] w[67c23]; Mi{ET1}MB00241/CyO
FBti0076236	22741 y[1] w[67c23]; Mi{ET1}CG34384[MB00245]
FBti0076237	22742 y[1] w[67c23]; Mi{ET1}MB00246
FBti0076238	22743 y[1] w[67c23]; Mi{ET1}MB00247
FBti0076239	22744 y[1] w[67c23]; Mi{ET1}ome[MB00248]

FBti0076240	22771 y[1] w[67c23]; Mi{ET1}MB00350
FBti0076241	22772 y[1] w[67c23]; Mi{ET1}Syn[MB00351]
FBti0076242	22773 y[1] w[67c23]; Mi{ET1}Mipp1[MB00354]
FBti0076243	22774 y[1] w[67c23]; Mi{ET1}DCX-EMAP[MB00356]
FBti0076244	22775 y[1] w[67c23]; Mi{ET1}CG11893[MB00360]
FBti0076245	22776 y[1] w[67c23]; Mi{ET1}CG43078[MB00361]
FBti0076246	22777 y[1] w[67c23]; Mi{ET1}CG34398[MB00367]
FBti0076247	22778 w[1118] Mi{ET1}MB00368
FBti0076248	22779 y[1] w[67c23]; Mi{ET1}MB00372
FBti0076249	22780 y[1] w[67c23]; Mi{ET1}CG44153[MB00374]
FBti0076250	22781 y[1] w[67c23]; Mi{ET1}Pif1A[MB00375]
FBti0076251	22782 y[1] w[67c23]; Mi{ET1}Pde1c[MB00379]
FBti0076252	22784 y[1] w[67c23]; Mi{ET1}CG6701[MB00389]
FBti0076253	22788 y[1] w[67c23]; Mi{ET1}MB00399
FBti0076254	22790 y[1] w[67c23]; Mi{ET1}MB00404
FBti0076255	22792 y[1] w[67c23]; Mi{ET1}Msp-300[MB00410]
FBti0076256	22795 y[1] w[67c23]; Mi{ET1}CG42260[MB00428]
FBti0076257	22798 w[1118] Mi{ET1}CG12535[MB00435]
FBti0076258	22803 w[1118] Mi{ET1}MB00450
FBti0076259	22817 y[1] w[67c23]; Mi{ET1}dpr3[MB00501]
FBti0076261	22819 y[1] w[67c23]; Mi{ET1}zormin[MB00505]
FBti0076263	22821 y[1] w[67c23]; Mi{ET1}Cpr97Eb[MB00515]
FBti0076264	22823 y[1] w[67c23]; Mi{ET1}MB00524
FBti0076265	22826 y[1] w[67c23]; Mi{ET1}MB00528
FBti0076266	22831 y[1] w[67c23]; Mi{ET1}CG34461[MB00536]
FBti0076267	22833 w[1118] Mi{ET1}DIP1[MB00541]
FBti0076268	22835 y[1] w[67c23]; Mi{ET1}CG15873[MB00550]
FBti0076269	22836 y[1] w[67c23]; Mi{ET1}MB00556
FBti0076270	22837 w[1118] Mi{ET1}Sh[MB00560]
FBti0076271	22838 y[1] w[67c23]; Mi{ET1}haf[MB00562] CG31935[MB00562]
FBti0076272	22839 y[1] w[67c23]; Mi{ET1}CG9967[MB00566]
FBti0076273	22840 y[1] w[67c23]; Mi{ET1}MB00567
FBti0076274	22841 w[1118] Mi{ET1}NetA[MB00571]
FBti0076275	22842 y[1] w[67c23]; Mi{ET1}MB00573
FBti0076276	22843 y[1] w[67c23]; Mi{ET1}MB00577
FBti0076277	22846 y[1] w[67c23]; Mi{ET1}CG16857[MB00585]
FBti0076278	22848 y[1] w[67c23]; Mi{ET1}CG14752[MB00604]
FBti0076279	22849 y[1] w[67c23]; Mi{ET1}SP2353[MB00605]
FBti0076280	22856 y[1] w[67c23]; Mi{ET1}MB00632

FBti0076281	22859 y[1] w[67c23]; Mi{ET1}MB00649
FBti0076282	22860 y[1] w[67c23]; Mi{ET1}Ptp61F[MB00652]
FBti0076283	22863 w[1118] Mi{ET1}MB00659
FBti0076284	22866 y[1] w[67c23]; Mi{ET1}Unc-89[MB00669]
FBti0076285	22869 y[1] w[67c23]; Mi{ET1}CG17839[MB00682]
FBti0076286	22870 y[1] w[67c23]; Mi{ET1}MB00686
FBti0076287	22871 y[1] w[67c23]; Mi{ET1}MB00690
FBti0076288	22872 y[1] w[67c23]; Mi{ET1}CG2812[MB00692]
FBti0076289	22877 y[1] w[67c23]; Mi{ET1}sano[MB00708]
FBti0076290	22879 y[1] w[67c23]; Mi{ET1}MB00719
FBti0076291	22932 y[1] w[67c23]; Mi{ET1}MB00913
FBti0076292	22933 y[1] w[67c23]; Mi{ET1}CG6293[MB00915]
FBti0076293	22938 y[1] w[67c23]; Mi{ET1}CG6972[MB00932]
FBti0076294	22940 y[1] w[67c23]; Mi{ET1}dally[MB00950]/TM6C, cu[1] Sb[1]
FBti0076295	22941 y[1] w[67c23]; Mi{ET1}CG13492[MB00954]
FBti0076296	22943 y[1] w[67c23]; Mi{ET1}CG18095[MB00959]
FBti0076297	22969 w[1118] Mi{ET1}MB01026
FBti0076298	22971 w[1118] Mi{ET1}MB01038
FBti0076299	22978 w[1118] Mi{ET1}RunxB[MB01057]
FBti0076300	22987 Mi{ET1}a6[MB01090] w[1118]
FBti0076302	23007 y[1] w[67c23]; Mi{ET1}Hr38[MB01166]
FBti0076303	23012 w[67c23] Mi{ET1}MB01180
FBti0076304	23023 y[1] w[67c23]; Mi{ET1}Dys[MB01222]
FBti0076305	23025 w[1118] Mi{ET1}MB01224
FBti0076306	23026 w[1118] Mi{ET1}CG32532[MB01230]
FBti0076307	23028 y[1] w[67c23]; Mi{ET1}CG7804[MB01233]
FBti0076308	23029 y[1] w[67c23]; Mi{ET1}Slob[MB01237]
FBti0076309	23031 y[1] w[67c23]; Mi{ET1}CG6282[MB01241]
FBti0076310	23033 y[1] w[67c23]; Mi{ET1}CG13894[MB01245]
FBti0076311	23034 y[1] w[67c23]; Mi{ET1}CG16791[MB01246]
FBti0076312	23041 w[1118] Mi{ET1}MB01268
FBti0076313	23042 y[1] w[67c23]; Mi{ET1}MB01274
FBti0076314	23043 y[1] w[67c23]; Mi{ET1}sqa[MB01276]
FBti0076315	23044 y[1] w[67c23]; Mi{ET1}CG9932[MB01281]
FBti0076316	23047 y[1] w[67c23]; Mi{ET1}MB01288
FBti0076317	23049 y[1] w[67c23]; Mi{ET1}CG33143[MB01293]/CyO
FBti0076318	23051 y[1] w[67c23]; Mi{ET1}CG5888[MB01297]
FBti0076319	23052 y[1] w[67c23]; Mi{ET1}gwl[MB01301]
FBti0076320	23070 y[1] w[67c23]; Mi{ET1}MB01351

FBti0076321	23071 y[1] w[67c23]; Mi{ET1}ppk20[MB01352]
FBti0076322	23075 y[1] w[67c23]; Mi{ET1}CG7059[MB01361]
FBti0076323	23076 Mi{ET1}MB01363 w[1118]
FBti0076324	23078 y[1] w[67c23]; Mi{ET1}MB01369
FBti0076326	23092 y[1] w[67c23]; Mi{ET1}RhoGAP100F[MB90151]
FBti0076327	22745 y[1] w[67c23]; Mi{ET1}Ir75a[MB00253]
FBti0076328	22746 y[1] w[67c23]; Mi{ET1}MB00259
FBti0076330	22750 y[1] w[67c23]; Mi{ET1}cpx[MB00267]
FBti0076331	22751 y[1] w[67c23]; Mi{ET1}UbcD6[MB00268]
FBti0076332	22760 y[1] w[67c23]; Mi{ET1}MB00301
FBti0076333	22770 y[1] w[67c23]; Mi{ET1}CG15888[MB00348]
FBti0076334	22785 y[1] w[67c23]; Mi{ET1}MB00393
FBti0076335	22786 y[1] w[67c23]; Mi{ET1}Shaw[MB00394]
FBti0076336	22787 y[1] w[67c23]; Mi{ET1}Myo95E[MB00398]
FBti0076338	22793 y[1] w[67c23]; Mi{ET1}bs[MB00417]
FBti0076339	22797 y[1] w[67c23]; Mi{ET1}bru-2[MB00431]
FBti0076340	22799 y[1] w[67c23]; Mi{ET1}Liprin-γ[MB00440]
FBti0076341	22800 y[1] w[67c23]; Mi{ET1}CREG[MB00443]
FBti0076342	22801 y[1] w[67c23]; Mi{ET1}Sobp[MB00447]
FBti0076343	22802 y[1] w[67c23]; Mi{ET1}CG13385[MB00448]
FBti0076344	22804 y[1] w[67c23]; Mi{ET1}dp[MB00453]
FBti0076345	22806 y[1] w[67c23]; Mi{ET1}CG32264[MB00461]
FBti0076346	22807 y[1] w[67c23]; Mi{ET1}CG32106[MB00463]
FBti0076347	22808 y[1] w[67c23]; Mi{ET1}MB00465
FBti0076348	22811 y[1] w[67c23]; Mi{ET1}MB00475
FBti0076349	22814 y[1] w[67c23]; Mi{ET1}sli[MB00487]
FBti0076350	22816 y[1] w[67c23]; Mi{ET1}robo3[MB00495]
FBti0076351	22832 y[1] w[67c23]; Mi{ET1}Tequila[MB00537]
FBti0076352	22858 y[1] w[67c23]; Mi{ET1}MB00642
FBti0076353	22875 y[1] w[67c23]; Mi{ET1}CG31176[MB00700]
FBti0076354	22881 y[1] w[67c23]; Mi{ET1}MB00725
FBti0076355	22883 y[1] w[67c23]; Mi{ET1}CG42321[MB00733]
FBti0076356	22886 y[1] w[67c23]; Mi{ET1}CG34371[MB00740]
FBti0076357	22892 y[1] w[67c23]; Mi{ET1}Tsp29Fb[MB00768]
FBti0076358	22895 w[1118] Mi{ET1}IP3K2[MB00775]
FBti0076359	22897 y[1] w[67c23]; Mi{ET1}CG8858[MB00783]
FBti0076360	22902 y[1] w[67c23]; Mi{ET1}MB00805
FBti0076361	22903 y[1] w[67c23]; Mi{ET1}CG43795[MB00810]
FBti0076362	22906 y[1] w[67c23]; Mi{ET1}RIC-3[MB00827]

FBti0076363	22909 y[1] w[67c23]; Mi{ET1}MB00836
FBti0076364	22911 y[1] w[67c23]; Mi{ET1}Cpr50Ca[MB00839]
FBti0076365	22915 y[1] w[67c23]; Mi{ET1}CG8550[MB00850]
FBti0076366	22916 y[1] w[67c23]; Mi{ET1}CG9463[MB00852]
FBti0076367	22919 y[1] w[67c23]; Mi{ET1}CG4691[MB00873]
FBti0076368	22920 y[1] w[67c23]; Mi{ET1}CG34113[MB00875]
FBti0076369	22922 w[1118] Mi{ET1}CG2233[MB00881]
FBti0076370	22924 y[1] w[67c23]; Mi{ET1}Gαo[MB00893]
FBti0076371	22926 y[1] w[67c23]; Mi{ET1}MB00896
FBti0076372	22927 y[1] w[67c23]; Mi{ET1}sowah[MB00901]
FBti0076373	22928 y[1] w[67c23]; Mi{ET1}Lapsyn[MB00906]
FBti0076375	22930 w[1118] Mi{ET1}Proc-R[MB00909]
FBti0076376	22935 Mi{ET1}msta[MB00924] w[1118]
FBti0076377	22944 w[1118] Mi{ET1}CG42343[MB00961]
FBti0076378	22945 y[1] w[67c23]; Mi{ET1}CG8740[MB00964]
FBti0076379	22946 y[1] w[67c23]; Mi{ET1}unc-5[MB00969]
FBti0076380	22950 y[1] w[67c23]; Mi{ET1}MB00979
FBti0076381	22958 y[1] w[67c23]; Mi{ET1}Jarid2[MB00996]
FBti0076382	22959 w[1118] Mi{ET1}AlstR[MB01003]
FBti0076383	22965 y[1] w[67c23]; Mi{ET1}ImpE1[MB01017]
FBti0076384	22967 y[1] w[67c23]; Mi{ET1}gogo[MB01020]
FBti0076385	22973 y[1] w[67c23]; Mi{ET1}bab1[MB01041]
FBti0076386	22983 y[1] w[67c23]; Mi{ET1}MB01072
FBti0076387	22991 y[1] w[67c23]; Mi{ET1}CG7510[MB01099]
FBti0076388	22994 w[1118] Mi{ET1}rst[MB01115]
FBti0076389	22995 y[1] w[67c23]; Mi{ET1}MB01119
FBti0076390	22997 y[1] w[67c23]; Mi{ET1}Cad74A[MB01124]
FBti0076391	22998 y[1] w[67c23]; Mi{ET1}CG43163[MB01128]
FBti0076392	23003 y[1] w[67c23]; Mi{ET1}CG10418[MB01154]
FBti0076393	23010 y[1] w[67c23]; Mi{ET1}MB01175
FBti0076394	23016 y[1] w[67c23]; Mi{ET1}MB01191
FBti0076395	23024 w[1118] Mi{ET1}CG3106[MB01223]
FBti0076396	23030 y[1] w[67c23]; Mi{ET1}CG1607[MB01239]
FBti0076397	23032 w[1118] Mi{ET1}Pvf1[MB01242]
FBti0076398	23036 y[1] w[67c23]; Mi{ET1}Cad86C[MB01251]
FBti0076399	23037 y[1] w[67c23]; Mi{ET1}tsl[MB01255]
FBti0076400	23039 y[1] w[67c23]; Mi{ET1}Sodh-2[MB01265]
FBti0076401	23046 y[1] w[67c23]; Mi{ET1}5PtaseI[MB01285]
FBti0076402	23050 y[1] w[67c23]; Mi{ET1}MB01295

FBti0076403	23053 y[1] w[67c23]; Mi{ET1}Zasp67[MB01313]
FBti0076404	23054 y[1] w[67c23]; Mi{ET1}MB01314
FBti0076405	23060 y[1] w[67c23]; Mi{ET1}CadN[MB01331]
FBti0076406	23065 y[1] w[67c23]; Mi{ET1}MB01343
FBti0076407	23067 y[1] w[67c23]; Mi{ET1}MB01345
FBti0076408	23074 y[1] w[67c23]; Mi{ET1}CG42750[MB01359]
FBti0076409	23081 y[1] w[67c23]; Mi{ET1}Obp49a[MB01375] nemy[MB01375]
FBti0076410	23084 Mi{ET1}MB01385 w[1118]
FBti0076411	23089 y[1] w[67c23]; Mi{ET1}MB01734
FBti0076412	23478 w[1118]; Mi{ET1}MB02529
FBti0077537	23859 w[1118]; Mi{ET1}bab1[MB01972]
FBti0077538	23775 w[1118]; Mi{ET1}lilli[MB01976]
FBti0077539	23776 w[1118]; Mi{ET1}CG5892[MB01984]
FBti0077540	23777 w[1118]; Mi{ET1}fru[MB01996]
FBti0077541	23778 w[1118]; Mi{ET1}beat-IIIc[MB01999]
FBti0077542	23779 w[1118]; Mi{ET1}CG13921[MB02003]
FBti0077543	23780 w[1118]; Mi{ET1}Sytβ[MB02011]
FBti0077544	23781 w[1118]; Mi{ET1}ppk11[MB02012]
FBti0077545	23782 w[1118]; Mi{ET1}CG30265[MB02029]
FBti0077546	23783 w[1118]; Mi{ET1}MB02034
FBti0077547	23784 w[1118]; Mi{ET1}Cda5[MB02050]
FBti0077548	23785 w[1118]; Mi{ET1}MB02060
FBti0077549	23786 w[1118]; Mi{ET1}MB02080
FBti0077550	23787 w[1118] Mi{ET1}CG12531[MB02081]
FBti0077551	23788 w[1118]; Mi{ET1}rgn[MB02115] sa[MB02115]
FBti0077552	23789 w[1118]; Mi{ET1}MB02148
FBti0077553	23790 w[1118]; Mi{ET1}MB02200
FBti0077554	23791 w[1118]; Mi{ET1}CG1688[MB02215]
FBti0077555	23792 w[1118] Mi{ET1}CG2258[MB02229]
FBti0077556	23793 w[1118]; Mi{ET1}MB02241
FBti0077557	23794 w[1118]; Mi{ET1}Letm1[MB02246]/SM6a
FBti0077558	23795 w[1118]; Mi{ET1}sprt[MB02257]
FBti0077559	23796 w[1118]; Mi{ET1}Fas3[MB02284]
FBti0077560	23797 w[1118]; Mi{ET1}MB02286
FBti0077561	23798 w[1118]; Mi{ET1}klar[MB02303]
FBti0077562	23799 w[1118]; Mi{ET1}Pde1c[MB02304]
FBti0077563	23800 w[1118]; Mi{ET1}MB02307
FBti0077564	23801 w[1118]; Mi{ET1}CG11261[MB02313]
FBti0077565	23802 y[1]; Mi{ET1}MB02338

FBti0077566	23803 w[1118]; Mi{ET1}Sesn[MB02362]
FBti0077567	23804 w[1118]; Mi{ET1}MB02363
FBti0077568	24794 w[1118] Mi{ET1}drd[MB02377]
FBti0077569	23805 w[1118]; Mi{ET1}sbb[MB02419]
FBti0077570	23806 w[1118]; Mi{ET1}MB02441
FBti0077571	23807 w[1118]; Mi{ET1}MB02455
FBti0077572	23808 w[1118] Mi{ET1}MB02467
FBti0077573	23809 w[1118]; Mi{ET1}MB02471
FBti0077574	23810 w[1118]; Mi{ET1}MB02474
FBti0077575	23811 w[1118]; Mi{ET1}ths[MB02475]
FBti0077576	23812 w[1118]; Mi{ET1}CG6006[MB02490]
FBti0077577	23813 w[1118]; Mi{ET1}MB02500
FBti0077578	23814 w[1118]; Mi{ET1}MB02506
FBti0077579	23815 w[1118]; Mi{ET1}MB02507
FBti0077580	23816 Mi{ET1}CG14785[MB02516], w[1118]
FBti0077581	23817 w[1118]; Mi{ET1}MB02527
FBti0077583	23818 w[1118]; Mi{ET1}MB02554
FBti0077584	23819 w[1118]; Mi{ET1}MB02588
FBti0077585	23820 w[1118]; Mi{ET1}MB02617
FBti0077588	23822 w[1118]; Mi{ET1}MB02673
FBti0077589	23823 w[1118]; Mi{ET1}CG4565[MB02677]
FBti0077590	23824 w[1118]; Mi{ET1}MB02718
FBti0077591	23825 w[1118] Mi{ET1}MB02722
FBti0077592	23826 w[1118]; Mi{ET1}MB02745
FBti0077593	23827 w[1118]; Mi{ET1}pyr[MB02808]/CyO, P{sevRas1.V12}FK1
FBti0077594	23828 w[1118]; Mi{ET1}MB02815
FBti0077595	23829 w[1118]; Mi{ET1}MB02842
FBti0077596	24456 w[1118]; Mi{ET1}MB02863
FBti0077597	23830 w[1118] Mi{ET1}hiw[MB02880] CG5541[MB02880]
FBti0077598	23831 w[1118]; Mi{ET1}MB02887
FBti0077599	23832 w[1118]; Mi{ET1}CG9701[MB02929]
FBti0077600	23833 w[1118]; Mi{ET1}MB02973
FBti0077601	23834 w[1118]; Mi{ET1}gol[MB03006]
FBti0077602	23835 w[1118] Mi{ET1}CG8909[MB03015]
FBti0077603	23836 w[1118]; Mi{ET1}prc[MB03017]/TM6C, Sb[1]
FBti0077604	23837 w[1118]; Mi{ET1}TyrRII[MB03028]
FBti0077606	23839 w[1118]; Mi{ET1}tx[MB03174]
FBti0077607	23840 w[1118] Mi{ET1}CG8028[MB03238]
FBti0077608	23841 w[1118]; Mi{ET1}CG13801[MB03262]

FBti0077609	23842 w[1118] Mi{ET1}Ir10a[MB03273]
FBti0077610	23843 w[1118]; Mi{ET1}CG16838[MB03352]
FBti0077611	23844 w[1118]; Mi{ET1}CG13500[MB03392]
FBti0077612	23845 w[1118]; Mi{ET1}CG7304[MB03467]
FBti0077613	23846 w[1118]; Mi{ET1}Osi4[MB03473]
FBti0077614	23847 w[1118]; Mi{ET1}krimp[MB03478]
FBti0077615	23848 w[1118]; Mi{ET1}MB03485
FBti0077616	23849 w[1118]; Mi{ET1}ACXB[MB03502]
FBti0077617	23850 w[1118] Mi{ET1}CG3632[MB03514] CG9911[MB03514]
FBti0077618	23851 w[1118]; Mi{ET1}CG17189[MB03519]
FBti0077619	23852 w[1118]; Mi{ET1}MB03546
FBti0077620	23853 w[1118] Mi{ET1}CG13014[MB03557]
FBti0077621	23854 w[1118]; Mi{ET1}CG11340[MB03564]
FBti0077623	23855 w[1118] Mi{ET1}MB03596
FBti0077624	24455 w[1118] Mi{ET1}slpr[MB03655]
FBti0077625	23856 w[1118] Mi{ET1}CG12698[MB03675]
FBti0077626	23857 w[1118] Mi{ET1}MB03691
FBti0077627	23858 y[1]; Mi{ET1}sv[MB03697]
FBti0077628	24007 Mi{ET1}MB01990 w[1118]
FBti0077629	24008 w[1118] Mi{ET1}MB02010
FBti0077630	24009 w[1118] Mi{ET1}MB02023
FBti0077631	24010 w[1118]; Mi{ET1}MB02028
FBti0077632	24011 w[1118] Mi{ET1}MB02056
FBti0077633	24012 w[1118]; Mi{ET1}axo[MB02106]
FBti0077634	24013 w[1118]; Mi{ET1}MB02116
FBti0077635	24014 w[1118]; Mi{ET1}CG31221[MB02141]
FBti0077636	24015 w[1118]; Mi{ET1}CG32305[MB02147]
FBti0077637	24016 w[1118]; Mi{ET1}CG31191[MB02232]
FBti0077638	24017 w[1118] Mi{ET1}CG6123[MB02356]
FBti0077639	24440 w[1118]; Mi{ET1}CG3764[MB02387]
FBti0077640	24441 w[1118]; Mi{ET1}cdi[MB02390]
FBti0077641	24018 w[1118]; Mi{ET1}CG9967[MB02469]
FBti0077642	24442 w[1118]; Mi{ET1}fru[MB02472]
FBti0077643	24019 w[1118]; Mi{ET1}CG30116[MB02473]
FBti0077644	24020 w[1118]; Mi{ET1}MB02558
FBti0077645	24021 w[1118]; Mi{ET1}px[MB02577]
FBti0077646	24022 w[1118]; Mi{ET1}Itgβν[MB02607]
FBti0077647	24023 w[1118]; Mi{ET1}M6[MB02608]/TM3, Sb[1] Ser[1]
FBti0077648	24024 w[1118] Mi{ET1}MB02618

FBti0077649	24025 w[1118]; Mi{ET1}NK7.1[MB02679]
FBti0077650	24659 w[1118]; Mi{ET1}CG1273[MB02700]
FBti0077651	24026 w[1118]; Mi{ET1}CG31612[MB02703]
FBti0077652	24027 w[1118]; Mi{ET1}dac[MB02724]
FBti0077653	24028 w[1118]; Mi{ET1}MB02736
FBti0077654	24029 w[1118]; Mi{ET1}MB02740
FBti0077655	24030 w[1118]; Mi{ET1}sif[MB02755]
FBti0077656	24031 w[1118]; Mi{ET1}Pask[MB02780]
FBti0077657	24443 w[1118]; Mi{ET1}Pkc53E[MB02781]
FBti0077658	24032 w[1118]; Mi{ET1}RhoGEF2[MB02788]
FBti0077659	24033 w[1118]; Mi{ET1}Pde1c[MB02790]
FBti0077660	24034 w[1118]; Mi{ET1}MB02806
FBti0077661	24035 w[1118]; Mi{ET1}olf413[MB02820]
FBti0077662	24036 w[1118]; Mi{ET1}CG32982[MB02847]
FBti0077663	24037 w[1118]; Mi{ET1}CG4221[MB02940]
FBti0077664	24038 w[1118]; Mi{ET1}mld[MB02957]
FBti0077665	24039 Mi{ET1}MB02961 w[1118]
FBti0077666	24040 w[1118]; Mi{ET1}CG31174[MB02966]
FBti0077667	24444 Mi{ET1}CG43867[MB02980] w[1118]
FBti0077668	24041 w[1118]; Mi{ET1}CG2010[MB02984]/TM3, Sb[1] Ser[1]
FBti0077669	24042 w[1118]; Mi{ET1}CG31038[MB03005]
FBti0077670	24043 w[1118]; Mi{ET1}MB03027
FBti0077671	24044 w[1118] Mi{ET1}MB03033
FBti0077672	24045 w[1118]; Mi{ET1}grsm[MB03043]
FBti0077673	24046 w[1118]; Mi{ET1}CG33111[MB03048]
FBti0077674	24445 w[1118]; Mi{ET1}sha[MB03098]
FBti0077675	24047 w[1118]; Mi{ET1}CG10960[MB03129]
FBti0077676	24048 w[1118]; Mi{ET1}kuz[MB03175]
FBti0077677	24049 w[1118]; Mi{ET1}Dys[MB03186]
FBti0077678	24050 w[1118]; Mi{ET1}MB03190
FBti0077679	24051 w[1118]; Mi{ET1}CG7737[MB03194]
FBti0077680	24052 Mi{ET1}CG32816[MB03211] w[1118]
FBti0077681	24053 w[1118]; Mi{ET1}MB03216
FBti0077682	24054 w[1118]; Mi{ET1}how[MB03227]
FBti0077683	24055 w[1118]; Mi{ET1}Pvf2[MB03230]
FBti0077684	24446 w[1118]; Mi{ET1}Prosap[MB03234]
FBti0077685	24056 w[1118]; Mi{ET1}timeout[MB03271]
FBti0077686	24057 w[1118]; Mi{ET1}LpR2[MB03322]
FBti0077687	24058 w[1118]; Mi{ET1}Lar[MB03324]

FBti0077688	24059 w[1118]; Mi{ET1}sns[MB03327]
FBti0077689	24060 w[1118]; Mi{ET1}T48[MB03333]
FBti0077690	24061 w[1118]; Mi{ET1}hppy[MB03334]
FBti0077691	24062 w[1118]; Mi{ET1}MB03371
FBti0077692	24063 w[1118]; Mi{ET1}MB03375
FBti0077693	24447 w[1118]; Mi{ET1}CG34347[MB03386]
FBti0077694	24064 w[1118]; Mi{ET1}Pde1c[MB03394]
FBti0077695	24065 w[1118]; Mi{ET1}MB03415
FBti0077696	24066 w[1118]; Mi{ET1}app[MB03425]
FBti0077697	24067 w[1118]; Mi{ET1}tey[MB03446]
FBti0077698	24068 w[1118]; Mi{ET1}Rpb4[MB03453]
FBti0077699	24069 w[1118]; Mi{ET1}MB03462
FBti0077700	24070 w[1118]; Mi{ET1}MB03480
FBti0077701	24071 w[1118]; Mi{ET1}Src64B[MB03494]
FBti0077702	24072 w[1118] Mi{ET1}Evi5[MB03496]
FBti0077703	24073 w[1118]; Mi{ET1}CG31475[MB03509]
FBti0077704	24074 w[1118]; Mi{ET1}MB03530
FBti0077705	24075 w[1118]; Mi{ET1}sano[MB03560]
FBti0077706	24076 w[1118]; Mi{ET1}MB03569
FBti0077707	24077 w[1118]; Mi{ET1}Psa[MB03598] cue[MB03598]
FBti0077708	24078 w[1118]; Mi{ET1}msi[MB03603]
FBti0077709	24079 w[1118]; Mi{ET1}Itp-r83A[MB03611]
FBti0077710	24080 w[1118]; Mi{ET1}CG2022[MB03632]
FBti0077711	24081 w[1118]; Mi{ET1}DLP[MB03646]
FBti0077712	24082 w[1118]; Mi{ET1}CG15630[MB03661]
FBti0077713	24083 w[1118]; Mi{ET1}MB03665
FBti0077714	24448 w[1118]; Mi{ET1}ltd[MB03690]
FBti0077715	24449 w[1118]; Mi{ET1}MB03699
FBti0077716	24084 w[1118]; Mi{ET1}MB03715
FBti0077717	24085 w[1118]; Mi{ET1}cv-c[MB03717]
FBti0077718	24086 w[1118]; Mi{ET1}Fas3[MB03718]
FBti0077719	22662 y[1] w[67c23]; Mi{ET1}Nrx-1[MB00002]
FBti0077720	23946 w[1118] Mi{ET1}CG42339[MB00060]
FBti0077724	23948 y[1] w[67c23]; Mi{ET1}CG14127[MB00306]/TM3, Sb[1] Ser[1]
FBti0077725	23949 w[1118] Mi{ET1}MB00365
FBti0077726	23950 y[1] w[67c23]; Mi{ET1}Ddr[MB00544]
FBti0077727	23951 y[1] w[67c23]; Mi{ET1}CG8745[MB00801]
FBti0077728	23952 w[1118] Mi{ET1}CG12716[MB00898]
FBti0077729	23953 y[1] w[67c23]; Mi{ET1}CG8180[MB01013]

FBti0077730	23954 y[1] w[67c23]; Mi{ET1}CG17097[MB01068]
FBti0077732	23955 y[1] w[67c23]; Mi{ET1}CG12885[MB01208]
FBti0077733	23956 y[1] w[67c23]; Mi{ET1}CG17364[MB01315]
FBti0077734	23957 y[1] w[67c23]; Mi{ET1}MB01602 Mi{ET1}hoe1[MB01602]
FBti0077736	23958 y[1] w[67c23]; Mi{ET1}MB01868
FBti0077737	23959 w[1118]; Mi{ET1}MB01979
FBti0077738	23960 w[1118] Mi{ET1}CG1402[MB01998]
FBti0077739	23961 w[1118]; Mi{ET1}MB02002
FBti0077740	23962 w[1118]; Mi{ET1}MB02038
FBti0077741	23963 w[1118]; Mi{ET1}MB02053
FBti0077742	23964 w[1118]; Mi{ET1}fd68A[MB02091]/TM3, Sb[1] Ser[1]
FBti0077743	23965 w[1118] Mi{ET1}MB02098
FBti0077744	23966 w[1118]; Mi{ET1}MB02169
FBti0077745	23967 w[1118]; Mi{ET1}MB02213
FBti0077746	23968 w[1118]; Mi{ET1}Fili[MB02223]
FBti0077747	23969 w[1118]; Mi{ET1}MB02350
FBti0077748	23970 w[1118]; Mi{ET1}Gγ30A[MB02355]
FBti0077749	23971 y[1] w[67c23]; Mi{ET1}CG18347[MB02413]
FBti0077750	23972 w[1118]; Mi{ET1}Sdc[MB02461]
FBti0077751	23973 w[1118]; Mi{ET1}CG14459[MB02564]
FBti0077752	23974 w[1118]; Mi{ET1}MB02576
FBti0077753	24431 w[1118]; Mi{ET1}MB02610
FBti0077754	23975 w[1118]; Mi{ET1}MB02629
FBti0077755	24432 w[1118]; Mi{ET1}MB02646
FBti0077756	23976 w[1118]; Mi{ET1}MB02653
FBti0077757	23977 w[1118]; Mi{ET1}MB02715
FBti0077758	23978 Mi{ET1}tyn[MB02763] w[1118]
FBti0077759	23979 w[1118]; Mi{ET1}MB02775
FBti0077760	23980 w[1118]; Mi{ET1}MB02821
FBti0077761	23981 w[1118]; Mi{ET1}MB02829
FBti0077762	23982 w[1118]; Mi{ET1}MB02870
FBti0077763	24655 w[1118]; Mi{ET1}MB02901
FBti0077764	23983 w[1118]; Mi{ET1}CG34109[MB02927]
FBti0077765	23984 w[1118]; Mi{ET1}MB02942
FBti0077766	23985 w[1118]; Mi{ET1}MB02947
FBti0077768	23986 y[1] w[67c23]; Mi{ET1}CG5758[MB02967] Mi{ET1}CG31751[MB02967]
FBti0077769	23987 w[1118]; Mi{ET1}MB02969
FBti0077770	23988 w[1118]; Mi{ET1}MB03025
FBti0077771	24433 w[1118]; Mi{ET1}MB03049

FBti0077772	24436 w[1118]; Mi{ET1}pog[MB03078]
FBti0077773	24434 w[1118]; Mi{ET1}MB03085
FBti0077774	24657 w[1118]; Mi{ET1}MB03091
FBti0077775	23989 w[1118]; Mi{ET1}MB03099
FBti0077776	23990 w[1118]; Mi{ET1}MB03113
FBti0077777	24435 w[1118]; Mi{ET1}pHCl[MB03119]/TM6C, cu[1] Sb[1]
FBti0077778	23991 w[1118]; Mi{ET1}MB03142
FBti0077779	23992 w[1118]; Mi{ET1}MB03160
FBti0077780	23993 w[1118]; Mi{ET1}MB03171
FBti0077781	24437 w[1118]; Mi{ET1}MB03181
FBti0077783	23995 w[1118]; Mi{ET1}MB03206
FBti0077784	23996 w[1118]; Mi{ET1}MB03257
FBti0077785	23997 w[1118]; Mi{ET1}MB03344
FBti0077786	23998 w[1118]; Mi{ET1}MB03347
FBti0077787	24438 w[1118]; Mi{ET1}nAcRα-34E[MB03361]
FBti0077788	24439 w[1118]; Mi{ET1}MB03362
FBti0077789	23999 w[1118]; Mi{ET1}MB03387
FBti0077790	24000 w[1118]; Mi{ET1}MB03452
FBti0077791	24001 w[1118]; Mi{ET1}cv-c[MB03489]
FBti0077792	24002 y[1] w[67c23]; Mi{ET1}toe[MB03498]
FBti0077794	24003 w[1118]; Mi{ET1}MB03562
FBti0077795	24004 w[1118]; Mi{ET1}MB03563
FBti0077797	24006 w[1118]; Mi{ET1}CG15256[MB03720]
FBti0078136	24522 w[1118] Mi{ET1}Ptp10D[MB02913]
FBti0078137	24523 w[1118]; Mi{ET1}CG9717[MB03722]
FBti0078138	24524 w[1118] Mi{ET1}shakB[MB03735]
FBti0078139	24525 w[1118]; Mi{ET1}Cip4[MB03744]
FBti0078140	24526 w[1118]; Mi{ET1}CG33993[MB03752]
FBti0078142	24528 w[1118]; Mi{ET1}CG32436[MB03782]
FBti0078143	24529 w[1118] Mi{ET1}rg[MB03792]
FBti0078144	24530 w[1118]; Mi{ET1}CG32432[MB03794]
FBti0078145	24531 Mi{ET1}CG2652[MB03796] w[1118]
FBti0078146	24532 w[1118] Mi{ET1}CG9518[MB03810] Flo-2[MB03810]
FBti0078147	24533 w[1118]; Mi{ET1}GluRIB[MB03843]
FBti0078148	24534 w[1118]; Mi{ET1}sosie[MB03846]
FBti0078149	24535 w[1118]; Mi{ET1}MB03854/SM6a
FBti0078150	24536 w[1118]; Mi{ET1}CG18586[MB03855]
FBti0078151	24537 w[1118]; Mi{ET1}Ets65A[MB03856]
FBti0078152	24538 w[1118]; Mi{ET1}CG14636[MB03866]

FBti0078153	24539 w[1118]; Mi{ET1}MB03870
FBti0078154	24540 w[1118]; Mi{ET1}CG13833[MB03871]
FBti0078155	24541 w[1118]; Mi{ET1}CG33093[MB03875]
FBti0078156	24542 w[1118] Mi{ET1}CG4301[MB03877]
FBti0078157	24543 w[1118]; Mi{ET1}MB03892/TM6C, Sb[1]
FBti0078158	24544 w[1118] Mi{ET1}MB03893
FBti0078159	24545 w[1118]; Mi{ET1}CG34355[MB03916]/TM6C, Sb[1]
FBti0078160	24546 w[1118]; Mi{ET1}MB03934
FBti0078161	24547 w[1118]; Mi{ET1}GluClα[MB03937]
FBti0078162	24548 w[1118]; Mi{ET1}MB03940
FBti0078163	24549 w[1118]; Mi{ET1}TpnC73F[MB03946]/TM6C, Sb[1]
FBti0078164	24550 w[1118]; Mi{ET1}beat-IIa[MB03955]
FBti0078165	24551 w[1118]; Mi{ET1}MB03963/TM6C, Sb[1]
FBti0078166	24789 w[1118]; Mi{ET1}CG33275[MB03965]
FBti0078167	24552 w[1118]; Mi{ET1}MB03974
FBti0078168	24553 w[1118]; Mi{ET1}dpr4[MB03978]
FBti0078169	24554 w[1118]; Mi{ET1}rn[MB03984]
FBti0078170	24555 w[1118]; Mi{ET1}CG6845[MB03988]
FBti0078171	24556 w[1118]; Mi{ET1}MB03990
FBti0078172	24557 w[1118]; Mi{ET1}CG15096[MB03994]
FBti0078173	24558 w[1118]; Mi{ET1}MB04009
FBti0078174	24559 w[1118]; Mi{ET1}MB04014
FBti0078175	24560 w[1118]; Mi{ET1}CG14955[MB04016]
FBti0078176	24561 w[1118]; Mi{ET1}Gnf1[MB04029]
FBti0078177	24562 w[1118]; Mi{ET1}ps[MB04043]/TM6C, Sb[1]
FBti0078178	24563 Mi{ET1}CG32816[MB04052] w[1118]
FBti0078179	24564 w[1118]; Mi{ET1}CG10738[MB04068]/TM6C, Sb[1]
FBti0078180	24565 w[1118]; Mi{ET1}MB04070
FBti0078181	24566 w[1118]; Mi{ET1}MB04079
FBti0078182	24567 w[1118]; Mi{ET1}Mrtf[MB04134]
FBti0078183	24568 w[1118] Mi{ET1}CG15347[MB04140]
FBti0078184	24569 w[1118]; Mi{ET1}Pka-R1[MB04145]
FBti0078185	24570 w[1118]; Mi{ET1}MB04156
FBti0078186	24571 w[1118] Mi{ET1}MB04159
FBti0078187	24572 w[1118] Mi{ET1}stnB[MB04192] stnA[MB04192]
FBti0078188	24573 w[1118] Mi{ET1}CG15043[MB04204]
FBti0078189	24574 w[1118] Mi{ET1}CG15765[MB04213]
FBti0078190	24575 w[1118]; Mi{ET1}sick[MB04228]/SM6a
FBti0078191	24576 w[1118] Mi{ET1}CG32698[MB04308]

FBti0078192	24577 w[1118] Mi{ET1}Ten-a[MB04333]
FBti0078193	24578 w[1118]; Mi{ET1}SCAP[MB04378]/SM6a
FBti0078194	24579 w[1118]; Mi{ET1}MB04385
FBti0078195	24580 w[1118]; Mi{ET1}Ir52c[MB04402]
FBti0078196	24581 w[1118] Mi{ET1}Cap[MB04420]
FBti0078197	24582 w[1118]; Mi{ET1}CG18011[MB04424]/SM6a
FBti0078198	24583 Mi{ET1}MB04449 w[1118]
FBti0078199	24584 w[1118] Mi{ET1}MB04487
FBti0078200	24585 w[1118] Mi{ET1}Fas2[MB04516]
FBti0078201	24586 w[1118]; Mi{ET1}Xpd[MB04535]/SM6a
FBti0078202	24791 w[1118]; Mi{ET1}CG42732[MB04544]
FBti0078203	24587 w[1118]; Mi{ET1}Vmat[MB04557]
FBti0078204	24588 w[1118]; Mi{ET1}CG8216[MB04572]
FBti0078205	24589 Mi{ET1}CG4281[MB04586] w[1118]
FBti0078206	24590 w[1118]; Mi{ET1}Ir85a[MB04613] Pif1A[MB04613]
FBti0078207	24591 w[1118]; Mi{ET1}MB04635
FBti0078208	24592 w[1118]; Mi{ET1}CG6024[MB04686]
FBti0078209	24593 w[1118]; Mi{ET1}Cyp49a1[MB04922] Gαo[MB04922]
FBti0078210	24594 w[1118]; Mi{ET1}CG43333[MB04923]
FBti0078211	24595 w[1118]; Mi{ET1}CG13868[MB04925] CG8920[MB04925]
FBti0078212	24790 w[1118]; Mi{ET1}pgant4[MB04930]/SM6a
FBti0078214	24597 w[1118] Mi{ET1}CG9059[MB04978]
FBti0078215	24598 w[1118]; Mi{ET1}CG8086[MB04989]
FBti0078216	24599 w[1118]; Mi{ET1}CG10600[MB04990]/SM6a
FBti0078217	24600 w[1118]; Mi{ET1}fab1[MB05024] CG33981[MB05024]/SM6a
FBti0078218	24601 w[1118] Mi{ET1}CG32694[MB05037]
FBti0078219	24602 w[1118]; Mi{ET1}hale[MB05052]
FBti0078220	24603 Mi{ET1}sdk[MB05054] w[1118]
FBti0078221	24604 w[1118] Mi{ET1}MB05057
FBti0078222	24605 w[1118] Mi{ET1}Drak[MB05079]
FBti0078223	24606 w[1118]; Mi{ET1}Camta[MB05101]
FBti0078224	24607 w[1118]; Mi{ET1}CG13601[MB05127]
FBti0078225	24608 w[1118]; Mi{ET1}Gr36b[MB05141]
FBti0078226	24609 w[1118]; Mi{ET1}CG4706[MB05145]
FBti0078227	24610 w[1118]; Mi{ET1}Ir64a[MB05283]
FBti0078228	24611 w[1118]; Mi{ET1}Ncc69[MB05292]
FBti0078229	24612 w[1118]; Mi{ET1}Gasp[MB05317]
FBti0078230	24660 w[1118] Mi{ET1}Dok[MB03742]
FBti0078231	24661 w[1118]; Mi{ET1}MB03766

FBti0078232	24662 Mi{ET1}sgg[MB03827] w[1118]
FBti0078233	24663 w[1118] Mi{ET1}MB03890
FBti0078234	24664 w[1118]; Mi{ET1}MB03947
FBti0078235	24665 w[1118]; Mi{ET1}htt[MB03997]
FBti0078236	24666 w[1118]; Mi{ET1}bru-3[MB04010]
FBti0078237	24667 w[1118]; Mi{ET1}CG6966[MB04011]
FBti0078238	24668 w[1118]; Mi{ET1}CG7945[MB04046]
FBti0078239	24669 w[1118]; Mi{ET1}MB04059
FBti0078240	24670 w[1118]; Mi{ET1}Top2[MB04073]
FBti0078241	24671 w[1118]; Mi{ET1}MB04076
FBti0078242	24672 w[1118]; Mi{ET1}CG12207[MB04082]/TM6C, cu[1] Sb[1]
FBti0078243	24673 w[1118]; Mi{ET1}dpr6[MB04096]
FBti0078244	24674 w[1118] Mi{ET1}Flo-2[MB04118]
FBti0078245	24675 w[1118]; Mi{ET1}CG32369[MB04121]
FBti0078246	24676 w[1118]; Mi{ET1}dys[MB04139]
FBti0078247	24677 w[1118]; Mi{ET1}MB04158
FBti0078248	24678 w[1118]; Mi{ET1}CG15864[MB04166]
FBti0078250	24680 w[1118] Mi{ET1}CG33639[MB04209]
FBti0078251	24681 w[1118]; Mi{ET1}CG34113[MB04218]
FBti0078252	24682 w[1118]; Mi{ET1}CG14838[MB04232]/TM6C, cu[1] Sb[1]
FBti0078253	24683 w[1118]; Mi{ET1}Csas[MB04236]/TM6C, cu[1] Sb[1]
FBti0078254	24684 w[1118] Mi{ET1}MB04269
FBti0078255	24685 w[1118]; Mi{ET1}MB04270/TM6C, cu[1] Sb[1]
FBti0078256	24686 w[1118]; Mi{ET1}Hsc70-2[MB04273]
FBti0078257	24687 w[1118]; Mi{ET1}CG5027[MB04280]/TM6C, cu[1] Sb[1]
FBti0078258	24688 w[1118]; Mi{ET1}MB04283
FBti0078259	24689 w[1118]; Mi{ET1}PH4αSG1[MB04290]
FBti0078260	24690 w[1118]; Mi{ET1}CG2187[MB04299]/TM6C, cu[1] Sb[1]
FBti0078261	24691 w[1118]; Mi{ET1}mkg-p[MB04324] CG33057[MB04324]
FBti0078262	24692 w[1118]; Mi{ET1}MB04330
FBti0078263	24693 w[1118]; Mi{ET1}CG14521[MB04344]
FBti0078264	24694 w[1118]; Mi{ET1}Npc2b[MB04347]
FBti0078265	24695 w[1118]; Mi{ET1}CG33124[MB04348]
FBti0078266	24696 w[1118]; Mi{ET1}CG4893[MB04349]
FBti0078267	24697 w[1118]; Mi{ET1}Dhc93AB[MB04366]
FBti0078268	24698 w[1118]; Mi{ET1}CG43367[MB04375]
FBti0078269	24699 w[1118]; Mi{ET1}AdoR[MB04401]
FBti0078270	24700 w[1118]; Mi{ET1}cert[MB04417]
FBti0078271	24701 w[1118]; Mi{ET1}MB04426

FBti0078272	24702 w[1118]; Mi{ET1}CG42668[MB04427]
FBti0078273	24703 w[1118]; Mi{ET1}MB04428
FBti0078274	24704 w[1118]; Mi{ET1}MB04440
FBti0078275	24705 w[1118]; Mi{ET1}5-HT7[MB04445] CG31008[MB04445]
FBti0078276	24706 w[1118]; Mi{ET1}MB04447
FBti0078277	24707 w[1118]; Mi{ET1}beat-VII[MB04459]
FBti0078278	24708 w[1118]; Mi{ET1}tnc[MB04464]
FBti0078279	24709 w[1118]; Mi{ET1}CG42673[MB04477]
FBti0078280	24710 w[1118]; Mi{ET1}beat-IV[MB04480]
FBti0078281	24711 w[1118]; Mi{ET1}p130CAS[MB04490]
FBti0078282	24712 w[1118] Mi{ET1}Cht6[MB04492]
FBti0078283	24713 w[1118]; Mi{ET1}MB04494
FBti0078284	24714 w[1118]; Mi{ET1}MB04496
FBti0078285	24715 w[1118]; Mi{ET1}Ank2[MB04517] CG32373[MB04517]
FBti0078286	24716 w[1118]; Mi{ET1}Eip63F-1[MB04523]
FBti0078287	24717 w[1118]; Mi{ET1}MB04524
FBti0078288	24718 w[1118]; Mi{ET1}Smvt[MB04530]
FBti0078289	24719 w[1118]; Mi{ET1}CG7504[MB04533]
FBti0078290	24720 w[1118]; Mi{ET1}Oatp58Db[MB04546]
FBti0078291	24721 w[1118]; Mi{ET1}exo70[MB04553]/TM6C, cu[1] Sb[1]
FBti0078292	24722 w[1118]; Mi{ET1}CG42575[MB04559]
FBti0078293	24723 w[1118]; Mi{ET1}CG9300[MB04640]
FBti0078294	24724 w[1118] Mi{ET1}CG12535[MB04655]
FBti0078295	24725 Mi{ET1}Pdfr[MB04737] w[1118]
FBti0078296	24726 w[1118]; Mi{ET1}MB04812
FBti0078297	24727 w[1118] Mi{ET1}dnc[MB04869]
FBti0078298	24728 w[1118]; Mi{ET1}Or67b[MB04909] CG8336[MB04909]
FBti0078299	24729 w[1118]; Mi{ET1}CG33090[MB04916]
FBti0078300	24730 w[1118] Mi{ET1}Neto[MB04917]
FBti0078301	24731 w[1118]; Mi{ET1}CG14125[MB04957]
FBti0078302	24732 w[1118]; Mi{ET1}pip[MB04958]
FBti0078303	24733 w[1118]; Mi{ET1}CG14304[MB04967]
FBti0078304	24734 w[1118]; Mi{ET1}CG10654[MB04970]
FBti0078305	24735 w[1118]; Mi{ET1}pHCl[MB04971]
FBti0078306	24736 w[1118]; Mi{ET1}CG9674[MB04992]
FBti0078307	24737 w[1118]; Mi{ET1}chinmo[MB04995]
FBti0078308	24738 w[1118]; Mi{ET1}Cep135[MB04996] CG16959[MB04996]/TM6C, cu[1] Sb[1]
FBti0078309	24739 w[1118]; Mi{ET1}CG14662[MB05012]
FBti0078310	24740 w[1118]; Mi{ET1}MB05032

FBti0078311	24741 w[1118]; Mi{ET1}CG13800[MB05048]
FBti0078312	24742 w[1118]; Mi{ET1}CG3669[MB05085]
FBti0078313	24743 w[1118]; Mi{ET1}Dop1R2[MB05108]
FBti0078314	24744 w[1118]; Mi{ET1}CG31272[MB05128]
FBti0078315	24745 w[1118]; Mi{ET1}CG33158[MB05135]
FBti0078316	24746 w[1118]; Mi{ET1}CG14298[MB05140]
FBti0078317	24747 w[1118]; Mi{ET1}mfr[MB05150]
FBti0078318	24748 w[1118]; Mi{ET1}Lerp[MB05321]
FBti0078319	24179 w[1118] Mi{ET1}MB01991
FBti0078320	24180 w[1118] Mi{ET1}X11Lβ[MB02137]
FBti0078321	24181 w[1118] Mi{ET1}Sh[MB02366]
FBti0078322	24182 w[1118]; Mi{ET1}Dys[MB02524]
FBti0078323	24183 w[1118] Mi{ET1}MB02884
FBti0078324	24184 w[1118] Mi{ET1}eag[MB03032]
FBti0078325	24185 w[1118] Mi{ET1}MB03073
FBti0078327	24186 w[1118] Mi{ET1}CG4991[MB03239]
FBti0078328	24653 w[1118] Mi{ET1}SK[MB03486]
FBti0078329	24187 w[1118] Mi{ET1}DAAM[MB03627]
FBti0078330	24793 w[1118] Mi{ET1}dpr8[MB03631]
FBti0078331	24188 w[1118]; Mi{ET1}Oatp58Dc[MB03731]
FBti0078332	24189 w[1118]; Mi{ET1}CG9850[MB03741]
FBti0078333	24190 w[1118]; Mi{ET1}Gr28b[MB03888]
FBti0078334	24191 w[1118]; Mi{ET1}CG5142[MB03894]
FBti0078335	24192 w[1118]; Mi{ET1}CG13786[MB03902]
FBti0078336	24193 w[1118]; Mi{ET1}CG5756[MB03913]
FBti0078337	24194 w[1118]; Mi{ET1}CG17337[MB03927]
FBti0078338	24195 w[1118]; Mi{ET1}MB03933
FBti0078339	24196 w[1118]; Mi{ET1}MB03966
FBti0078340	24197 w[1118]; Mi{ET1}CLIP-190[MB04222]
FBti0078341	24792 w[1118]; Mi{ET1}MB04266
FBti0078342	24198 w[1118]; Mi{ET1}CG11192[MB04335]
FBti0078343	24199 w[1118]; Mi{ET1}Elk[MB04525]
FBti0078344	24200 w[1118]; Mi{ET1}CG14107[MB04579]
FBti0078345	24201 w[1118]; Mi{ET1}qless[MB04580]
FBti0078346	24202 w[1118]; Mi{ET1}jim[MB04583]
FBti0078347	24203 w[1118]; Mi{ET1}CG31211[MB04599]
FBti0078348	24204 w[1118]; Mi{ET1}CG13793[MB04609]
FBti0078349	24205 w[1118]; Mi{ET1}Ir75d[MB04616]
FBti0078350	24206 w[1118]; Mi{ET1}wkd[MB04624]

FBti0078351	24207 w[1118]; Mi{ET1}CG9451[MB04628]
FBti0078352	24208 w[1118]; Mi{ET1}CG42335[MB04630]
FBti0078353	24209 w[1118]; Mi{ET1}MB04632/CyO
FBti0078355	24211 w[1118]; Mi{ET1}CG34362[MB04653]
FBti0078356	24212 w[1118]; Mi{ET1}FMRFaR[MB04659]
FBti0078357	24656 w[1118]/Dp(1;Y)y[+]; Mi{ET1}MB04662
FBti0078358	24213 w[1118]; Mi{ET1}Tdc1[MB04664] CG9422[MB04664]
FBti0078359	24214 w[1118]; Mi{ET1}CG3290[MB04669]
FBti0078361	24215 w[1118]; Mi{ET1}CG7886[MB04687]
FBti0078362	24216 w[1118]; Mi{ET1}CG1544[MB04693]
FBti0078363	24217 w[1118]; Mi{ET1}CG34380[MB04706]/CyO
FBti0078364	24218 w[1118]; Mi{ET1}amon[MB04710]
FBti0078365	24219 w[1118]; Mi{ET1}ImpL2[MB04721]
FBti0078366	24220 w[1118]; Mi{ET1}RhoGEF64C[MB04730]
FBti0078367	24221 w[1118]; Mi{ET1}MB04740
FBti0078368	24222 w[1118]; Mi{ET1}ft[MB04750]
FBti0078369	24223 w[1118]; Mi{ET1}sr[MB04757]
FBti0078370	24224 w[1118]; Mi{ET1}CG17362[MB04765]
FBti0078371	24225 w[1118]; Mi{ET1}sick[MB04779]
FBti0078372	24226 w[1118]; Mi{ET1}CG5867[MB04780]
FBti0078373	24227 w[1118]; Mi{ET1}CG14502[MB04791]
FBti0078374	24228 w[1118]; Mi{ET1}Pkg21D[MB04805]
FBti0078375	24229 w[1118]; Mi{ET1}ds[MB04809]
FBti0078376	24230 w[1118]; Mi{ET1}MB04815
FBti0078377	24231 w[1118]; Mi{ET1}MB04833
FBti0078378	24232 w[1118]; Mi{ET1}MB04851
FBti0078379	24233 w[1118]; Mi{ET1}MB04856
FBti0078380	24234 w[1118]; Mi{ET1}MB04888
FBti0078381	24235 w[1118]; Mi{ET1}CadN[MB05059]/CyO
FBti0078382	24236 w[1118]; Mi{ET1}kon[MB05095]
FBti0078383	24237 w[1118]; Mi{ET1}spir[MB05102]
FBti0078385	24239 w[1118]; Mi{ET1}CG5073[MB05167]
FBti0078386	24240 w[1118]; Mi{ET1}5-HT1B[MB05181]
FBti0078387	24241 w[1118]; Mi{ET1}CG11842[MB05183]
FBti0078388	24242 w[1118]; Mi{ET1}MB05184
FBti0078389	24243 w[1118]; Mi{ET1}Sema-1a[MB05185]
FBti0078390	24244 w[1118]; Mi{ET1}CG8861[MB05189]
FBti0078391	24245 w[1118]; Mi{ET1}wnd[MB05191]
FBti0078392	24246 w[1118] Mi{ET1}CG34325[MB05200]

FBti0078393	24658 w[1118]; Mi{ET1}zwilch[MB05212]
FBti0078394	24247 w[1118]; Mi{ET1}CG14736[MB05213]
FBti0078395	24248 w[1118]; Mi{ET1}Cpr65Eb[MB05220]
FBti0078396	24249 w[1118]; Mi{ET1}MB05253
FBti0078397	24250 w[1118]; Mi{ET1}CG7213[MB05259]
FBti0078398	24251 w[1118]; Mi{ET1}Cyp6d2[MB05269]
FBti0078399	24252 w[1118]; Mi{ET1}MB05284
FBti0078400	24253 w[1118]; Mi{ET1}MB05289
FBti0078401	24254 w[1118]; Mi{ET1}CG5792[MB05293]
FBti0078402	24255 w[1118]; Mi{ET1}IA-2[MB05302]
FBti0078403	24256 w[1118]; Mi{ET1}MB05319/CyO
FBti0078404	24257 w[1118]; Mi{ET1}mim[MB05320]
FBti0078405	24258 w[1118]; Mi{ET1}MB05322
FBti0078406	24259 w[1118]; Mi{ET1}CG11406[MB05328]
FBti0078407	24260 w[1118]; Mi{ET1}CG30069[MB05330]/CyO
FBti0078408	24261 w[1118]; Mi{ET1}MB02881
FBti0078409	24262 Mi{ET1}Cyp4d14[MB03260] w[1118]
FBti0078410	24263 w[1118]; Mi{ET1}A2bp1[MB03305]
FBti0078411	24264 w[1118]; Mi{ET1}MB03733
FBti0078412	24265 w[1118]; Mi{ET1}CG12484[MB03736]
FBti0078413	24266 w[1118]; Mi{ET1}CG15099[MB03737]/CyO
FBti0078414	24267 w[1118]; Mi{ET1}MB03746
FBti0078415	24268 w[1118]; Mi{ET1}Vha100-5[MB03748]
FBti0078416	24269 w[1118]; Mi{ET1}CG7203[MB03761]
FBti0078417	24270 w[1118]; Mi{ET1}CG18812[MB03763]
FBti0078419	24272 y[1]; Mi{ET1}bt[MB03788]
FBti0078420	24273 w[1118]; Mi{ET1}MB03799/CyO
FBti0078421	24274 w[1118]; Mi{ET1}MB03803
FBti0078422	24275 w[1118]; Mi{ET1}βTub97EF[MB03812]
FBti0078423	24276 w[1118]; Mi{ET1}MB03817
FBti0078424	24277 w[1118]; Mi{ET1}CG43444[MB03826]
FBti0078425	24278 w[1118]; Mi{ET1}Cpr51A[MB03837]
FBti0078426	24279 y[1]; Mi{ET1}Caps[MB03912]
FBti0078427	24280 w[1118]; Mi{ET1}MB03931
FBti0078428	24281 w[1118]; Mi{ET1}MB03982
FBti0078429	24282 w[1118]; Mi{ET1}Glut4EF[MB03996]
FBti0078430	24283 w[1118]; Mi{ET1}Nos[MB04018]
FBti0078431	24284 w[1118]; Mi{ET1}LanB2[MB04039]/TM3, Sb[1] Ser[1]
FBti0078432	24285 w[1118]; Mi{ET1}CG8475[MB04049]

FBti0078433	24286 w[1118]; Mi{ET1}Cct2[MB04086]
FBti0078434	24287 w[1118]; Mi{ET1}CG32111[MB04092]
FBti0078435	24288 w[1118]; Mi{ET1}CG8349[MB04116]
FBti0078436	24289 w[1118]; Mi{ET1}MB04125
FBti0078437	24290 w[1118]; Mi{ET1}Lip4[MB04130]
FBti0078438	24291 w[1118]; Mi{ET1}CG30158[MB04149]
FBti0078439	24292 w[1118]; Mi{ET1}CG13954[MB04151]
FBti0078440	24293 w[1118]; Mi{ET1}CG31974[MB04153]
FBti0078441	24294 w[1118]; Mi{ET1}MB04176
FBti0078442	24295 w[1118]; Mi{ET1}MB04193
FBti0078443	24296 w[1118]; Mi{ET1}CG3409[MB04207]
FBti0078444	24297 w[1118]; Mi{ET1}beat-Ib[MB04216]
FBti0078445	24298 w[1118]; Mi{ET1}CG14669[MB04223]
FBti0078446	24299 w[1118]; Mi{ET1}CG10361[MB04239]
FBti0078447	24300 w[1118]; Mi{ET1}MB04264
FBti0078448	24301 w[1118]; Mi{ET1}MB04380
FBti0078449	24302 w[1118]; Mi{ET1}MB04383
FBti0078450	24303 w[1118]; Mi{ET1}MB04429
FBti0078451	24304 w[1118]; Mi{ET1}Prosβ4R2[MB04520]
FBti0078453	24305 w[1118]; Mi{ET1}MB04542
FBti0078454	24306 w[1118]; Mi{ET1}CG4374[MB04620]
FBti0078455	24307 w[1118] Mi{ET1}Mur11Da[MB04770]
FBti0078456	24308 w[1118]; Mi{ET1}CG5973[MB04855]
FBti0078457	24309 w[1118]; Mi{ET1}CG13196[MB04892] Buffy[MB04892]
FBti0078458	24310 w[1118]; Mi{ET1}CG4382[MB04920]
FBti0078459	24311 w[1118]; Mi{ET1}hbn[MB04955]
FBti0078460	24312 w[1118]; Mi{ET1}Mdr49[MB04959]
FBti0078461	24313 w[1118]; Mi{ET1}jp[MB04981]
FBti0078462	24314 w[1118]; Mi{ET1}Hydr1[MB04988]
FBti0078463	24315 w[1118]; Mi{ET1}Cyp6a16[MB05000]
FBti0078464	24316 w[1118]; Mi{ET1}unc-104[MB05021]
FBti0078465	24317 w[1118]; Mi{ET1}MB05035
FBti0078466	24318 w[1118]; Mi{ET1}Vha68-2[MB05049]
FBti0078467	24319 w[1118]; Mi{ET1}MB05072
FBti0078468	24320 w[1118]; Mi{ET1}knrl[MB05076]
FBti0078469	24321 w[1118]; Mi{ET1}chp[MB05115]
FBti0078470	24322 w[1118]; Mi{ET1}Ack-like[MB05119]
FBti0078471	24323 w[1118]; Mi{ET1}MB05126
FBti0078472	24324 w[1118]; Mi{ET1}garz[MB05159]

FBti0078473	24325 w[1118]; Mi{ET1}CG34383[MB05180]
FBti0078474	24326 w[1118]; Mi{ET1}Shal[MB05249]
FBti0078475	24327 w[1118]; Mi{ET1}MB05266
FBti0078476	24328 w[1118]; Mi{ET1}MB05268
FBti0078477	24329 w[1118]; Mi{ET1}MB05291
FBti0078478	24330 w[1118]; Mi{ET1}Ndae1[MB05294]
FBti0078479	24331 w[1118]; Mi{ET1}rn[MB05313]
FBti0078480	24332 w[1118]; Mi{ET1}CG5621[MB05324]
FBti0078481	24333 w[1118]; Mi{ET1}Oatp74D[MB05332]/TM3, Sb[1] Ser[1]
FBti0100384	23303 y[1] w[67c23]; Mi{ET1}side[MB00161]
FBti0100385	23725 y[1] w[67c23]; Mi{ET1}wge[MB00520]
FBti0100386	23727 y[1]; Mi{ET1}MED26[MB00704]
FBti0100388	23305 y[1] w[67c23]; Mi{ET1}spz6[MB01006]
FBti0100389	23306 y[1]; Mi{ET1}PMCA[MB01085]
FBti0100390	23307 y[1] w[67c23]; Mi{ET1}DOR[MB01323]
FBti0100391	23308 y[1] w[67c23]; Mi{ET1}CG42268[MB01328]
FBti0100392	23309 y[1] w[67c23]; Mi{ET1}CG9850[MB01396]
FBti0100393	23310 y[1] w[67c23]; Mi{ET1}MB01400
FBti0100394	23311 y[1] w[67c23]; Mi{ET1}ftz-f1[MB01411]
FBti0100395	23312 y[1] w[67c23]; Mi{ET1}Meltrin[MB01428]/TM3, Sb[1] Ser[1]
FBti0100396	23313 y[1] w[67c23]; Mi{ET1}fng[MB01443]
FBti0100398	23315 y[1] w[67c23]; Mi{ET1}fz2[MB01455]
FBti0100399	23316 y[1] w[67c23]; Mi{ET1}Lkr[MB01467]
FBti0100400	23317 y[1] w[67c23]; Mi{ET1}MB01469
FBti0100401	23318 y[1] w[67c23]; Mi{ET1}MB01470
FBti0100402	23319 y[1] w[67c23]; Mi{ET1}Nrx-IV[MB01471]
FBti0100403	23320 y[1] w[67c23]; Mi{ET1}MB01472
FBti0100404	23388 y[1] w[67c23]; Mi{ET1}MB01473
FBti0100405	23321 y[1] w[67c23]; Mi{ET1}MB01475
FBti0100406	23322 y[1] w[67c23]; Mi{ET1}mRpL43[MB01480]/CyO
FBti0100407	23323 y[1] w[67c23]; Mi{ET1}CG16908[MB01483]/TM3, Sb[1] Ser[1]
FBti0100408	23324 y[1] w[67c23]; Mi{ET1}sif[MB01486]
FBti0100409	23325 y[1] w[67c23]; Mi{ET1}MB01493
FBti0100410	23326 y[1] w[67c23]; Mi{ET1}Ac76E[MB01510]
FBti0100411	23327 y[1] w[67c23]; Mi{ET1}sty[MB01515]
FBti0100412	23328 y[1] w[67c23]; Mi{ET1}MB01523
FBti0100413	23329 y[1] w[67c23]; Mi{ET1}MB01524
FBti0100414	23330 y[1] w[67c23]; Mi{ET1}CG7987[MB01525]
FBti0100415	23331 y[1] w[67c23]; Mi{ET1}rgn[MB01529]

FBti0100416	23332 y[1] w[67c23]; Mi{ET1}htl[MB01532]
FBti0100417	23333 y[1] w[67c23]; Mi{ET1}MB01535
FBti0100418	23334 y[1] w[67c23]; Mi{ET1}CG42669[MB01547]
FBti0100419	23335 y[1] w[67c23]; Mi{ET1}CG18622[MB01553]
FBti0100420	23336 y[1] w[67c23]; Mi{ET1}Syn1[MB01557]
FBti0100421	23337 y[1] w[67c23]; Mi{ET1}MB01626
FBti0100422	23338 w[1118] Mi{ET1}lz[MB01646]
FBti0100423	23339 w[1118] Mi{ET1}Graf[MB01672]
FBti0100425	23340 y[1] w[67c23]; Mi{ET1}CG14691[MB01673]
FBti0100426	23341 y[1] w[67c23]; Mi{ET1}alph[MB01683]
FBti0100427	23342 y[1] w[67c23]; Mi{ET1}ltl[MB01691]
FBti0100428	23343 y[1] w[67c23]; Mi{ET1}Dys[MB01698]
FBti0100429	23344 w[1118] Mi{ET1}RunxB[MB01709]
FBti0100430	23345 w[1118] Mi{ET1}MB01710
FBti0100431	23346 y[1] w[67c23]; Mi{ET1}MB01718/TM3, Sb[1] Ser[1]
FBti0100432	23347 y[1] w[67c23]; Mi{ET1}CG43143[MB01720]
FBti0100433	23348 w[1118] Mi{ET1}MB01723
FBti0100434	23349 y[1] w[67c23]; Mi{ET1}MB01724
FBti0100435	23350 y[1] w[67c23]; Mi{ET1}MB01729
FBti0100436	23351 y[1] w[67c23]; Mi{ET1}CG17666[MB01732]
FBti0100437	23352 y[1] w[67c23]; Mi{ET1}CG14857[MB01745]
FBti0100438	23353 y[1] w[67c23]; Mi{ET1}GLaz[MB01748]
FBti0100439	23354 y[1] w[67c23]; Mi{ET1}MB01753
FBti0100440	23355 y[1] w[67c23]; Mi{ET1}AlCR2[MB01754]
FBti0100441	23356 y[1] w[67c23]; Mi{ET1}pHCl[MB01759]
FBti0100442	23357 y[1] w[67c23]; Mi{ET1}Glut1[MB01761]
FBti0100443	23358 y[1] w[67c23]; Mi{ET1}timeout[MB01771]
FBti0100444	23359 y[1] w[67c23]; Mi{ET1}Elo68α[MB01772]
FBti0100445	23360 y[1] w[67c23]; Mi{ET1}MB01776
FBti0100446	23361 y[1] w[67c23]; Mi{ET1}MB01784
FBti0100447	23362 y[1] w[67c23]; Mi{ET1}CG17917[MB01790]
FBti0100448	23363 y[1] w[67c23]; Mi{ET1}CG12974[MB01796]
FBti0100449	23364 y[1] w[67c23]; Mi{ET1}CG6332[MB01798]
FBti0100450	23365 w[1118] Mi{ET1}MB01800
FBti0100451	23366 y[1] w[67c23]; Mi{ET1}MB01803
FBti0100452	23367 y[1] w[67c23]; Mi{ET1}CG7896[MB01808]
FBti0100453	23368 y[1] w[67c23]; Mi{ET1}CG42268[MB01816]
FBti0100454	23369 y[1] w[67c23]; Mi{ET1}CG44836[MB01828]
FBti0100455	23370 y[1] w[67c23]; Mi{ET1}MB01835

FBti0100456	23371 w[1118] Mi{ET1}rg[MB01845]
FBti0100457	23372 w[1118] Mi{ET1}CG42258[MB01846]
FBti0100458	23373 y[1] w[67c23]; Mi{ET1}MB01848/TM3, Sb[1] Ser[1]
FBti0100459	23374 y[1] w[67c23]; Mi{ET1}Glut1[MB01876]
FBti0100460	23375 w[1118] Mi{ET1}MB01892
FBti0100461	23376 y[1] w[67c23]; Mi{ET1}CSN7[MB01896]/CyO
FBti0100462	23377 y[1] w[67c23]; Mi{ET1}CG8046[MB01906]
FBti0100463	23378 y[1] w[67c23]; Mi{ET1}fry[MB01915]
FBti0100464	23379 y[1] w[67c23]; Mi{ET1}Osi19[MB01924]
FBti0100465	23380 y[1] w[67c23]; Mi{ET1}Spn[MB01935]
FBti0100466	23381 y[1] w[67c23]; Mi{ET1}Hml[MB01940]
FBti0100467	23382 y[1] w[67c23]; Mi{ET1}LKR[MB01942]
FBti0100469	23384 y[1] w[67c23]; Mi{ET1}CG42708[MB01945]
FBti0100471	23386 y[1] w[67c23]; Mi{ET1}Eip78C[MB01950]
FBti0100472	23387 y[1] w[67c23]; Mi{ET1}Gfrl[MB01951]
FBti0100477	24796 w[1118]; Mi{ET1}MB01149
FBti0100478	24797 w[1118]; Mi{ET1}Elk[MB01176]
FBti0100479	24798 w[1118]; Mi{ET1}MB01794
FBti0100480	24799 w[1118]; Mi{ET1}Pde1c[MB02052] CG31704[MB02052]
FBti0100481	24800 w[1118]; Mi{ET1}CG32017[MB02477]
FBti0100482	24801 w[1118]; Mi{ET1}CG30088[MB03014]
FBti0100484	24803 w[1118]; Mi{ET1}sug[MB03297]/SM6a
FBti0100485	24804 w[1118]; Mi{ET1}tn[MB03490]
FBti0100486	24805 w[1118]; Mi{ET1}MB04184/SM6a
FBti0100487	24806 w[1118]; Mi{ET1}Ets98B[MB04306]
FBti0100488	24807 w[1118]; Mi{ET1}CG14299[MB04322]
FBti0100489	24808 w[1118]; Mi{ET1}ara[MB04323]/TM6C, Sb[1]
FBti0100490	24809 w[1118]; Mi{ET1}Hip1[MB04365]
FBti0100491	24810 w[1118]; Mi{ET1}Ir93a[MB04433]
FBti0100492	24811 w[1118]; Mi{ET1}slo[MB04469]
FBti0100493	24812 w[1118]; Mi{ET1}CG7724[MB04508]/TM6C, Sb[1]
FBti0100495	24813 w[1118]; Mi{ET1}CAP[MB04531] Mi{ET1}Prx2540-2[MB04531]
FBti0100496	24814 w[1118]; Mi{ET1}CG9990[MB04550]/TM6C, Sb[1]
FBti0100497	24815 w[1118]; Mi{ET1}MB04575
FBti0100498	24816 w[1118]; Mi{ET1}MB04699
FBti0100499	24817 w[1118]; Mi{ET1}MB04731
FBti0100500	24818 w[1118]; Mi{ET1}MB04772
FBti0100501	24819 w[1118]; Mi{ET1}Octβ3R[MB04794]
FBti0100502	24820 w[1118]; Mi{ET1}mesh[MB04800]

FBti0100503	24821 w[1118]; Mi{ET1}CG33255[MB04810]
FBti0100504	24822 w[1118]; Mi{ET1}MB04813
FBti0100505	24823 w[1118]; Mi{ET1}CG34113[MB04817]
FBti0100506	24824 w[1118]; Mi{ET1}CG5873[MB04819]/TM6C, Sb[1]
FBti0100507	24825 w[1118]; Mi{ET1}Ilk[MB04821]/TM6C, Sb[1]
FBti0100508	24826 w[1118]; Mi{ET1}CG14380[MB04838]
FBti0100509	24827 w[1118]; Mi{ET1}fau[MB04842]
FBti0100510	24828 w[1118]; Mi{ET1}SKIP[MB04854]
FBti0100511	24829 w[1118]; Mi{ET1}ckd[MB04861]
FBti0100512	24830 w[1118]; Mi{ET1}CG6498[MB04862]
FBti0100513	24831 w[1118]; Mi{ET1}CG6839[MB04865]
FBti0100514	24832 w[1118]; Mi{ET1}cal1[MB04866]/TM6C, Sb[1]
FBti0100515	24833 w[1118]; Mi{ET1}sba[MB04880]
FBti0100516	24834 w[1118]; Mi{ET1}Ugt86Dj[MB04890]
FBti0100517	24835 w[1118]; Mi{ET1}CG31145[MB04900]
FBti0100518	24836 w[1118]; Mi{ET1}MB04908
FBti0100519	24837 w[1118]; Mi{ET1}mth[MB04912] Ptpmeg[MB04912]
FBti0100520	24838 w[1118]; Mi{ET1}MB04914/TM6C, Sb[1]
FBti0100521	24839 w[1118]; Mi{ET1}ara[MB04926]
FBti0100522	24840 w[1118]; Mi{ET1}CG10226[MB04934]
FBti0100523	24841 w[1118]; Mi{ET1}MB04944
FBti0100524	24842 w[1118]; Mi{ET1}CG13060[MB04946]
FBti0100525	24843 w[1118]; Mi{ET1}Ptp99A[MB04947]
FBti0100526	24844 w[1118]; Mi{ET1}CG17150[MB05004]/TM6C, Sb[1]
FBti0100527	24845 w[1118]; Mi{ET1}CG11889[MB05006] CG11891[MB05006]
FBti0100528	24846 w[1118]; Mi{ET1}MB05007
FBti0100529	24847 w[1118]; Mi{ET1}CG7956[MB05039]
FBti0100530	24848 w[1118]; Mi{ET1}CG4613[MB05077]
FBti0100531	24849 w[1118]; Mi{ET1}MB05082
FBti0100532	24850 w[1118]; Mi{ET1}Mal-A1[MB05107]
FBti0100533	24851 w[1118]; Mi{ET1}MB05114
FBti0100534	24852 w[1118]; Mi{ET1}CG12768[MB05134]
FBti0101589	25212 w[1118]; Mi{ET1}Ir52b[MB02231]/SM6a
FBti0101590	25213 y[1]; Mi{ET1}Ank[MB02705]
FBti0101591	25214 w[1118]; Mi{ET1}Clbn[MB03253]/TM6C, Sb[1]
FBti0101592	25215 w[1118]; Mi{ET1}CG4398[MB03695]
FBti0101593	25216 w[1118]; Mi{ET1}MB03842
FBti0101594	25217 w[1118]; Mi{ET1}Slc45-1[MB04353]
FBti0101595	26325 w[*]; Mi{ET1}CG14869[MB04384]/TM6B, Tb[1]

FBti0101596	25218 w[1118]; Mi{ET1}CG17193[MB04985]
FBti0101597	25219 w[1118]; Mi{ET1}CG10096[MB05136] CG10097[MB05136] CG34402[MB05136]
FBti0101598	25220 w[1118] Mi{ET1}su(f)[MB05229] CG17159[MB05229] CG17162[MB05229]
FBti0101599	25221 w[1118]; Mi{ET1}l(2)34Fc[MB05436]
FBti0101600	25222 w[1118]; Mi{ET1}luna[MB05472]
FBti0101601	25223 w[1118]; Mi{ET1}proPO45[MB05593]
FBti0101602	25224 w[1118]; Mi{ET1}Ac78C[MB05598]
FBti0101603	25225 w[1118]; Mi{ET1}MB05603
FBti0101604	25226 w[1118]; Mi{ET1}MB05607
FBti0101605	25227 w[1118]; Mi{ET1}MB05611
FBti0101606	25228 w[1118]; Mi{ET1}MB05626
FBti0101607	25229 w[1118]; Mi{ET1}CG15861[MB05703]
FBti0101608	25230 w[1118]; Mi{ET1}MB05704
FBti0101609	25231 w[1118]; Mi{ET1}cpx[MB05709]
FBti0101610	25232 w[1118]; Mi{ET1}CG6071[MB05713]
FBti0101611	25233 w[1118]; Mi{ET1}Hmx[MB05774]
FBti0101612	25234 w[1118]; Mi{ET1}aret[MB05908]
FBti0101613	25235 w[1118]; Mi{ET1}Hr51[MB05909]
FBti0101614	25236 w[1118]; Mi{ET1}klg[MB05977]
FBti0101615	25237 w[1118]; Mi{ET1}p120ctn[MB06056]
FBti0101616	25238 w[1118]; Mi{ET1}CG15666[MB06062]
FBti0101617	25239 w[1118]; Mi{ET1}CG12428[MB06416]
FBti0101618	25240 w[1118]; Mi{ET1}sick[MB06621]
FBti0101619	25241 w[1118]; Mi{ET1}CG16965[MB06672]
FBti0101620	25242 w[1118]; Mi{ET1}CadN[MB06686]
FBti0101621	25243 w[1118]; Mi{ET1}MB06691
FBti0101622	25244 w[1118]; Mi{ET1}Pkd2[MB06703]
FBti0101623	25245 w[1118]; Mi{ET1}Sgs5[MB06704]
FBti0101624	25246 w[1118]; Mi{ET1}CG34126[MB06705]
FBti0101625	25247 w[1118]; Mi{ET1}CG10211[MB06712]
FBti0101626	25248 w[1118]; Mi{ET1}MB06722/SM6a
FBti0101627	25249 w[1118]; Mi{ET1}fu2[MB06723]
FBti0101628	25250 w[1118]; Mi{ET1}MB06731
FBti0101629	25251 w[1118]; Mi{ET1}Kat60[MB06739]
FBti0101630	25252 w[1118]; Mi{ET1}CG33458[MB06740]
FBti0101631	25253 w[1118]; Mi{ET1}Lgr3[MB06848]
FBti0101632	25254 w[1118]; Mi{ET1}CG5023[MB06849]
FBti0101633	25255 w[1118]; Mi{ET1}MB06861
FBti0101634	25256 w[1118]; Mi{ET1}dpr10[MB07155]

FBti0101635	25257 w[1118]; Mi{ET1}CG31016[MB07157]
FBti0101636	25258 w[1118]; Mi{ET1}Osi18[MB07173]
FBti0101637	25259 w[1118]; Mi{ET1}CG1513[MB07184]
FBti0101638	25260 w[1118]; Mi{ET1}Hdc[MB07212]
FBti0101639	25261 w[1118]; Mi{ET1}MB07220
FBti0101640	25262 w[1118]; Mi{ET1}Mes2[MB07230]
FBti0101641	25263 w[1118]; Mi{ET1}Cpr50Ca[MB07237]
FBti0101642	25264 w[1118]; Mi{ET1}MB07253b Mi{ET1}MB07253a
FBti0101644	25265 w[1118]; Mi{ET1}CG32196[MB07264]
FBti0101645	25266 w[1118]; Mi{ET1}CG14712[MB07271]/TM6C, Sb[1]
FBti0101646	25267 w[1118]; Mi{ET1}CadN[MB07278]
FBti0101647	25268 w[1118]; Mi{ET1}Nckx30C[MB07279]
FBti0101648	25269 w[1118]; Mi{ET1}CG30463[MB07284]
FBti0101649	25270 w[1118]; Mi{ET1}MB07286
FBti0101650	25271 w[1118]; Mi{ET1}Sp212[MB07536]
FBti0101651	25272 w[1118]; Mi{ET1}MB07538
FBti0101652	25273 w[1118]; Mi{ET1}Rim[MB07541]
FBti0101653	25274 w[1118]; Mi{ET1}MB07645
FBti0101654	25275 w[1118]; Mi{ET1}CG7675[MB07646]/TM6C, Sb[1]
FBti0101655	25276 w[1118]; Mi{ET1}MB07665
FBti0101656	25277 w[1118]; Mi{ET1}Ppn[MB07666]
FBti0101657	25278 w[1118]; Mi{ET1}MB07677
FBti0101658	25279 w[1118]; Mi{ET1}CG30049[MB07714]
FBti0101659	25280 w[1118]; Mi{ET1}MB07723
FBti0101660	25281 w[1118]; Mi{ET1}MB07788
FBti0101661	25282 w[1118]; Mi{ET1}MB07878
FBti0101662	25283 w[1118]; Mi{ET1}CG15534[MB07929]
FBti0101663	25284 w[1118]; Mi{ET1}MB07957
FBti0101664	25285 w[1118]; Mi{ET1}dpr15[MB07991]
FBti0101665	25286 w[1118]; Mi{ET1}CG32121[MB08045]
FBti0101666	25287 w[1118]; Mi{ET1}MB08195
FBti0101667	25288 w[1118]; Mi{ET1}MB08198
FBti0101668	25289 w[1118]; Mi{ET1}mp[MB08228]
FBti0101669	25290 w[1118]; Mi{ET1}MB08269
FBti0101670	25291 y[1] w[67c23]; Mi{ET1}mspo[MB00082]
FBti0101671	25292 w[1118]; Mi{ET1}CG30048[MB05216]
FBti0101672	25293 w[1118]; Mi{ET1}ppk19[MB05382]
FBti0101673	25294 w[1118]; Mi{ET1}CG4751[MB05404]
FBti0101674	25295 w[1118]; Mi{ET1}Dscam4[MB05408]

FBti0101675	25296 w[1118]; Mi{ET1}MB05423
FBti0101676	25297 w[1118]; Mi{ET1}MB05443
FBti0101677	25298 w[1118]; Mi{ET1}Dhc93AB[MB05444]
FBti0101679	25300 w[1118] Mi{ET1}Neto[MB05569]
FBti0101680	25301 w[1118]; Mi{ET1}CG34245[MB05581]
FBti0101681	25302 w[1118]; Mi{ET1}vimar[MB05601]
FBti0101682	25303 w[1118]; Mi{ET1}Rab9[MB05612]
FBti0101683	25304 w[1118]; Mi{ET1}CG30054[MB05666]
FBti0101684	25305 w[1118]; Mi{ET1}MB05672
FBti0101685	25306 w[1118]; Mi{ET1}CG11876[MB05732]
FBti0101686	25307 w[1118]; Mi{ET1}CG42389[MB05860]
FBti0101687	25308 w[1118]; Mi{ET1}Rme-8[MB05876]/SM6a
FBti0101688	25309 w[1118]; Mi{ET1}CG42672[MB05883]
FBti0101689	25310 w[1118]; Mi{ET1}CG8665[MB05897]
FBti0101690	25311 w[1118]; Mi{ET1}140up[MB05907]/TM6C, Sb[1]
FBti0101691	25312 w[1118] Mi{ET1}MB05922
FBti0101692	25313 w[1118]; Mi{ET1}MB05926
FBti0101693	25314 w[1118]; Mi{ET1}MB05927
FBti0101694	25315 w[1118]; Mi{ET1}dpr3[MB06000]
FBti0101696	25317 w[1118]; Mi{ET1}mbl[MB06084]
FBti0101697	25318 w[1118]; Mi{ET1}Nckx30C[MB06102]
FBti0101698	25319 w[1118]; Mi{ET1}CG42235[MB06284]
FBti0101699	25320 w[1118]; Mi{ET1}CG4577[MB06386]
FBti0101700	25321 w[1118]; Mi{ET1}CG8419[MB06410]
FBti0101701	25322 w[1118]; Mi{ET1}CG30268[MB06424]
FBti0101702	25323 w[1118]; Mi{ET1}wb[MB06467]
FBti0101703	25324 w[1118]; Mi{ET1}MB06490
FBti0101704	25325 w[1118]; Mi{ET1}CG7742[MB06587]
FBti0101705	25326 w[1118]; Mi{ET1}beat-Ic[MB06591]
FBti0101706	25327 w[1118]; Mi{ET1}CG12038[MB06619]
FBti0101707	25328 w[1118]; Mi{ET1}Myo95E[MB06620]
FBti0101708	25329 w[1118]; Mi{ET1}MB06625
FBti0101709	25330 w[1118]; Mi{ET1}Rbp6[MB06777]
FBti0101710	25331 w[1118]; Mi{ET1}CG31145[MB06827]
FBti0101711	25332 w[1118]; Mi{ET1}CG8768[MB06845] nemy[MB06845]
FBti0101712	25333 w[1118]; Mi{ET1}MB06847
FBti0101713	25334 w[1118]; Mi{ET1}otk[MB06876]
FBti0101714	25335 w[1118]; Mi{ET1}MB06884
FBti0101715	25336 w[1118]; Mi{ET1}MB06890

FBti0101716	25337 w[1118]; Mi{ET1}eIF3-S9[MB06914]
FBti0101717	25338 w[1118]; Mi{ET1}gry[MB06920]/TM6C, Sb[1]
FBti0101718	25339 w[1118]; Mi{ET1}pHCl[MB06931]/TM6C, Sb[1]
FBti0101719	25340 w[1118]; Mi{ET1}CG42324[MB06933]
FBti0101720	25341 w[1118]; Mi{ET1}Oli[MB06951]
FBti0101721	25342 w[1118]; Mi{ET1}CG32204[MB06966]
FBti0101722	25343 w[1118]; Mi{ET1}TotA[MB06977]
FBti0101723	25344 w[1118]; Mi{ET1}a[MB06991]
FBti0101724	25345 w[1118]; Mi{ET1}Dh44[MB07006]
FBti0101725	25346 w[1118]; Mi{ET1}CG7971[MB07314]
FBti0101726	25347 w[1118]; Mi{ET1}CG30463[MB07317]
FBti0101727	25348 w[1118]; Mi{ET1}dsx-c73A[MB07320] CG33158[MB07320]
FBti0101728	25349 w[1118]; Mi{ET1}MB07391
FBti0101729	25350 w[1118]; Mi{ET1}CG13380[MB07480] CG18233[MB07480] CG4174[MB07480]
FBti0101730	25351 w[1118]; Mi{ET1}zfh1[MB07519]
FBti0101731	25352 w[1118]; Mi{ET1}MB07523
FBti0101732	25353 w[1118]; Mi{ET1}MB07525
FBti0101733	25354 w[1118]; Mi{ET1}CG8907[MB07528]
FBti0101735	25356 w[1118]; Mi{ET1}MB07826
FBti0101736	25357 w[1118]; Mi{ET1}MB07844
FBti0101737	25358 w[1118]; Mi{ET1}MB07882
FBti0101738	25359 w[1118]; Mi{ET1}MB07895
FBti0101739	25360 w[1118] Mi{ET1}MB07928
FBti0101740	25361 w[1118]; Mi{ET1}Sec61α[MB07934]
FBti0101741	25362 w[1118]; Mi{ET1}MB07955
FBti0101742	25363 w[1118]; Mi{ET1}Tor[MB07988]
FBti0101743	25364 w[1118]; Mi{ET1}CG31668[MB08037]
FBti0101744	25365 w[1118]; Mi{ET1}Afti[MB08087]/TM6C, Sb[1]
FBti0101745	25366 w[1118]; Mi{ET1}timeout[MB08132]
FBti0101746	25367 w[1118]; Mi{ET1}MB08135
FBti0101747	25368 w[1118]; Mi{ET1}CG13800[MB08137]
FBti0101748	25369 w[1118]; Mi{ET1}TotZ[MB08193]
FBti0101749	25370 w[1118]; Mi{ET1}CG16786[MB08255]
FBti0101884	25446 w[1118]; Mi{ET1}MB05340
FBti0101885	25447 w[1118]; Mi{ET1}MB05342/SM6a
FBti0101886	25448 w[1118]; Mi{ET1}MB05376
FBti0101887	25449 w[1118]; Mi{ET1}MB05406
FBti0101888	25450 w[1118]; Mi{ET1}CG18519[MB05465]
FBti0101889	25451 w[1118]; Mi{ET1}CG13654[MB05466]

FBti0101890	25452 w[1118]; Mi{ET1}MB05476
FBti0101891	25453 w[1118]; Mi{ET1}Nup37[MB05519]
FBti0101892	25454 w[1118]; Mi{ET1}MB05551
FBti0101894	25456 w[1118] Mi{ET1}CG34120[MB05594]
FBti0101895	25457 w[1118]; Mi{ET1}MB05655
FBti0101896	25458 w[1118]; Mi{ET1}MB05702
FBti0101897	25459 w[1118] Mi{ET1}rg[MB05724]
FBti0101898	25460 w[1118]; Mi{ET1}Snoo[MB05731]
FBti0101899	25461 w[1118]; Mi{ET1}CG6232[MB05750]
FBti0101900	25462 w[1118]; Mi{ET1}dpr3[MB05777]
FBti0101901	25463 w[1118]; Mi{ET1}CG42321[MB05800]
FBti0101902	25464 w[1118] Mi{ET1}CG14435[MB05816]
FBti0101903	25465 w[1118] Mi{ET1}CG12119[MB05824]
FBti0101904	25466 w[1118]; Mi{ET1}CG44153[MB05845]
FBti0101905	25467 w[1118] Mi{ET1}CG15452[MB05881]
FBti0101906	25468 w[1118]; Mi{ET1}tinc[MB05904]
FBti0101907	25469 w[1118]; Mi{ET1}CG16732[MB05928]
FBti0101908	25470 w[1118]; Mi{ET1}CG33156[MB05931]
FBti0101909	25471 w[1118]; Mi{ET1}MB05946
FBti0101910	25472 w[1118]; Mi{ET1}CG34386[MB05982]
FBti0101911	25473 w[1118]; Mi{ET1}MB05991
FBti0101912	25474 w[1118]; Mi{ET1}CG13028[MB05996]
FBti0101913	25475 w[1118]; Mi{ET1}CG34030[MB06014]
FBti0101914	25476 w[1118] Mi{ET1}Peritrophin-A[MB06101]
FBti0101915	25477 w[1118]; Mi{ET1}MB06113
FBti0101916	25478 w[1118]; Mi{ET1}MB06117
FBti0101917	25479 w[1118]; Mi{ET1}CG5398[MB06136]
FBti0101918	25480 w[1118]; Mi{ET1}pum[MB06187]
FBti0101919	25481 w[1118]; Mi{ET1}CG10168[MB06190]
FBti0101920	25482 w[1118]; Mi{ET1}Nha2[MB06200] Or94b[MB06200]
FBti0101921	25483 w[1118]; Mi{ET1}Pi3K92E[MB06212]/TM6C, Sb[1]
FBti0101922	25484 w[1118]; Mi{ET1}MESR3[MB06218]
FBti0101923	25485 w[1118]; Mi{ET1}Pld[MB06221]
FBti0101924	25486 w[1118]; Mi{ET1}CG8519[MB06237]
FBti0101925	25487 w[1118]; Mi{ET1}MB06239
FBti0101926	25488 w[1118]; Mi{ET1}CG8008[MB06245]
FBti0101927	25489 w[1118]; Mi{ET1}mRpS23[MB06248]/SM6a
FBti0101928	25490 w[1118] Mi{ET1}CG15721[MB06259]
FBti0101929	25491 w[1118]; Mi{ET1}CG6053[MB06262]

FBti0101930	25492 w[1118]; Mi{ET1}CG5565[MB06279]
FBti0101931	25493 w[1118]; Mi{ET1}MB06289
FBti0101932	25494 w[1118]; Mi{ET1}Snoo[MB06294]
FBti0101933	25495 w[1118]; Mi{ET1}CG31445[MB06310]
FBti0101934	25496 w[1118]; Mi{ET1}CG44250[MB06320]
FBti0101935	25497 w[1118]; Mi{ET1}CG17780[MB06329] CG17781[MB06329]/TM6C, Sb[1]
FBti0101936	25498 w[1118]; Mi{ET1}MB06343
FBti0101937	25499 w[1118]; Mi{ET1}MB06365
FBti0101938	25500 w[1118]; Mi{ET1}Obp69a[MB06377] CG32104[MB06377]
FBti0101939	25501 w[1118]; Mi{ET1}Ugt36Bb[MB06388]
FBti0101940	25502 w[1118]; Mi{ET1}mRpL23[MB06390]/TM6C, Sb[1]
FBti0101941	25503 w[1118]; Mi{ET1}Lar[MB06400]
FBti0101942	25504 w[1118]; Mi{ET1}CG5849[MB06403]/TM6C, Sb[1]
FBti0101943	25505 w[1118]; Mi{ET1}MB06407
FBti0101944	25506 w[1118]; Mi{ET1}MB06409
FBti0101945	25507 w[1118]; Mi{ET1}CG34394[MB06414]
FBti0101946	25508 w[1118] Mi{ET1}CG43658[MB06429]
FBti0101947	25509 w[1118]; Mi{ET1}Alk[MB06458]
FBti0101948	25510 w[1118]; Mi{ET1}MB06501
FBti0101949	25511 w[1118]; Mi{ET1}CG10638[MB06512]
FBti0101950	25512 w[1118]; Mi{ET1}Cyp6d4[MB06513]
FBti0101951	25513 w[1118]; Mi{ET1}Hs6st[MB06545] CG31213[MB06545]
FBti0101952	25514 w[1118]; Mi{ET1}CG7910[MB06548]
FBti0101953	25515 w[1118] Mi{ET1}eIF4E-7[MB06554]a Mi{ET1}eIF4E-7[MB06554]b eIF4E-7[MB06554]
FBti0101955	25516 w[1118] Mi{ET1}MB06556
FBti0101956	25517 w[1118]; Mi{ET1}sff[MB06603]
FBti0101957	25518 w[1118]; Mi{ET1}Fur1[MB06661]
FBti0101958	25519 w[1118]; Mi{ET1}nAcRα-30D[MB06675]
FBti0101959	25520 Mi{ET1}CG32816[MB06700] w[1118]
FBti0101960	25521 w[1118]; Mi{ET1}MB06743
FBti0101961	25522 w[1118]; Mi{ET1}CG17082[MB06749]
FBti0101962	25523 w[1118]; Mi{ET1}MB06765
FBti0101963	25524 w[1118]; Mi{ET1}CG13476[MB06767]
FBti0101964	25525 w[1118]; Mi{ET1}CG33704[MB06800]
FBti0101965	25526 w[1118]; Mi{ET1}egr[MB06803]
FBti0101966	25527 w[1118]; Mi{ET1}Ca-α1D[MB06807]/SM6a
FBti0101967	25528 w[1118]; Mi{ET1}MB06831
FBti0101968	25529 w[1118]; Mi{ET1}CG31637[MB06841]
FBti0101969	25530 w[1118]; Mi{ET1}Cyp9c1[MB06857] CG13594[MB06857]

FBti0101970	25531 w[1118]; Mi{ET1}drpr[MB06916]
FBti0101971	25532 w[1118]; Mi{ET1}app[MB06918]/TM6C, Sb[1]
FBti0101972	25533 w[1118]; Mi{ET1}slam[MB06922]/SM6a
FBti0101973	25534 Mi{ET1}CG13377[MB06932] w[1118]
FBti0101974	25535 w[1118]; Mi{ET1}CG17224[MB06944]
FBti0101975	25536 w[1118]; Mi{ET1}Gfrl[MB06972]
FBti0101976	25537 w[1118]; Mi{ET1}MB06988
FBti0101977	25538 w[1118]; Mi{ET1}MB06995
FBti0101978	25539 w[1118]; Mi{ET1}dpr9[MB07019]
FBti0101979	25540 w[1118]; Mi{ET1}MB07020
FBti0101980	25541 w[1118]; Mi{ET1}CG18539[MB07054]
FBti0101981	25542 w[1118]; Mi{ET1}MB07093
FBti0101982	25543 w[1118]; Mi{ET1}CG10513[MB07190]
FBti0101983	25544 w[1118]; Mi{ET1}CG13841[MB07231] Usp12-46[MB07231] CG7029[MB07231]
FBti0101985	25546 w[1118]; Mi{ET1}MB07305
FBti0101986	25547 w[1118]; Mi{ET1}DAT[MB07315]
FBti0101987	25548 w[1118]; Mi{ET1}CG11030[MB07325] CG11147[MB07325]
FBti0101988	25549 w[1118]; Mi{ET1}CG34356[MB07390]
FBti0101989	25550 w[1118]; Mi{ET1}CG2955[MB07433]
FBti0101990	25551 w[1118]; Mi{ET1}Ir94g[MB07445]
FBti0101991	25552 w[1118]; Mi{ET1}Glut1[MB07446]
FBti0101992	25553 w[1118]; Mi{ET1}CG3502[MB07452]
FBti0101993	25554 w[1118]; Mi{ET1}fz[MB07478]
FBti0101994	25555 w[1118]; Mi{ET1}Sap-r[MB07508] CG15547[MB07508]
FBti0101995	25556 w[1118]; Mi{ET1}CG15236[MB07522] CG34215[MB07522]
FBti0101996	25557 w[1118]; Mi{ET1}MB07576
FBti0101997	25558 w[1118]; Mi{ET1}CG31924[MB07582] CG5561[MB07582]
FBti0101998	25559 w[1118]; Mi{ET1}TfIIA-L[MB07587]
FBti0101999	25560 w[1118]; Mi{ET1}atl[MB07599]/TM6C, Sb[1]
FBti0102000	25561 w[1118]; Mi{ET1}CG5910[MB07603]
FBti0102001	25562 w[1118]; Mi{ET1}CAH2[MB07616]
FBti0102002	25563 w[1118]; Mi{ET1}CG11073[MB07687]
FBti0102003	25564 w[1118]; Mi{ET1}CG13284[MB07694]
FBti0102004	25565 w[1118] Mi{ET1}gce[MB07696]
FBti0102005	25566 w[1118]; Mi{ET1}Ser[MB07731]
FBti0102006	25567 w[1118]; Mi{ET1}CG12910[MB07736]
FBti0102007	25568 w[1118]; Mi{ET1}CG15630[MB07747]
FBti0102008	25569 w[1118]; Mi{ET1}MB07791
FBti0102009	25570 w[1118]; Mi{ET1}MB07815

FBti0102010	25571 w[1118]; Mi{ET1}CG44152[MB07822]
FBti0102011	25572 w[1118]; Mi{ET1}CG7290[MB07832]
FBti0102012	25573 w[1118]; Mi{ET1}MB07854
FBti0102013	25574 w[1118]; Mi{ET1}eyg[MB07856]
FBti0102014	25575 w[1118]; Mi{ET1}CG4623[MB07860]
FBti0102015	25576 w[1118]; Mi{ET1}Pph13[MB07864]
FBti0102016	25577 w[1118] Mi{ET1}AlstR[MB07922]
FBti0102017	25578 w[1118]; Mi{ET1}CG16791[MB07933]/TM6C, Sb[1]
FBti0102018	25579 w[1118]; Mi{ET1}Sema-1a[MB07938]
FBti0102020	25581 w[1118]; Mi{ET1}CG13188[MB08023]
FBti0102021	25582 w[1118]; Mi{ET1}MB08040
FBti0102022	25583 w[1118]; Mi{ET1}CG15385[MB08071]
FBti0102023	25584 w[1118]; Mi{ET1}CG16710[MB08106]
FBti0102024	25585 w[1118] Mi{ET1}para[MB08120]
FBti0102025	25586 w[1118]; Mi{ET1}MB08131
FBti0102026	25587 w[1118]; Mi{ET1}CG42368[MB08158]
FBti0102027	25588 w[1118]; Mi{ET1}CG32278[MB08160] CG32281[MB08160]/TM6C, Sb[1]
FBti0102028	25589 w[1118]; Mi{ET1}CG9270[MB08161]
FBti0102029	25590 w[1118]; Mi{ET1}CG32082[MB08199]
FBti0102030	25591 w[1118]; Mi{ET1}ort[MB08221]
FBti0102031	25592 w[1118]; Mi{ET1}CG34375[MB08223]
FBti0102032	25593 w[1118] Mi{ET1}MB08238
FBti0102033	25594 w[1118]; Mi{ET1}crb[MB08251]
FBti0102034	25595 w[1118]; Mi{ET1}Nrx-IV[MB08273] eap[MB08273]/TM6C, Sb[1]
FBti0102035	25596 w[1118]; Mi{ET1}MB08607
FBti0113528	25597 w[1118]; Mi{ET1}MB05415
FBti0113529	25598 w[1118]; Mi{ET1}AttC[MB05438]
FBti0113530	25599 w[1118]; Mi{ET1}Cad96Ca[MB05499]
FBti0113531	25600 w[1118]; Mi{ET1}CG13579[MB05513]
FBti0113532	25601 w[1118]; Mi{ET1}CG31559[MB05527]
FBti0113533	25602 w[1118]; Mi{ET1}CG31139[MB05545]
FBti0113534	25603 w[1118]; Mi{ET1}MB05546
FBti0113535	25604 w[1118]; Mi{ET1}MB05560
FBti0113536	25605 w[1118]; Mi{ET1}CG12947[MB05606]
FBti0113537	25606 w[1118]; Mi{ET1}CG13506[MB05615]
FBti0113539	25608 w[1118]; Mi{ET1}CG3502[MB05778] CG9863[MB05778]
FBti0113540	25609 w[1118]; Mi{ET1}CG43078[MB05780]
FBti0113541	25610 w[1118]; Mi{ET1}CG13700[MB05785]
FBti0113542	25611 w[1118]; Mi{ET1}olf413[MB05868]

FBti0113543	25612 w[1118]; Mi{ET1}Invadolysin[MB05882]
FBti0113544	25613 w[1118]; Mi{ET1}Naam[MB05940]
FBti0113545	25614 w[1118]; Mi{ET1}MsR2[MB05984]
FBti0113546	25615 w[1118]; Mi{ET1}CG6983[MB06049]
FBti0113547	25616 w[1118] Mi{ET1}AlstR[MB06064]
FBti0113548	25617 w[1118]; Mi{ET1}Dscam2[MB06131]
FBti0113549	25618 w[1118]; Mi{ET1}obst-B[MB06214]
FBti0113550	25619 w[1118]; Mi{ET1}MB06303
FBti0113551	25620 w[1118]; Mi{ET1}Tsp42Ec[MB06423]
FBti0113552	25621 w[1118]; Mi{ET1}CG10062[MB06502]
FBti0113553	25622 w[1118]; Mi{ET1}CG43795[MB06507]
FBti0113554	25623 w[1118]; Mi{ET1}MB06523
FBti0113555	25624 w[1118]; Mi{ET1}CG43729[MB06537]
FBti0113556	25625 w[1118]; Mi{ET1}CG33303[MB06540]
FBti0113557	25626 w[1118]; Mi{ET1}H2.0[MB06569]
FBti0113558	25627 w[1118]; Mi{ET1}Drl-2[MB06584]
FBti0113559	25628 w[1118]; Mi{ET1}CG13538[MB06608]
FBti0113560	25629 w[1118]; Mi{ET1}trpγ[MB06664]
FBti0113561	25630 w[1118]; Mi{ET1}CG10631[MB06810]
FBti0113562	25631 w[1118]; Mi{ET1}CG9313[MB06913]
FBti0113563	25632 w[1118]; Mi{ET1}Or30a[MB06924]
FBti0113564	25633 w[1118]; Mi{ET1}CG33465[MB06930]
FBti0113565	25634 Mi{ET1}MB07008 w[1118]
FBti0113566	25635 w[1118]; Mi{ET1}Ance-4[MB07027]
FBti0113567	25636 w[1118]; Mi{ET1}E23[MB07029]
FBti0113568	25637 w[1118]; Mi{ET1}CG9304[MB07102]
FBti0113569	25638 w[1118]; Mi{ET1}Sur[MB07175]
FBti0113570	25639 w[1118]; Mi{ET1}Bili[MB07242]
FBti0113571	25640 w[1118]; Mi{ET1}MB07293
FBti0113572	25641 w[1118]; Mi{ET1}FBX011[MB07449]
FBti0113573	25642 w[1118]; Mi{ET1}Snoo[MB07471]
FBti0113574	25643 w[1118]; Mi{ET1}CG12858[MB07515]
FBti0113575	25644 w[1118]; Mi{ET1}CG34354[MB07567]
FBti0113576	25645 w[1118]; Mi{ET1}CG33120[MB07596]
FBti0113577	25646 w[1118]; Mi{ET1}MB07614
FBti0113578	25647 w[1118]; Mi{ET1}CG43729[MB07623]
FBti0113579	25648 w[1118]; Mi{ET1}MB07628
FBti0113580	25649 w[1118]; Mi{ET1}side[MB07679]
FBti0113581	25650 w[1118]; Mi{ET1}CG3119[MB07697]

FBti0113582	25651 w[1118]; Mi{ET1}CG8001[MB07772]
FBti0113583	25652 w[1118]; Mi{ET1}Tsp42Ei[MB07877]
FBti0113584	25653 w[1118]; Mi{ET1}CG14837[MB07912]
FBti0113585	25654 w[1118]; Mi{ET1}MB08007
FBti0113586	25655 w[1118]; Mi{ET1}Or56a[MB08013]
FBti0113587	25656 w[1118]; Mi{ET1}CG3831[MB08020]
FBti0113588	25657 w[1118]; Mi{ET1}MB08024
FBti0113589	25658 w[1118]; Mi{ET1}Tsp42Eg[MB08050]
FBti0113590	25659 w[1118]; Mi{ET1}MB08067
FBti0113591	25660 w[1118]; Mi{ET1}MB08143
FBti0113592	25661 w[1118]; Mi{ET1}CG9119[MB08166]
FBti0113593	25662 w[1118]; Mi{ET1}CG43373[MB08203]
FBti0113594	25663 w[1118]; Mi{ET1}MB08226
FBti0113595	25664 w[1118]; Mi{ET1}CG17839[MB08271]
FBti0113596	25665 w[1118]; Mi{ET1}CG34353[MB08498]
FBti0113597	25666 w[1118]; Mi{ET1}MB08549
FBti0113934	26026 w[1118]; Mi{ET1}MB05417
FBti0113935	26027 w[1118]; Mi{ET1}MB05515
FBti0113936	26028 w[1118]; Mi{ET1}MB05518
FBti0113937	26029 w[1118]; Mi{ET1}CG14891[MB05531]
FBti0113938	26030 w[1118]; Mi{ET1}GV1[MB05550]
FBti0113939	26031 w[1118]; Mi{ET1}Ir68a[MB05565]
FBti0113940	26032 w[1118]; Mi{ET1}CG4480[MB05664]
FBti0113941	26033 w[1118]; Mi{ET1}Cbl[MB05683]/TM6C, Sb[1]
FBti0113942	26034 w[1118]; Mi{ET1}MB05693
FBti0113943	26035 w[1118]; Mi{ET1}Gfrl[MB05727]
FBti0113944	26036 w[1118]; Mi{ET1}CG15533[MB05761]
FBti0113945	26037 Mi{ET1}MB05779 w[1118]
FBti0113946	26038 w[1118]; Mi{ET1}CG9130[MB05793] CG9133[MB05793]
FBti0113947	26039 w[1118]; Mi{ET1}Mco1[MB05950]
FBti0113948	26040 w[1118]; Mi{ET1}Lim3[MB05992]
FBti0113949	26041 w[1118]; Mi{ET1}Pde6[MB06146]
FBti0113950	26042 w[1118]; Mi{ET1}MB06152
FBti0113951	26043 w[1118]; Mi{ET1}MB06183
FBti0113952	26044 w[1118]; Mi{ET1}CG11889[MB06268] CG11891[MB06268]
FBti0113953	26045 w[1118]; Mi{ET1}Cha[MB06338] VAChT[MB06338]
FBti0113954	26046 w[1118] Mi{ET1}CG2750[MB06379]
FBti0113955	26047 w[1118] Mi{ET1}acj6[MB06436]
FBti0113956	26048 w[1118]; Mi{ET1}Pk92B[MB06487]

FBti0113957	26049 w[1118]; Mi{ET1}sm[MB06488]
FBti0113958	26050 w[1118]; Mi{ET1}Wnk[MB06499]/TM6C, Sb[1]
FBti0113959	26051 w[1118]; Mi{ET1}CG14856[MB06509]
FBti0113960	26052 w[1118] Mi{ET1}MB06515
FBti0113961	26053 w[1118]; Mi{ET1}CG32407[MB06522]
FBti0113962	26054 w[1118]; Mi{ET1}RhoGEF64C[MB06529]
FBti0113963	26055 w[1118]; Mi{ET1}Spec2[MB06551]/TM6C, Sb[1]
FBti0113964	26056 w[1118]; Mi{ET1}CG13830[MB06609]
FBti0113965	26057 w[1118]; Mi{ET1}Indy-2[MB06615] CG17193[MB06615] CG33934[MB06615]
FBti0113966	26058 w[1118]; Mi{ET1}Cpr49Ad[MB06646]
FBti0113967	26059 w[1118] Mi{ET1}CG3626[MB06711]
FBti0113968	26060 w[1118]; Mi{ET1}Cyp316a1[MB06750]
FBti0113969	26061 w[1118] Mi{ET1}rdgA[MB06886] CG10962[MB06886]
FBti0113970	26062 y[1]; Mi{ET1}dpr7[MB06954]
FBti0113971	26063 w[1118]; Mi{ET1}CASK[MB06983]/TM6C, Sb[1]
FBti0113972	26064 w[1118] Mi{ET1}CG14196[MB07005]
FBti0113973	26065 w[1118]; Mi{ET1}RhoBTB[MB07044]
FBti0113974	26066 w[1118]; Mi{ET1}CG14516[MB07062]
FBti0113975	26067 w[1118] Mi{ET1}CG1835[MB07074]
FBti0113976	26068 w[1118] Mi{ET1}CG5877[MB07078]
FBti0113977	26069 w[1118]; Mi{ET1}CG4723[MB07117] Usp12-46[MB07117] CG7029[MB07117]
FBti0113978	26070 Mi{ET1}vnd[MB07124] w[1118]
FBti0113979	26071 w[1118]; Mi{ET1}Pli[MB07138]
FBti0113980	26072 w[1118]; Mi{ET1}EndoG[MB07150]
FBti0113981	26073 w[1118] Mi{ET1}MB07163
FBti0113982	26074 w[1118]; Mi{ET1}Muc26B[MB07167]
FBti0113983	26075 w[1118] Mi{ET1}MB07189
FBti0113984	26076 Mi{ET1}Cyp4d1[MB07195] w[1118]
FBti0113985	26077 w[1118]; Mi{ET1}MB07299
FBti0113986	26078 w[1118]; Mi{ET1}CG10116[MB07306]
FBti0113987	26079 w[1118]; Mi{ET1}CG13671[MB07358]
FBti0113988	26080 w[1118]; Mi{ET1}CG3216[MB07455]
FBti0113989	26503 w[1118]; Mi{ET1}wa-cup[MB07475]
FBti0113990	26081 w[1118]; Mi{ET1}CG8483[MB07625]
FBti0113991	26082 w[1118] Mi{ET1}CG15252[MB07671]
FBti0113992	26083 w[1118]; Mi{ET1}RhoGEF3[MB07682]
FBti0113993	26084 w[1118]; Mi{ET1}mspo[MB07748]
FBti0113994	26085 w[1118]; Mi{ET1}α-Est7[MB07755]
FBti0113995	26086 w[1118]; Mi{ET1}CG2321[MB07796]

FBti0113996	26087 w[1118] Mi{ET1}MB07827
FBti0113997	26088 y[1]; Mi{ET1}toy[MB07851]
FBti0113998	26089 y[1]; Mi{ET1}CG31997[MB07898]
FBti0114000	26090 Mi{ET1}Smr[MB07914]a Mi{ET1}Smr[MB07914]b Smr[MB07914] CG15725[MB07914]
FBti0114001	26091 w[1118]; Mi{ET1}CG13978[MB07972]
FBti0114002	26092 w[1118]; Mi{ET1}soti[MB07998]/TM6C, Sb[1]
FBti0114003	26093 w[1118]; Mi{ET1}CG31198[MB08005]
FBti0114004	26094 w[1118]; Mi{ET1}jvl[MB08021]
FBti0114005	26095 w[1118]; Mi{ET1}PH4αEFB[MB08046]
FBti0114006	26096 w[1118] Mi{ET1}mus101[MB08064]
FBti0114007	26097 w[1118]; Mi{ET1}Gprk2[MB08070]
FBti0114008	26098 w[1118]; Mi{ET1}CG14743[MB08074]
FBti0114009	26099 w[1118]; Mi{ET1}CG18437[MB08151]
FBti0114010	26100 Mi{ET1}Mur2B[MB08153] w[1118]
FBti0114011	26101 w[1118]; Mi{ET1}Vrp1[MB08225]
FBti0114013	26102 w[1118]; Mi{ET1}SKIP[MB08250]a Mi{ET1}SKIP[MB08250]b
FBti0114014	26103 w[1118]; Mi{ET1}CadN2[MB08260]
FBti0114015	26104 w[1118]; Mi{ET1}MB08301
FBti0114016	26105 w[1118]; Mi{ET1}Sesn[MB08308] CG18128[MB08308]
FBti0114017	26106 w[1118]; Mi{ET1}Cry[MB08319]
FBti0114018	26107 w[1118]; Mi{ET1}grp[MB08323]
FBti0114019	26108 w[1118]; Mi{ET1}CG5002[MB08327]
FBti0114020	26109 w[1118]; Mi{ET1}kon[MB08344]
FBti0114021	26110 w[1118]; Mi{ET1}MB08378
FBti0114022	26111 w[1118]; Mi{ET1}CG42820[MB08402]
FBti0114023	26112 w[1118]; Mi{ET1}CG3124[MB08409]
FBti0114024	26113 w[1118]; Mi{ET1}CG3257[MB08418] CG3257[MB08418]
FBti0114025	26114 w[1118]; Mi{ET1}CG10527[MB08435]
FBti0114026	26115 w[1118]; Mi{ET1}MB08451
FBti0114027	26116 w[1118]; Mi{ET1}MB08455
FBti0114028	26117 w[1118]; Mi{ET1}CG12209[MB08460]
FBti0114029	26118 w[1118]; Mi{ET1}MB08492
FBti0114030	26119 w[1118]; Mi{ET1}MB08527
FBti0114031	26120 w[1118]; Mi{ET1}MB08535
FBti0114032	26121 w[1118]; Mi{ET1}CG12866[MB08536]
FBti0114033	26122 w[1118]; Mi{ET1}MB08568
FBti0114034	26123 w[1118]; Mi{ET1}CG12913[MB08570]
FBti0114035	26124 w[1118]; Mi{ET1}Calx[MB08599]
FBti0114036	26125 w[1118]; Mi{ET1}MB08628

FBti0114037	26126 w[1118]; Mi{ET1}MB08639
FBti0114038	26127 w[1118]; Mi{ET1}MB08673
FBti0114039	26128 w[1118]; Mi{ET1}MB08723
FBti0114040	26129 w[1118]; Mi{ET1}Cas[MB08748] mdy[MB08748]/SM6a
FBti0114041	26130 w[1118]; Mi{ET1}MB08752
FBti0114042	26131 w[1118]; Mi{ET1}CG5872[MB08774]
FBti0114043	26132 w[1118]; Mi{ET1}MB08815
FBti0114044	26133 w[1118]; Mi{ET1}Ptp61F[MB08823] CG32320[MB08823]
FBti0114045	26134 w[1118]; Mi{ET1}Spn42Dc[MB08825]
FBti0114046	26135 w[1118]; Mi{ET1}ptip[MB08895]
FBti0114047	26136 w[1118]; Mi{ET1}MB08925
FBti0114048	26137 w[1118]; Mi{ET1}MB08926
FBti0114049	26138 w[1118]; Mi{ET1}ZnT35C[MB08945]
FBti0114050	26139 w[1118]; Mi{ET1}upSET[MB08950]
FBti0114051	26140 w[1118]; Mi{ET1}MB09035
FBti0114052	26141 w[1118]; Mi{ET1}MB09062
FBti0114053	26142 w[1118]; Mi{ET1}yki[MB09079]/SM6a
FBti0114054	26143 w[1118]; Mi{ET1}sut4[MB09128]
FBti0114055	26144 w[1118]; Mi{ET1}Cdc16[MB09129]/TM6C, Sb[1]
FBti0114056	26145 w[1118]; Mi{ET1}CG6758[MB09275]
FBti0114057	26146 w[1118]; Mi{ET1}CG5888[MB09289]
FBti0114058	26147 w[1118]; Mi{ET1}CG9525[MB09338]
FBti0114059	26148 w[1118]; Mi{ET1}MB09528
FBti0114060	26149 w[1118]; Mi{ET1}MB09539
FBti0114061	26150 w[1118]; Mi{ET1}CG17840[MB09541]
FBti0114062	26151 w[1118]; Mi{ET1}CG12011[MB09542]
FBti0114063	26152 w[1118]; Mi{ET1}MB09548
FBti0114064	26153 w[1118]; Mi{ET1}B4[MB09610]
FBti0114065	26154 w[1118]; Mi{ET1}CG8654[MB09631]
FBti0114066	26155 w[1118]; Mi{ET1}CG10089[MB09633]
FBti0114067	26156 w[1118]; Mi{ET1}MB09697/SM6a
FBti0114145	26326 w[1118]; Mi{ET1}Cad89D[MB04472]
FBti0114146	26327 w[1118]; Mi{ET1}MB05517
FBti0114147	26328 w[1118] Mi{ET1}CG5172[MB05660]
FBti0114148	26329 w[1118]; Mi{ET1}MB05905
FBti0114149	26330 w[1118] Mi{ET1}MB06099
FBti0114150	26331 w[1118]; Mi{ET1}CG5177[MB06186]
FBti0114151	26332 w[1118] Mi{ET1}CG17167[MB06236]
FBti0114152	26333 w[1118]; Mi{ET1}Tpl94D[MB06354]

FBti0114153	26334 w[1118]; Mi{ET1}W[MB06404]
FBti0114154	26335 w[1118] Mi{ET1}Neto[MB06658]
FBti0114155	26336 w[1118]; Mi{ET1}CG7220[MB06775]
FBti0114156	26337 w[1118]; Mi{ET1}MB06879
FBti0114157	26338 w[1118] Mi{ET1}Bx[MB06953]
FBti0114158	26339 w[1118]; Mi{ET1}CG13428[MB06969]
FBti0114159	26340 w[1118]; Mi{ET1}CG7695[MB06982]
FBti0114160	26341 w[1118] Mi{ET1}Hers[MB07095]
FBti0114161	26342 w[1118] Mi{ET1}Neto[MB07125]
FBti0114162	26343 w[1118] Mi{ET1}MB07154
FBti0114163	26344 w[1118]; Mi{ET1}CG33322[MB07355]
FBti0114164	26345 w[1118]; Mi{ET1}BG642167[MB07363]
FBti0114165	26346 w[1118]; Mi{ET1}CG32105[MB07364]
FBti0114166	26347 w[1118]; Mi{ET1}Uch-L5[MB07373]
FBti0114167	26348 w[1118]; Mi{ET1}capa[MB07374]/TM6C, Sb[1]
FBti0114168	26349 w[1118]; Mi{ET1}CG7716[MB07394]
FBti0114169	26350 w[1118]; Mi{ET1}CG34426[MB07397]
FBti0114170	26351 w[1118]; Mi{ET1}kug[MB07400]/TM6C, Sb[1]
FBti0114171	26352 w[1118]; Mi{ET1}MB07424
FBti0114172	26353 w[1118]; Mi{ET1}CG8562[MB07675]
FBti0114173	26354 y[1]; Mi{ET1}bt[MB07727]
FBti0114174	26355 w[1118]; Mi{ET1}CG9850[MB07952]
FBti0114175	26356 w[1118]; Mi{ET1}dpr10[MB07963]
FBti0114176	26357 w[1118]; Mi{ET1}MB07992
FBti0114177	26358 w[1118]; Mi{ET1}MB08017
FBti0114178	26359 w[1118]; Mi{ET1}Ate1[MB08111]
FBti0114179	26360 w[1118]; Mi{ET1}MB08152
FBti0114180	26361 w[1118] Mi{ET1}oc[MB08272]
FBti0114181	26362 w[1118]; Mi{ET1}Mhcl[MB08285] CG32855[MB08285]
FBti0114182	26363 w[1118]; Mi{ET1}Pi3K68D[MB08286] CG14131[MB08286]
FBti0114183	26364 w[1118] Mi{ET1}MB08288
FBti0114184	26365 w[1118]; Mi{ET1}MB08339
FBti0114185	26366 w[1118]; Mi{ET1}MB08350
FBti0114186	26367 w[1118] Mi{ET1}MB08361
FBti0114187	26368 w[1118]; Mi{ET1}MB08365
FBti0114188	26369 w[1118]; Mi{ET1}MB08387
FBti0114189	26370 w[1118]; Mi{ET1}CG31427[MB08405]
FBti0114190	26371 w[1118]; Mi{ET1}MB08415
FBti0114191	26372 w[1118]; Mi{ET1}Irk1[MB08423]

FBti0114192	26373 w[1118]; Mi{ET1}beat-Ib[MB08429]
FBti0114193	26374 w[1118]; Mi{ET1}Gr77a[MB08432] CG13252[MB08432]
FBti0114194	26375 w[1118]; Mi{ET1}MB08436
FBti0114195	26376 w[1118]; Mi{ET1}CG6954[MB08442]
FBti0114196	26377 w[1118]; Mi{ET1}ABCB7[MB08445]/TM6C, Sb[1]
FBti0114197	26378 w[1118]; Mi{ET1}MB08452
FBti0114198	26379 w[1118]; Mi{ET1}Tsp26A[MB08474]
FBti0114199	26380 w[1118]; Mi{ET1}cry[MB08493]
FBti0114200	26381 w[1118]; Mi{ET1}mld[MB08505]
FBti0114201	26382 w[1118]; Mi{ET1}MB08510
FBti0114202	26383 w[1118]; Mi{ET1}MB08550
FBti0114203	26384 w[1118] Mi{ET1}Cyp6t1[MB08552]
FBti0114204	26385 w[1118]; Mi{ET1}MB08554
FBti0114206	26387 w[1118]; Mi{ET1}CG31918[MB08562]
FBti0114207	26388 w[1118]; Mi{ET1}MB08564
FBti0114208	26389 w[1118]; Mi{ET1}CG42313[MB08581]
FBti0114209	26390 w[1118]; Mi{ET1}CG16734[MB08597]/TM6C, Sb[1]
FBti0114210	26391 w[1118]; Mi{ET1}MB08630
FBti0114211	26392 w[1118]; Mi{ET1}prominin-like[MB08636]
FBti0114212	26393 w[1118]; Mi{ET1}MB08643
FBti0114214	26395 w[1118]; Mi{ET1}α-Est1[MB08655]
FBti0114215	26396 w[1118]; Mi{ET1}MB08656
FBti0114216	26397 w[1118]; Mi{ET1}Alh[MB08677] Mlp84B[MB08677]
FBti0114217	26398 w[1118]; Mi{ET1}CycG[MB08684] Med[MB08684]/TM6C, Sb[1]
FBti0114218	26399 w[1118]; Mi{ET1}CG14605[MB08700]
FBti0114219	26400 w[1118]; Mi{ET1}Apc[MB08754]
FBti0114220	26401 w[1118]; Mi{ET1}dpr13[MB08759]
FBti0114221	26402 w[1118]; Mi{ET1}CG42784[MB08770]
FBti0114222	26403 w[1118]; Mi{ET1}CG33966[MB08778]
FBti0114223	26404 w[1118]; Mi{ET1}Rdl[MB08800]
FBti0114224	26405 w[1118]; Mi{ET1}CG10688[MB08803]/TM6C, Sb[1]
FBti0114225	26406 w[1118]; Mi{ET1}MB08804
FBti0114226	26407 w[1118] Mi{ET1}mgl[MB08807]
FBti0114227	26408 w[1118]; Mi{ET1}MB08841
FBti0114228	26409 w[1118]; Mi{ET1}CG7461[MB08844]
FBti0114229	26410 w[1118]; Mi{ET1}CG6280[MB08847]
FBti0114230	26411 w[1118]; Mi{ET1}MB08868
FBti0114231	26412 w[1118]; Mi{ET1}MB08881
FBti0114232	26413 w[1118]; Mi{ET1}Cad99C[MB08891]

FBti0114233	26414 w[1118]; Mi{ET1}dsx[MB08902]
FBti0114234	26415 w[1118]; Mi{ET1}CG13830[MB08905] CG17109[MB08905]
FBti0114235	26416 w[1118]; Mi{ET1}CG13085[MB08906]
FBti0114236	26417 w[1118]; Mi{ET1}MB08939
FBti0114237	26418 w[1118]; Mi{ET1}MB08951
FBti0114238	26419 w[1118]; Mi{ET1}MB08955
FBti0114239	26420 w[1118]; Mi{ET1}timeout[MB08962]
FBti0114240	26421 w[1118]; Mi{ET1}Cp19[MB08964]
FBti0114241	26422 w[1118]; Mi{ET1}MB08976
FBti0114242	26423 w[1118] Mi{ET1}Flo-2[MB08977] CG9522[MB08977]
FBti0114243	26424 w[1118]; Mi{ET1}prominin-like[MB08988]
FBti0114244	26425 w[1118]; Mi{ET1}CG31760[MB08990] CG31860[MB08990]
FBti0114245	26426 w[1118]; Mi{ET1}MB08999
FBti0114246	26427 w[1118]; Mi{ET1}Cng[MB09000]
FBti0114247	26428 w[1118]; Mi{ET1}CG15824[MB09005]
FBti0114248	26429 w[1118]; Mi{ET1}CG31229[MB09021]
FBti0114249	26430 w[1118]; Mi{ET1}E(spl)m6-BFM[MB09027]
FBti0114250	26431 w[1118]; Mi{ET1}CG33288[MB09039] barc[MB09039]
FBti0114251	26432 w[1118]; Mi{ET1}MB09050
FBti0114252	26433 w[1118] Mi{ET1}MB09052
FBti0114253	26434 w[1118]; Mi{ET1}CG8475[MB09060]
FBti0114254	26435 w[1118]; Mi{ET1}MB09093
FBti0114255	26436 w[1118]; Mi{ET1}Doc2[MB09116]
FBti0114256	26437 w[1118]; Mi{ET1}Idgf3[MB09118]
FBti0114258	26439 w[1118]; Mi{ET1}MB09123a Mi{ET1}MB09123b
FBti0114259	26439 w[1118]; Mi{ET1}MB09123a Mi{ET1}MB09123b
FBti0114260	26440 w[1118]; Mi{ET1}MB09124
FBti0114261	26441 w[1118]; Mi{ET1}CG6901[MB09130]
FBti0114262	26442 w[1118]; Mi{ET1}CG10625[MB09131]
FBti0114263	26443 w[1118]; Mi{ET1}CG43897[MB09138]
FBti0114264	26444 w[1118] Mi{ET1}MB09143
FBti0114265	26445 w[1118]; Mi{ET1}Acon[MB09176]/SM6a
FBti0114266	26446 w[1118]; Mi{ET1}MB09179
FBti0114267	26447 w[1118]; Mi{ET1}MB09183
FBti0114268	26448 w[1118]; Mi{ET1}CG10713[MB09193]
FBti0114269	26449 w[1118]; Mi{ET1}MB09198
FBti0114270	26450 w[1118]; Mi{ET1}Dgp-1[MB09208]
FBti0114271	26451 w[1118]; Mi{ET1}Amy-d[MB09212]
FBti0114272	26452 w[1118]; Mi{ET1}MB09214

FBti0114273	26453 w[1118]; Mi{ET1}Ir48a[MB09217]
FBti0114274	26454 w[1118]; Mi{ET1}wb[MB09221]
FBti0114275	26455 w[1118]; Mi{ET1}CG3222[MB09257]
FBti0114276	26456 w[1118]; Mi{ET1}MB09259
FBti0114277	26457 w[1118]; Mi{ET1}CG30288[MB09262]
FBti0114278	26458 w[1118]; Mi{ET1}lbl[MB09264]
FBti0114279	26459 w[1118]; Mi{ET1}MB09280
FBti0114280	26460 w[1118]; Mi{ET1}MB09281
FBti0114281	26461 w[1118]; Mi{ET1}Eig71Ec[MB09286]
FBti0114282	26462 w[1118]; Mi{ET1}CG33203[MB09292]
FBti0114283	26463 w[1118]; Mi{ET1}lap[MB09294]
FBti0114284	26464 w[1118]; Mi{ET1}MB09295
FBti0114285	26465 w[1118]; Mi{ET1}Rgk2[MB09302]
FBti0114286	26466 w[1118]; Mi{ET1}MB09310
FBti0114288	26468 w[1118]; Mi{ET1}MB09334
FBti0114289	26469 w[1118]; Mi{ET1}Osi9[MB09340]
FBti0114290	26470 w[1118]; Mi{ET1}MB09351
FBti0114291	26471 w[1118]; Mi{ET1}Takr99D[MB09356]
FBti0114292	26472 w[1118]; Mi{ET1}mwh[MB09358]
FBti0114293	26473 w[1118]; Mi{ET1}CG14545[MB09376]
FBti0114294	26474 w[1118]; Mi{ET1}MB09379
FBti0114295	26475 w[1118]; Mi{ET1}Cyp6d5[MB09381] rdx[MB09381]
FBti0114296	26476 w[1118]; Mi{ET1}cwo[MB09389]
FBti0114297	26477 w[1118]; Mi{ET1}MB09392
FBti0114298	26478 w[1118]; Mi{ET1}CG31100[MB09407]/TM6C, Sb[1]
FBti0114299	26479 w[1118]; Mi{ET1}Ino80[MB09416]
FBti0114300	26480 w[1118]; Mi{ET1}MB09423
FBti0114301	26481 w[1118]; Mi{ET1}MB09434
FBti0114302	26482 w[1118]; Mi{ET1}CG31524[MB09447]
FBti0114303	26483 w[1118]; Mi{ET1}Fer2[MB09480]/TM6C, Sb[1]
FBti0114304	26484 w[1118]; Mi{ET1}Rpn6[MB09493]/SM6a
FBti0114305	26485 w[1118]; Mi{ET1}fus[MB09495]/SM6a
FBti0114306	26486 w[1118] Mi{ET1}MB09508
FBti0114307	26487 w[1118]; Mi{ET1}Tcp-1η[MB09573] CG9839[MB09573]/TM6C, Sb[1]
FBti0114308	26488 w[1118]; Mi{ET1}CG33477[MB09585]
FBti0114309	26489 w[1118]; Mi{ET1}net[MB09620]
FBti0114310	26490 w[1118]; Mi{ET1}Cyp6a8[MB09626]
FBti0114311	26491 w[1118]; Mi{ET1}MB09636
FBti0114312	26492 w[1118]; Mi{ET1}MB09652

FBti0114313	26493 w[1118]; Mi{ET1}MB09670
FBti0114314	26494 w[1118]; Mi{ET1}Calx[MB09693] CG10827[MB09693]
FBti0114315	26495 w[1118]; Mi{ET1}CG7778[MB09695]
FBti0114316	26496 w[1118]; Mi{ET1}Gr36c[MB09703]
FBti0115822	27740 w[1118]; Mi{ET1}CG43896[MB05829]/TM6C, Sb[1]
FBti0115823	27741 w[1118]; Mi{ET1}cln3[MB06009]
FBti0115824	27742 w[1118] Mi{ET1}cac[MB06176]
FBti0115825	27744 w[1118] Mi{ET1}HDAC6[MB06564]
FBti0115826	27745 w[1118]; Mi{ET1}MB06744
FBti0115827	27746 w[1118] Mi{ET1}rdgA[MB07086] CG10962[MB07086]
FBti0115828	27748 w[1118]; Mi{ET1}igl[MB07097]
FBti0115829	27749 w[1118]; Mi{ET1}Rfabg[MB07263]
FBti0115830	27750 w[1118] Mi{ET1}MB07408
FBti0115831	27751 w[1118]; Mi{ET1}MB07415
FBti0115832	27752 w[1118] Mi{ET1}MB07442
FBti0115833	27753 w[1118]; Mi{ET1}aux[MB07514] CG31516[MB07514]
FBti0115834	27754 w[1118]; Mi{ET1}Gαi[MB07632] CR32385[MB07632]
FBti0115835	27755 w[1118]; Mi{ET1}CG13049[MB07761]
FBti0115837	27757 w[1118]; Mi{ET1}CG43980[MB08381]
FBti0115838	27758 w[1118]; Mi{ET1}CG31204[MB08385]
FBti0115839	27759 w[1118]; Mi{ET1}Dys[MB08490]
FBti0115840	27760 w[1118]; Mi{ET1}CG4467[MB08573]
FBti0115841	27761 w[1118]; Mi{ET1}CG31036[MB08574]
FBti0115842	27762 w[1118]; Mi{ET1}Gld[MB08587]
FBti0115843	27763 w[1118]; Mi{ET1}Piezo[MB08675] Piezo[MB08675]
FBti0115844	27764 w[1118] Mi{ET1}Ac13E[MB08864]
FBti0115845	27765 w[1118]; Mi{ET1}MB08874
FBti0115846	27766 w[1118]; Mi{ET1}MB08880
FBti0115847	27767 w[1118]; Mi{ET1}Npc2f[MB08896]
FBti0115848	27768 w[1118]; Mi{ET1}CG33700[MB08920]
FBti0115849	27769 w[1118]; Mi{ET1}MB09112
FBti0115850	27770 w[1118]; Mi{ET1}CG4269[MB09167]
FBti0115851	27771 w[1118]; Mi{ET1}CG13315[MB09204]
FBti0115852	27772 w[1118]; Mi{ET1}CG12814[MB09236]
FBti0115853	27773 w[1118]; Mi{ET1}CG31533[MB09238]
FBti0115854	27774 w[1118]; Mi{ET1}CG18675[MB09311]
FBti0115855	27775 w[1118]; Mi{ET1}MB09324
FBti0115856	27776 w[1118]; Mi{ET1}MB09325
FBti0115857	27777 w[1118] Mi{ET1}SPR[MB09357]

FBti0115858	27778 w[1118]; Mi{ET1}MB09365
FBti0115859	27779 w[1118]; Mi{ET1}Sox100B[MB09390]
FBti0115860	27780 w[1118]; Mi{ET1}cmpy[MB09409]
FBti0115861	27781 w[1118] Mi{ET1}MB09435
FBti0115862	29884 Mi{ET1}Nmdar2[MB09441] w[1118]
FBti0115863	27782 w[1118] Mi{ET1}MB09451
FBti0115864	27783 Mi{ET1}futsch[MB09452] w[1118]
FBti0115865	27784 Mi{ET1}CG3655[MB09468] w[1118]
FBti0115866	27785 w[1118]; Mi{ET1}MB09494
FBti0115867	27786 w[1118]; Mi{ET1}brp[MB09517]
FBti0115868	27787 w[1118]; Mi{ET1}MB09520
FBti0115869	27788 w[1118]; Mi{ET1}MB09526
FBti0115870	27789 w[1118]; Mi{ET1}Ir25a[MB09568]
FBti0115871	27790 w[1118]; Mi{ET1}CG31646[MB09592]
FBti0115872	27791 w[1118]; Mi{ET1}MB09596
FBti0115873	27792 w[1118]; Mi{ET1}MB09608
FBti0115874	27793 w[1118]; Mi{ET1}MB09637
FBti0115875	27794 w[1118] Mi{ET1}MB09639
FBti0115876	27795 w[1118]; Mi{ET1}Lmpt[MB09664]
FBti0115877	27796 w[1118]; Mi{ET1}MB09682
FBti0115878	27797 w[1118]; Mi{ET1}TyrR[MB09692]
FBti0115879	27798 w[1118]; Mi{ET1}CG7337[MB09715]
FBti0115880	27799 w[1118]; Mi{ET1}Muc26B[MB09721]
FBti0115881	27800 w[1118]; Mi{ET1}Gr93d[MB09723]
FBti0115882	27801 w[1118]; Mi{ET1}scro[MB09728]
FBti0115883	27802 w[1118]; Mi{ET1}CG42534[MB09771]
FBti0115884	27803 w[1118]; Mi{ET1}nuf[MB09772]
FBti0115885	27804 w[1118]; Mi{ET1}CG31099[MB09774]
FBti0115886	27805 w[1118]; Mi{ET1}MB09786
FBti0115887	27806 w[1118]; Mi{ET1}CG15256[MB09797]
FBti0115888	27807 w[1118]; Mi{ET1}5-HT1A[MB09812]
FBti0115889	27808 w[1118]; Mi{ET1}T3dh[MB09825]
FBti0115890	27809 w[1118]; Mi{ET1}Ance[MB09828]
FBti0115891	27810 w[1118]; Mi{ET1}nerfin-1[MB09839]
FBti0115892	27811 w[1118]; Mi{ET1}CG13983[MB09845]
FBti0115893	27812 w[1118]; Mi{ET1}MB09859
FBti0115894	27813 w[1118]; Mi{ET1}Ccn[MB09887] CG6034[MB09887]
FBti0115895	27814 w[1118]; Mi{ET1}MB09898
FBti0115896	27815 w[1118]; Mi{ET1}MB09910

FBti0115897	27816 w[1118]; Mi{ET1}CG10051[MB09923]
FBti0115898	27817 w[1118]; Mi{ET1}CG13243[MB09929]
FBti0115899	27818 w[1118]; Mi{ET1}Ir56b[MB09950]
FBti0115900	27819 w[1118]; Mi{ET1}ACXC[MB09973]
FBti0115901	27820 w[1118]; Mi{ET1}5-HT1A[MB09978]
FBti0115902	27821 w[1118]; Mi{ET1}Mal-A7[MB09988]
FBti0115903	27822 w[1118]; Mi{ET1}CG14960[MB09999]
FBti0115904	27823 w[1118]; Mi{ET1}MB10011
FBti0115905	27824 w[1118]; Mi{ET1}CG12069[MB10013]
FBti0115906	27825 w[1118]; Mi{ET1}Galk[MB10014]/SM6a
FBti0115907	27826 w[1118]; Mi{ET1}uif[MB10021]
FBti0115908	27827 w[1118]; Mi{ET1}Trissin[MB10031]
FBti0115909	27828 w[1118]; Mi{ET1}Lsd-1[MB10034]
FBti0115910	27829 w[1118]; Mi{ET1}CG33225[MB10041]
FBti0115911	27830 w[1118]; Mi{ET1}CG12502[MB10071]
FBti0115912	27831 w[1118]; Mi{ET1}MB10075
FBti0115913	27832 w[1118]; Mi{ET1}Ero1L[MB10117]
FBti0115914	27833 w[1118]; Mi{ET1}CG9760[MB10130]
FBti0115915	27834 w[1118]; Mi{ET1}CG30043[MB10216]
FBti0115916	27835 w[1118]; Mi{ET1}MB10256
FBti0115917	27836 w[1118]; Mi{ET1}Osi12[MB10282]
FBti0115918	27837 w[1118]; Mi{ET1}CG34041[MB10285]
FBti0115919	27838 w[1118]; Mi{ET1}Mal-A3[MB10295]
FBti0115920	27839 w[1118]; Mi{ET1}CG14280[MB10320]
FBti0115921	27840 w[1118]; Mi{ET1}MB10340
FBti0115922	27841 w[1118]; Mi{ET1}CG4434[MB10362]
FBti0115923	27842 w[1118]; Mi{ET1}CG4935[MB10437]
FBti0115924	27843 w[1118]; Mi{ET1}gig[MB10497] CG7335[MB10497]
FBti0115925	27844 w[1118]; Mi{ET1}CG6490[MB10543]
FBti0115926	27845 w[1118]; Mi{ET1}MB10687
FBti0115927	27846 w[1118]; Mi{ET1}CG11425[MB10743]
FBti0115928	27847 w[1118]; Mi{ET1}CG42686[MB10828]
FBti0115929	27848 w[1118]; Mi{ET1}MB10839
FBti0115930	27849 w[1118]; Mi{ET1}CG32079[MB10846]
FBti0115931	27850 w[1118]; Mi{ET1}Edg91[MB10934]
FBti0115932	27851 w[1118]; Mi{ET1}Muc68E[MB10935]
FBti0115933	27852 w[1118]; Mi{ET1}MB10945
FBti0115934	27853 w[1118]; Mi{ET1}MB10967
FBti0115935	27854 w[1118]; Mi{ET1}MB11043

FBti0115936	27855 w[1118]; Mi{ET1}Or83c[MB11142]
FBti0115937	27856 w[1118]; Mi{ET1}MB11150
FBti0115938	27857 w[1118]; Mi{ET1}nompA[MB11221]/SM6a
FBti0115939	27858 w[1118]; Mi{ET1}MB11223
FBti0115940	27859 w[1118]; Mi{ET1}CG5819[MB11253]
FBti0115941	27860 w[1118]; Mi{ET1}CG14651[MB11257]
FBti0115942	27861 w[1118]; Mi{ET1}Ugt86Dh[MB11311]
FBti0115943	27862 w[1118]; Mi{ET1}trem[MB11321]
FBti0115944	27863 w[1118]; Mi{ET1}Cyp6g1[MB11394]
FBti0115945	27864 w[1118]; Mi{ET1}MB11438
FBti0115946	27865 w[1118]; Mi{ET1}Su(var)205[MB11439]
FBti0115947	27866 w[1118]; Mi{ET1}CG5265[MB11442]
FBti0115948	27867 w[1118]; Mi{ET1}CG34045[MB11449]
FBti0115949	27868 w[1118]; Mi{ET1}CG13407[MB11521] CG5778[MB11521]
FBti0115950	27869 w[1118]; Mi{ET1}ppk16[MB11536]
FBti0115951	27870 w[1118]; Mi{ET1}Scsα[MB11537]
FBti0115952	27871 w[1118]; Mi{ET1}Spn31A[MB11640]
FBti0115953	27872 w[1118]; Mi{ET1}CG14160[MB11680]
FBti0115954	27873 w[1118]; Mi{ET1}CG30222[MB11771]
FBti0115955	27874 w[1118]; Mi{ET1}CG10178[MB11816]
FBti0115956	27875 w[1118]; Mi{ET1}CG10469[MB11902]
FBti0115957	27876 w[1118]; Mi{ET1}MB12032
FBti0115958	27877 w[1118]; Mi{ET1}Cpr62Ba[MB12091]
FBti0115959	27878 w[1118]; Mi{ET1}CG5568[MB12106]
FBti0115960	27879 w[1118]; Mi{ET1}CG6118[MB12220]
FBti0115961	27880 w[1118]; Mi{ET1}Ccn[MB12229]
FBti0115962	27881 w[1118]; Mi{ET1}CG13293[MB12233]
FBti0115963	27882 w[1118]; Mi{ET1}CG30427[MB12237]
FBti0115964	27883 w[1118]; Mi{ET1}Gr64f[MB12243]
FBti0115965	27884 w[1118]; Mi{ET1}CG13727[MB12378]
FBti0115966	27885 w[1118]; Mi{ET1}CG9766[MB12417]
FBti0124338	28504 w[1118] Mi{ET1}dec-1[MB06340]
FBti0124339	27747 y[1]; Mi{ET1}bru-3[MB07091]
FBti0127439	29052 w[1118]; Mi{ET1}MB05855
FBti0127440	29053 w[1118]; Mi{ET1}CG12078[MB06909]
FBti0127441	29054 w[1118]; Mi{ET1}pip[MB07037]
FBti0127442	29055 w[1118]; Mi{ET1}Fps85D[MB08538]
FBti0127443	29056 w[1118]; Mi{ET1}CG13830[MB09645] CG6733[MB09645]
FBti0127444	29057 w[1118]; Mi{ET1}RpA-70[MB09710]

FBti0127445	29058 w[1118]; Mi{ET1}Pi4KIIα[MB09727]
FBti0127446	29059 w[1118]; Mi{ET1}CG9098[MB09740]
FBti0127447	29060 w[1118]; Mi{ET1}MB09750
FBti0127448	29061 w[1118]; Mi{ET1}MB09761
FBti0127449	29702 w[1118]; Mi{ET1}Mrp4[MB09770]
FBti0127450	29062 w[1118]; Mi{ET1}CG32441[MB09773]
FBti0127451	29063 w[1118]; Mi{ET1}CG1299[MB09811]
FBti0127452	29064 w[1118]; Mi{ET1}MB09819
FBti0127453	29065 w[1118]; Mi{ET1}Gr58b[MB09820]
FBti0127454	29066 w[1118]; Mi{ET1}MB09830
FBti0127455	29067 w[1118]; Mi{ET1}capaR[MB09834]/TM6C, Sb[1]
FBti0127456	29068 w[1118] Mi{ET1}MB09853
FBti0127457	29069 w[1118] Mi{ET1}MB09856
FBti0127458	29070 w[1118]; Mi{ET1}NepYr[MB09874]
FBti0127459	29071 w[1118]; Mi{ET1}CG6931[MB09876]
FBti0127460	29072 w[1118]; Mi{ET1}MB09900
FBti0127461	29073 w[1118]; Mi{ET1}trio[MB09917]
FBti0127462	29074 w[1118]; Mi{ET1}CcapR[MB09926]
FBti0127463	29075 w[1118]; Mi{ET1}CG8834[MB09927]
FBti0127464	29076 w[1118] Mi{ET1}CG1636[MB09941]
FBti0127465	29077 w[1118]; Mi{ET1}MB09990
FBti0127466	29078 w[1118]; Mi{ET1}MB09995
FBti0127467	29079 w[1118]; Mi{ET1}MB10010
FBti0127468	29080 w[1118]; Mi{ET1}MB10017
FBti0127469	29081 w[1118]; Mi{ET1}beat-Ia[MB10019]
FBti0127471	29082 w[1118]; Mi{ET1}CG10170[MB10022]
FBti0127472	29083 w[1118]; Mi{ET1}MB10032a Mi{ET1}MB10032b
FBti0127474	29084 w[1118]; Mi{ET1}MB10036
FBti0127475	29085 w[1118]; Mi{ET1}CG11413[MB10057] CG4622[MB10057]
FBti0127476	29086 w[1118]; Mi{ET1}CG8646[MB10060]
FBti0127477	29087 w[1118]; Mi{ET1}CG4925[MB10061]
FBti0127478	29088 w[1118]; Mi{ET1}CG6830[MB10063]
FBti0127479	29089 w[1118]; Mi{ET1}α-Spec[MB10065]
FBti0127480	29090 w[1118]; Mi{ET1}Ir67b[MB10077]
FBti0127481	29091 w[1118]; Mi{ET1}MB10099
FBti0127482	29092 w[1118]; Mi{ET1}cnc[MB10127]
FBti0127483	29093 w[1118]; Mi{ET1}MB10133
FBti0127484	29094 w[1118]; Mi{ET1}MB10134
FBti0127485	29095 w[1118]; Mi{ET1}MB10136

FBti0127486	29096 w[1118]; Mi{ET1}CG31102[MB10157]
FBti0127487	29097 w[1118]; Mi{ET1}MB10161
FBti0127488	29098 w[1118]; Mi{ET1}MB10175
FBti0127489	29099 w[1118]; Mi{ET1}MB10204
FBti0127490	29100 w[1118]; Mi{ET1}tst[MB10212]/TM6C, Sb[1]
FBti0127491	29101 Mi{ET1}Scgδ[MB10218] w[1118]
FBti0127492	29102 w[1118] Mi{ET1}CG7423[MB10222]
FBti0127493	29103 w[1118]; Mi{ET1}MB10231
FBti0127494	29104 w[1118]; Mi{ET1}Dbx[MB10238]
FBti0127495	29105 w[1118]; Mi{ET1}MB10240
FBti0127496	29106 w[1118]; Mi{ET1}CG32462[MB10243]
FBti0127497	29107 w[1118]; Mi{ET1}Ast[MB10261]
FBti0127498	29108 w[1118]; Mi{ET1}CG5359[MB10284]
FBti0127499	29109 w[1118]; Mi{ET1}Efhc1.2[MB10286]
FBti0127500	29110 w[1118]; Mi{ET1}CG42669[MB10299]
FBti0127501	29111 w[1118]; Mi{ET1}MB10310
FBti0127502	29112 w[1118]; Mi{ET1}CG15236[MB10311]
FBti0127503	29113 w[1118]; Mi{ET1}MB10323
FBti0127504	29114 w[1118]; Mi{ET1}CG13501[MB10330]
FBti0127505	29115 w[1118]; Mi{ET1}CG32473[MB10347]
FBti0127506	29116 w[1118]; Mi{ET1}CG12950[MB10368]
FBti0127507	29117 w[1118]; Mi{ET1}grim[MB10379]
FBti0127508	29118 w[1118]; Mi{ET1}Dgk[MB10383]
FBti0127509	29119 w[1118]; Mi{ET1}MB10389
FBti0127510	29120 w[1118]; Mi{ET1}CG12880[MB10396]
FBti0127511	29121 w[1118]; Mi{ET1}CG42613[MB10397]
FBti0127512	29122 w[1118]; Mi{ET1}beat-Ib[MB10402]
FBti0127513	29123 w[1118]; Mi{ET1}al[MB10407]
FBti0127514	29124 w[1118]; Mi{ET1}MB10442
FBti0127515	29125 w[1118]; Mi{ET1}MB10444
FBti0127516	29126 w[1118]; Mi{ET1}CG12017[MB10464]
FBti0127517	29127 w[1118]; Mi{ET1}QC[MB10475]
FBti0127518	29128 w[1118]; Mi{ET1}CG31288[MB10489]
FBti0127519	29129 w[1118]; Mi{ET1}Dh44-R2[MB10503]
FBti0127520	29130 w[1118]; Mi{ET1}CG4839[MB10509]
FBti0127521	29131 w[1118]; Mi{ET1}CG44433[MB10518]
FBti0127522	29132 w[1118]; Mi{ET1}MB10540
FBti0127523	29133 w[1118]; Mi{ET1}MB10550
FBti0127524	29134 w[1118]; Mi{ET1}trpl[MB10553]

FBti0127525	29135 w[1118]; Mi{ET1}MB10568
FBti0127526	29136 w[1118]; Mi{ET1}CG8680[MB10571]
FBti0127527	29137 w[1118]; Mi{ET1}MB10572
FBti0127528	29138 w[1118]; Mi{ET1}ome[MB10610] CG17177[MB10610]
FBti0127529	29139 Mi{ET1}MB10623 w[1118]
FBti0127530	29140 w[1118]; Mi{ET1}Galk[MB10638]
FBti0127531	29141 w[1118]; Mi{ET1}CG5532[MB10648]
FBti0127532	29142 w[1118]; Mi{ET1}CG32071[MB10652]
FBti0127533	29143 w[1118]; Mi{ET1}GEFmeso[MB10683]
FBti0127534	29144 w[1118]; Mi{ET1}MB10691
FBti0127535	29145 w[1118]; Mi{ET1}CG14118[MB10699]
FBti0127536	29146 w[1118]; Mi{ET1}CG13248[MB10708]
FBti0127537	29147 w[1118]; Mi{ET1}CG34436[MB10721]
FBti0127538	29148 w[1118]; Mi{ET1}MB10722
FBti0127539	29149 w[1118]; Mi{ET1}MB10730
FBti0127540	29150 w[1118]; Mi{ET1}Ten-m[MB10734]/TM6C, Sb[1]
FBti0127541	29151 w[1118]; Mi{ET1}CG18109[MB10748]
FBti0127542	29152 w[1118]; Mi{ET1}CG15394[MB10758]
FBti0127543	29153 w[1118]; Mi{ET1}unc-104[MB10763]
FBti0127544	29154 w[1118]; Mi{ET1}MB10770
FBti0127545	29155 w[1118]; Mi{ET1}MB10785
FBti0127546	29156 w[1118]; Mi{ET1}MB10803
FBti0127547	29157 w[1118]; Mi{ET1}Gαf[MB10810]
FBti0127548	29158 w[1118]; Mi{ET1}MB10830
FBti0127549	29159 w[1118]; Mi{ET1}WDR79[MB10832]
FBti0127550	29160 w[1118]; Mi{ET1}olf186-F[MB10834] CG30323[MB10834]
FBti0127551	29161 w[1118]; Mi{ET1}CG34437[MB10842]
FBti0127552	29162 w[1118]; Mi{ET1}CG8586[MB10855]
FBti0127553	29163 w[1118]; Mi{ET1}CG7194[MB10883]
FBti0127554	29164 w[1118]; Mi{ET1}Fife[MB10889]
FBti0127555	29165 w[1118]; Mi{ET1}MB10901
FBti0127556	29166 w[1118]; Mi{ET1}RtGEF[MB10902]
FBti0127557	29167 w[1118]; Mi{ET1}MB10910
FBti0127558	29168 w[1118]; Mi{ET1}CG13455[MB10922]
FBti0127559	29169 w[1118]; Mi{ET1}Cyp313a5[MB10923]
FBti0127560	29170 w[1118]; Mi{ET1}CG15537[MB10928]
FBti0127561	29171 w[1118]; Mi{ET1}nyo[MB10929]
FBti0127562	29172 w[1118]; Mi{ET1}CG31690[MB10962]
FBti0127563	29173 w[1118]; Mi{ET1}MB10977

FBti0127564	29174 w[1118]; Mi{ET1}MB10992
FBti0127565	29175 w[1118]; Mi{ET1}CG10086[MB11019]
FBti0127566	29176 w[1118]; Mi{ET1}MB11023
FBti0127567	29177 w[1118]; Mi{ET1}Snx1[MB11025]
FBti0127568	29178 w[1118]; Mi{ET1}MB11028
FBti0127569	29179 w[1118]; Mi{ET1}ppk12[MB11059]
FBti0127570	29180 w[1118]; Mi{ET1}Hsc70-1[MB11077]
FBti0127571	29181 w[1118]; Mi{ET1}Obp84a[MB11081] CG10050[MB11081]
FBti0127572	29182 w[1118]; Mi{ET1}MB11135
FBti0127573	29183 w[1118]; Mi{ET1}MB11156
FBti0127574	29184 w[1118]; Mi{ET1}Gfrl[MB11189]
FBti0127575	29185 w[1118]; Mi{ET1}CG30177[MB11215]
FBti0127576	29186 w[1118]; Mi{ET1}CG12560[MB11224]
FBti0127577	29187 w[1118]; Mi{ET1}ome[MB11247]
FBti0127578	29188 w[1118]; Mi{ET1}MB11266
FBti0127579	29189 w[1118]; Mi{ET1}MB11267
FBti0127580	29190 w[1118]; Mi{ET1}CG32333[MB11273]
FBti0127581	29191 w[1118]; Mi{ET1}Irc[MB11278]
FBti0127582	29192 w[1118]; Mi{ET1}MB11286
FBti0127583	29193 w[1118]; Mi{ET1}CG42399[MB11288]
FBti0127584	29194 w[1118]; Mi{ET1}CG42784[MB11294]
FBti0127585	29195 w[1118]; Mi{ET1}serp[MB11301]
FBti0127586	29196 w[1118]; Mi{ET1}CG17147[MB11340]
FBti0127587	29197 w[1118]; Mi{ET1}CG12917[MB11343]
FBti0127588	29198 w[1118]; Mi{ET1}MB11350
FBti0127589	29199 w[1118]; Mi{ET1}MB11354
FBti0127590	29200 w[1118]; Mi{ET1}CG7573[MB11355]
FBti0127591	29201 w[1118]; Mi{ET1}Cha[MB11366] CG7714[MB11366]
FBti0127592	29202 w[1118]; Mi{ET1}asp[MB11367]/TM6C, Sb[1]
FBti0127593	29203 w[1118]; Mi{ET1}MB11400
FBti0127594	29204 w[1118]; Mi{ET1}kek4[MB11415]
FBti0127595	29205 w[1118]; Mi{ET1}CG14330[MB11420]
FBti0127596	29206 w[1118]; Mi{ET1}MB11421
FBti0127597	29207 w[1118]; Mi{ET1}Ptr[MB11426]
FBti0127598	29208 w[1118]; Mi{ET1}CG4288[MB11427]
FBti0127599	29209 w[1118]; Mi{ET1}CG18213[MB11443]
FBti0127600	29210 w[1118]; Mi{ET1}CG4080[MB11450]
FBti0127601	29211 w[1118]; Mi{ET1}CanA1[MB11454]
FBti0127602	29212 w[1118]; Mi{ET1}CG8334[MB11462]

FBti0127603	29213 w[1118]; Mi{ET1}MB11484
FBti0127604	29214 w[1118]; Mi{ET1}Ddr[MB11489]
FBti0127605	29215 w[1118]; Mi{ET1}bru-3[MB11495]
FBti0127606	29216 w[1118]; Mi{ET1}CG13871[MB11500] CG8920[MB11500]
FBti0127607	29217 w[1118]; Mi{ET1}Pop2[MB11505]/TM6C, Sb[1]
FBti0127608	29218 w[1118]; Mi{ET1}ik2[MB11506]/SM6a
FBti0127609	29219 w[1118]; Mi{ET1}MB11512
FBti0127610	29220 w[1118]; Mi{ET1}MB11518
FBti0127611	29221 w[1118]; Mi{ET1}CG11317[MB11524]
FBti0127612	29222 w[1118] Mi{ET1}RabX2[MB11526] CG32683[MB11526]
FBti0127613	29223 w[1118]; Mi{ET1}MB11533
FBti0127614	29224 w[1118]; Mi{ET1}Eig71Ed[MB11550]
FBti0127615	29225 w[1118]; Mi{ET1}MB11551
FBti0127616	29226 w[1118]; Mi{ET1}CG11927[MB11553]/SM6a
FBti0127617	29227 w[1118] Mi{ET1}spheroide[MB11555] CG4678[MB11555]
FBti0127618	29228 w[1118]; Mi{ET1}RpL14[MB11564]
FBti0127619	29229 w[1118]; Mi{ET1}CG34183[MB11592]/SM6a
FBti0127620	29230 w[1118]; Mi{ET1}CG1550[MB11597]
FBti0127621	29231 w[1118]; Mi{ET1}r2d2[MB11618]/SM6a
FBti0127622	29232 w[1118]; Mi{ET1}CG3570[MB11625] CG4707[MB11625]
FBti0127623	29233 w[1118]; Mi{ET1}tim[MB11632]
FBti0127624	29234 w[1118]; Mi{ET1}MB11633
FBti0127625	29235 w[1118]; Mi{ET1}MB11641
FBti0127626	29236 w[1118]; Mi{ET1}nAcRα-34E[MB11647]/SM6a
FBti0127627	29237 w[1118]; Mi{ET1}Sulf1[MB11661]
FBti0127628	29238 w[1118]; Mi{ET1}Cyp4d21[MB11663]
FBti0127629	29239 w[1118]; Mi{ET1}Tep3[MB11679]/SM6a
FBti0127630	29240 w[1118]; Mi{ET1}CG15023[MB11692]
FBti0127631	29241 w[1118]; Mi{ET1}vas[MB11697]
FBti0127632	29242 w[1118]; Mi{ET1}MB11706/TM6C, Sb[1]
FBti0127633	29243 w[1118]; Mi{ET1}MB11710
FBti0127634	29244 w[1118]; Mi{ET1}msl-3[MB11711]
FBti0127635	29245 w[1118]; Mi{ET1}MB11712/SM6a
FBti0127636	29246 w[1118]; Mi{ET1}sim[MB11720]
FBti0127637	29247 w[1118]; Mi{ET1}MB11729
FBti0127638	29248 w[1118]; Mi{ET1}CG10019[MB11741]
FBti0127639	29249 w[1118]; Mi{ET1}MB11751
FBti0127640	29250 w[1118]; Mi{ET1}PGAP5[MB11752]/SM6a
FBti0127641	29251 w[1118]; Mi{ET1}CG7025[MB11758]

FBti0127642	29252 w[1118]; Mi{ET1}pk[MB11776] CG30384[MB11776]
FBti0127643	29253 w[1118]; Mi{ET1}MB11781
FBti0127644	29254 w[1118]; Mi{ET1}CG15923[MB11785]
FBti0127645	29255 w[1118]; Mi{ET1}fred[MB11793]
FBti0127646	29256 w[1118]; Mi{ET1}dtr[MB11825]
FBti0127647	29257 w[1118]; Mi{ET1}CG42796[MB11858]
FBti0127648	29258 w[1118]; Mi{ET1}CG31789[MB11884]
FBti0127649	29259 w[1118]; Mi{ET1}MB11896
FBti0127650	29260 w[1118]; Mi{ET1}mle[MB11906]
FBti0127651	29261 w[1118]; Mi{ET1}MB11914
FBti0127652	29262 w[1118]; Mi{ET1}fon[MB11923]/SM6a
FBti0127653	29263 w[1118]; Mi{ET1}MB11934
FBti0127654	29264 w[1118]; Mi{ET1}Toll-9[MB11953]
FBti0127655	29265 w[1118]; Mi{ET1}fdl[MB11956] s-cup[MB11956]
FBti0127656	29266 w[1118]; Mi{ET1}CCHa1[MB11962]
FBti0127657	29267 w[1118]; Mi{ET1}mas[MB11963]/TM6C, Sb[1]
FBti0127658	29268 w[1118]; Mi{ET1}Adgf-A[MB11970] Msi[MB11970] CG32181[MB11970]
FBti0127659	29269 w[1118]; Mi{ET1}MB11987
FBti0127660	29270 w[1118]; Mi{ET1}Taf11[MB11995]
FBti0127661	29271 w[1118]; Mi{ET1}MB11997
FBti0127662	29272 w[1118]; Mi{ET1}Fs[MB12011]
FBti0127663	29273 w[1118]; Mi{ET1}CG4887[MB12014]
FBti0127664	29274 w[1118]; Mi{ET1}MB12038
FBti0127665	29275 w[1118]; Mi{ET1}Npc2e[MB12074]
FBti0127666	29276 w[1118]; Mi{ET1}GABA-B-R1[MB12092] CG33310[MB12092]
FBti0127667	29277 w[1118]; Mi{ET1}CG33690[MB12096]
FBti0127668	29278 w[1118]; Mi{ET1}Asph[MB12115]
FBti0127669	29279 w[1118]; Mi{ET1}bc10[MB12117]
FBti0127670	29280 w[1118]; Mi{ET1}CG42284[MB12120]
FBti0127671	29281 w[1118]; Mi{ET1}S-Lap8[MB12123]/SM6a
FBti0127672	29282 w[1118]; Mi{ET1}MB12138
FBti0127673	29283 w[1118] Mi{ET1}cngl[MB12199]
FBti0127674	29284 w[1118]; Mi{ET1}Orct2[MB12221]
FBti0127675	29285 w[1118]; Mi{ET1}MB12252
FBti0127676	29286 w[1118]; Mi{ET1}so[MB12256]
FBti0127677	29287 w[1118]; Mi{ET1}MB12259
FBti0127678	29288 w[1118]; Mi{ET1}Ndg[MB12298]
FBti0127679	29289 w[1118]; Mi{ET1}Cpr76Bc[MB12303]
FBti0127680	29290 w[1118]; Mi{ET1}CG9706[MB12310]

FBti0127681	29291 w[1118]; Mi{ET1}dan[MB12311] lobo[MB12311]
FBti0127682	29292 w[1118]; Mi{ET1}Pdh[MB12341]
FBti0127683	29293 w[1118]; Mi{ET1}MB12346
FBti0127684	29294 w[1118]; Mi{ET1}fal[MB12347]
FBti0127685	29295 w[1118]; Mi{ET1}CadN[MB12355]/SM6a
FBti0127686	29296 w[1118]; Mi{ET1}MB12356
FBti0127687	29297 w[1118]; Mi{ET1}frac[MB12360]
FBti0127688	29298 w[1118] Mi{ET1}CG33223[MB12372]
FBti0127689	29299 w[1118]; Mi{ET1}CG8833[MB12392]
FBti0128205	29885 w[1118]; Mi{ET1}Gcn5[MB09741]/TM6C, Sb[1]
FBti0128206	29886 w[1118]; Mi{ET1}Npc1a[MB09749]/SM6a
FBti0128207	29887 w[1118]; Mi{ET1}MB09944
FBti0128208	29888 w[1118]; Mi{ET1}tefu[MB09945]/TM6C, Sb[1]
FBti0128209	29889 w[1118]; Mi{ET1}CG14995[MB09963]
FBti0128210	29890 w[1118] Mi{ET1}MB10049
FBti0128211	29891 w[1118]; Mi{ET1}CG14835[MB10076]
FBti0128212	29892 w[1118]; Mi{ET1}CG32091[MB10254]
FBti0128213	29893 w[1118]; Mi{ET1}hbs[MB10413]
FBti0128214	29894 w[1118]; Mi{ET1}MB10492
FBti0128215	29895 w[1118]; Mi{ET1}jp[MB10539]
FBti0128216	29896 w[1118] Mi{ET1}if[MB10541]
FBti0128217	29897 w[1118]; Mi{ET1}CG13252[MB10555]
FBti0128218	29898 w[1118]; Mi{ET1}CG8562[MB10558]
FBti0128219	29899 w[1118]; Mi{ET1}CG14526[MB10591] CG33203[MB10591]
FBti0128220	29900 w[1118]; Mi{ET1}MB10660
FBti0128221	29901 w[1118]; Mi{ET1}tn[MB10738]
FBti0128222	29902 w[1118]; Mi{ET1}MB10762
FBti0128223	29903 w[1118]; Mi{ET1}CG6071[MB10956]
FBti0128224	29904 w[1118]; Mi{ET1}Zasp66[MB10984]
FBti0128225	29905 Mi{ET1}CG3038[MB11095] w[1118]
FBti0128227	29907 w[1118]; Mi{ET1}MB11154
FBti0128228	29908 w[1118]; Mi{ET1}MB11208
FBti0128229	29909 w[1118]; Mi{ET1}MB11260
FBti0128230	29910 w[1118]; Mi{ET1}CG10936[MB11295] Oxp[MB11295]
FBti0128231	29911 w[1118]; Mi{ET1}S-Lap4[MB11296]
FBti0128232	29912 w[1118]; Mi{ET1}CG3257[MB11332]
FBti0128233	29913 w[1118]; Mi{ET1}ara[MB11365]/TM6C, Sb[1]
FBti0128234	29914 w[1118]; Mi{ET1}CG11221[MB11376]
FBti0128235	29915 w[1118]; Mi{ET1}CG10623[MB11441]/SM6a

FBti0128236	29916 w[1118]; Mi{ET1}MB11453
FBti0128237	29917 w[1118]; Mi{ET1}Sytβ[MB11464]
FBti0128238	29918 w[1118]; Mi{ET1}slo[MB11481]
FBti0128239	29919 w[1118] Mi{ET1}Myo10A[MB11485]
FBti0128240	29920 w[1118]; Mi{ET1}MB11493
FBti0128241	29921 w[1118]; Mi{ET1}CG14736[MB11513]
FBti0128242	29922 w[1118]; Mi{ET1}MB11516
FBti0128243	29923 w[1118]; Mi{ET1}Sur-8[MB11559]
FBti0128244	29924 w[1118]; Mi{ET1}star1[MB11579]
FBti0128245	29925 w[1118]; Mi{ET1}lqfR[MB11643]/TM6C, Sb[1]
FBti0128246	29926 w[1118]; Mi{ET1}CG17684[MB11659]
FBti0128247	29927 w[1118]; Mi{ET1}MB11674
FBti0128248	29928 w[1118]; Mi{ET1}CG10006[MB11703]
FBti0128249	29929 w[1118]; Mi{ET1}mirr[MB11790]
FBti0128250	29930 w[1118]; Mi{ET1}CG7309[MB11863]
FBti0128251	29931 Mi{ET1}MB11933 w[1118]
FBti0128252	29932 w[1118] Mi{ET1}CG1463[MB11940]
FBti0128253	29933 Mi{ET1}G9a[MB11975] w[1118]
FBti0128254	29934 w[1118]; Mi{ET1}MB11992
FBti0128255	29935 w[1118] Mi{ET1}MB11996
FBti0128256	29936 w[1118]; Mi{ET1}CG6241[MB11999]/TM6C, Sb[1]
FBti0128257	29937 Mi{ET1}MB12027 w[1118]
FBti0128258	29938 w[1118] Mi{ET1}MB12063
FBti0128259	29939 w[1118]; Mi{ET1}Dhc62B[MB12102]
FBti0128260	29940 Mi{ET1}CG3711[MB12128] w[1118]
FBti0128261	29941 w[1118]; Mi{ET1}CG14509[MB12140]
FBti0128262	29942 w[1118]; Mi{ET1}Smyd4[MB12148]
FBti0128263	30000 w[1118]; Mi{ET1}CG14128[MB12149]/TM6C, Sb[1]
FBti0128264	29943 w[1118]; Mi{ET1}MB12150
FBti0128265	29944 w[1118]; Mi{ET1}NijC[MB12178]
FBti0128266	29945 w[1118]; Mi{ET1}MB12211
FBti0128267	29946 w[1118]; Mi{ET1}Nelf-E[MB12251] CG43078[MB12251]
FBti0128268	29998 w[1118]; Mi{ET1}CG9377[MB12286]
FBti0128269	29947 w[1118] Mi{ET1}Gr10a[MB12324]
FBti0128270	29948 w[1118] Mi{ET1}Scamp[MB12334]
